# Anthroponumbers.org: A quantitative database of human impacts on Planet Earth

**DOI:** 10.1016/j.patter.2022.100552

**Published:** 2022-08-03

**Authors:** Griffin Chure, Rachel A. Banks, Avi I. Flamholz, Nicholas S. Sarai, Mason Kamb, Ignacio Lopez-Gomez, Yinon Bar-On, Ron Milo, Rob Phillips

**Affiliations:** 1 Department of Biology, Stanford University, Stanford, CA, USA; 2 Department of Applied Physics, California Institute of Technology, Pasadena, CA, USA; 3 Division of Biology and Biological Engineering, California Institute of Technology, Pasadena, CA, USA; 4 Resnick Sustainibility Institute, California Institute of Technology, Pasadena, CA, USA; 5 Chan-Zuckerberg BioHub, San Francisco, CA, USA; 6 Division of Chemistry and Chemical Engineering, California Institute of Technology, Pasadena, CA, USA; 7 Department of Environmental Science and Engineering, California Institute of Technology, Pasadena, CA, USA; 8 Department of Plant and Environmental Sciences, Weizmann Institute of Science, Rehovot, Israel; 9 Department of Physics, California Institute of Technology, Pasadena, CA, USA

**Keywords:** Anthropocene, database, human impacts, climate change, Earth-human system, global ecology

## Abstract

The Human Impacts Database (www.anthroponumbers.org) is a curated, searchable resource housing quantitative data relating to the diverse anthropogenic impacts on our planet, with topics ranging from sea-level rise to livestock populations, greenhouse gas emissions, fertilizer use, and beyond. Each entry in the database reports a quantitative value (or a time series of values) along with clear referencing of the primary source, the method of measurement or estimation, an assessment of uncertainty, and links to the underlying data, as well as a permanent identifier called a Human Impacts ID (HuID). While there are other databases that house some of these values, they are typically focused on a single topic area, like energy usage or greenhouse gas emissions. The Human Impacts Database facilitates access to carefully curated data, acting as a quantitative resource pertaining to the myriad ways in which humans have an impact on the Earth, for practicing scientists, the general public, and those involved in education for sustainable development alike. We outline the structure of the database, describe our curation procedures, and use this database to generate a graphical summary of the current state of human impacts on the Earth, illustrating both their numerical values and their intimate interconnections.

## Highlights

• We present a holistic view of the many ways humans alter Earth at a global scale
• We consider how these global quantities vary across geography
• We further explore the time- and population-dependent dynamics of these impacts
• We enumerate and describe key properties associated with each entry in the database

## The bigger picture

Over the last 10,000 years, human activities have transformed Earth through farming, forestry, mining, and industry. The complex results of these activities are now observed and quantified as “human impacts” on Earth’s atmosphere, oceans, biosphere, and geochemistry. While myriad studies have explored facets of human impacts on the planet, they are necessarily technical and often highly focused. Thus, finding reliable quantitative information requires a significant investment of time to assess each quantity and associated uncertainty. We present the Human Impacts Database (www.anthroponumbers.org), which houses a diverse array of such quantities. We review a subset of these values and how they help build intuition for understanding the Earth-human system. While collation alone does not tell us how to best ameliorate human impacts, we contend that any future plans should be made in light of a quantitative understanding of the interconnected ways in which humans influence the planet.

## Graphical abstract

**Figure:**
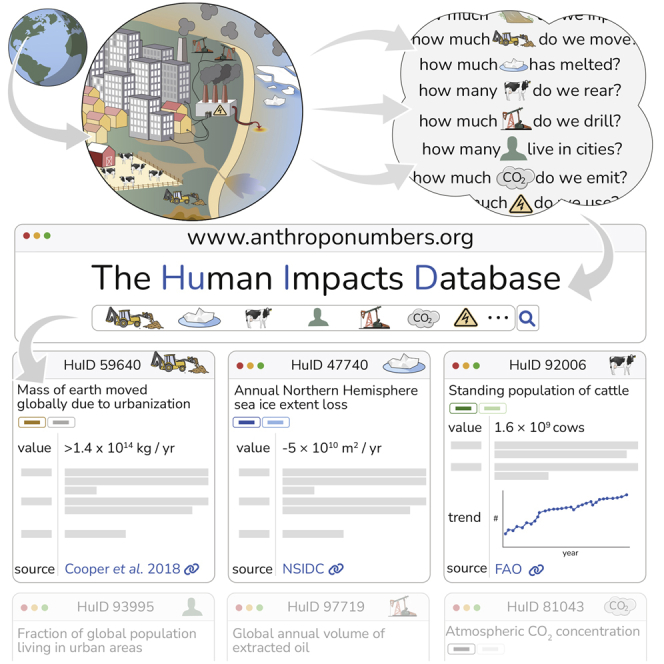

## Teaser

The environmental impacts of human action on Earth are being felt on many fronts. Despite our deep knowledge of these impacts, finding reliable quantitative information is burdensome, often requiring domain expertise and programmatic acumen. We present the Human Impacts Database, which houses a diverse array of quantities regarding human impacts, making them easily accessible and searchable. We use this database to present a broad view of the Anthropocene, exploring the global magnitudes, spatial dependence, and temporal dynamics of human impacts.

## Introduction

One of the most important scientific developments of the last two centuries is the realization that the evolution of Earth is deeply intertwined with the evolution of life. Perhaps the most famous example of this intimate relationship is the large-scale oxygenation of Earth’s atmosphere following the emergence of photosynthesis.^1^ This dramatic change in the composition of the atmosphere is believed to have caused a massive extinction, as the biosphere was not adapted to an oxygenated atmosphere.^2^, ^3^, ^4^ Over the past 10,000 years, humans have likewise transformed the planet, directly affecting the rise and fall of ecosystems,^5^, ^6^, ^7^, ^8^, ^9^, ^10^, ^11^, ^12^, ^13^ the pH and surface temperature of the oceans,^14^^,^^15^ the composition of terrestrial biological and human-made mass,^16^^,^^17^ the planetary albedo and ice cover,^18^, ^19^, ^20^, ^21^, ^22^, ^23^, ^24^, ^25^, ^26^, ^27^ and the chemistry of the atmosphere,^28^, ^29^, ^30^, ^31^, ^32^, ^33^ to name just a few examples. The breadth of human impacts on the planet is so diverse that it touches on nearly every facet of the Earth system and every scientific discipline.

Technological advances in remote sensing, precision measurement, and computational power have made it possible to measure these anthropogenic impacts with unprecedented depth and resolution. However, as scientists with different training use distinct methods for measurement and analysis, report data in different units and formats, and use nomenclature differently, these studies can be very challenging to understand and relate to one another. Even seemingly simple questions such as “how much water do humans use?” can be difficult to answer when search engines are not optimized for finding numeric data, and a search of the scientific literature yields an array of complicated analyses with different units, varying definitions about what constitutes water use, and distinct approaches to quantifying flows. This problem persists beyond the primary scientific literature, as governmental, intergovernmental, and industry datasets can be similarly tricky and laborious to interpret.

Writing from California, as several of the authors are, where we now have a “wildfire season” and a multi-decadal drought,^34^^,^^35^ we wanted to develop a deeper understanding of the ways in which human activities might have produced such dramatic and consequential changes in our local and global environment. In pursuit of basic understanding, we asked many questions, like “how much water and land do humans use?” and “how much methane is emitted annually?” In our search for answers, even when the question is well defined (as is the case for methane emissions), we often encountered the same challenges: disparate technical studies written for expert audiences must be understood, evaluated, and synthesized just to answer simple questions. It seemed to us that a referenced compendium of “things we already know,” akin to the *CRC Handbook of Chemistry and Physics*, would be very useful for us and others.

In building the Human Impacts Database, we took inspiration from our previous experience building and using the BioNumbers Database^36^ (https://bionumbers.hms.harvard.edu), a compendium of quantitative values relating to cell and organismal biology. Over the past decade, the BioNumbers Database has become a widely accessed resource that serves not only as an index of biological numbers, but also as a means of finding relevant primary literature, learning about methods of measurement, and teaching basic concepts in cell biology.^37^ We believe that a centralized, searchable database for quantitative data encompassing the breadth of human impacts on Earth would be similarly transformative for researchers, students, and the interested public. While reading an entry in the Human Impacts Database is not a replacement for reading the primary literature, the database serves as a resource to expedite the process of finding quantitative data and exploring their interconnection. Importantly, we do not put forward projected scenarios or specific policy proposals for combating anthropogenic effects on Earth. However, we are convinced that such proposals should be evaluated in the light of a comprehensive and quantitative understanding of the Earth-human system.

## Results

### Finding and compiling numbers from scientific literature, governmental and non-governmental reports, and industrial datasets

We have established the Human Impacts Database (http://anthroponumbers.org) as a repository for the rapid discovery of quantities describing the Earth-human system. We here provide a more complete description of the database structure, the values it holds, and the stories it tells us about how humans affect the Earth. As of this writing, the database holds > 300 unique and manually curated entries covering a breadth of data sources, including primary scientific literature, governmental and non-governmental reports, and industrial communiques. Before it is added to the database and made public, each entry is vetted extensively by the administrators (see Note S1 for detailed curation procedures). Included in each entry is a summary of the method by which it was determined, an assessment of the corresponding uncertainty, and an explicit statement of any known caveats important for interpretation of the data. While these ≈ 300 entries include those we consider to be essential for a quantitative understanding of human impacts on Earth, it is not an exhaustive list. This database will continue to grow and evolve as more data become publicly released, our understanding of the human-Earth system improves, and members of the scientific community suggest values to be added.

**Figure 1:**
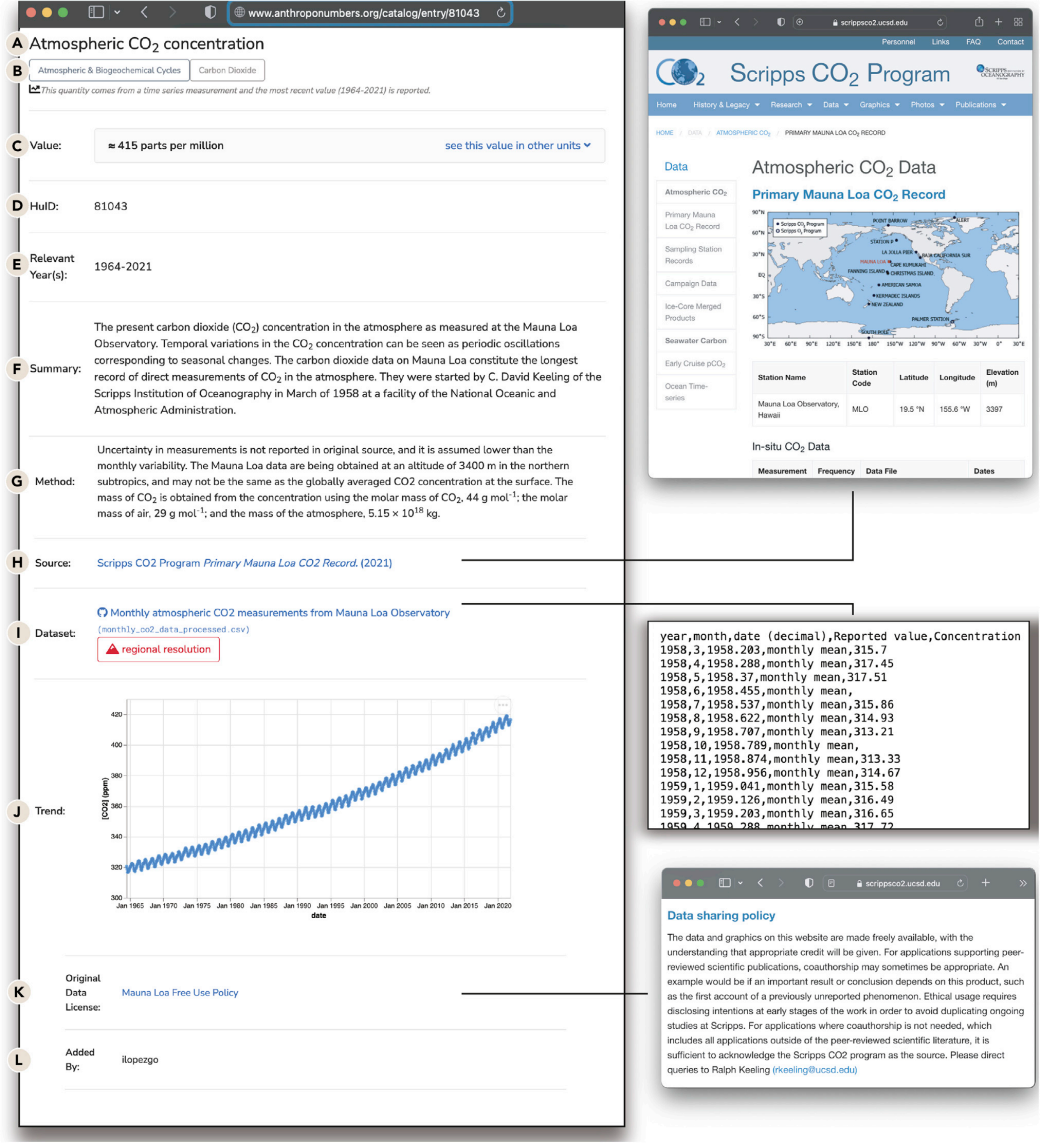
A representative entry in the Human Impacts Database (A–I) The entry page for HuID 81043, “Atmospheric CO_2_ concentration,” is diagrammed with important features highlighted. Each entry in the Human Impacts Database has (A) a name, (B) a primary and secondary categorization, (C) the numerical value with other units when appropriate, (D) a five-digit permanent numeric identifier, (E) the years for which the measurement was determined, (F) a brief summary of the quantity, (G) the method of determination, (H) a link to the source data, and (I) a link to a processed version of the data saved as a .csv file. When possible, a time series of the data is presented. (K) Every entry in the database also has a statement of the data use protection associated with the relevant data. When possible, this links directly to the data protection statement from the original source. In other cases, it points to the formal definition of the license by a disinterested third party. (L) Finally, each entry lists the username of the administrator who curated the quantity. Their contact information is available on the anthroponumbers.org “About” page.

Figure 1 shows the Human Impacts Database Entry for perhaps the most emblematic anthropogenic impact: the standing atmospheric CO_2_ concentration. The first two components of an entry are the quantity title and its assigned category and subcategory (Figures 1A and 1B). Primary categorization falls into one of five classes: “land,” “water,” “energy,” “flora & fauna,” and “atmospheric & biogeochemical cycles.” Of course, these categories are broad, and entries can be associated with several categories. For this reason, each entry is also assigned a narrower “subcategory,” such as “agriculture,” “urbanization,” or “carbon dioxide.” While this categorization is not meant to be exhaustive, and many other schemes could be implemented, we found that this organization allowed us to quickly browse and identify quantities of interest.

Following the title and categorization, we report the measured atmospheric CO_2_ concentration. This corresponds to the most recent reported measurement, which is, as of this writing, roughly 416 parts per million (ppm) in 2021 (Figure 1C). Importantly, we report an approximate value for the CO_2_ concentration rather than a precise value to many significant digits. While the most recent entry in the linked dataset (Figure 1I) gives a monthly average value of 416.43 ppm for December of 2021, this value does not account for error in the measurement, fluctuations throughout December, or seasonal oscillations in atmospheric CO_2_. Therefore, we report a rounded value of 416 ppm. CO_2_ measurements are quite accurate, but other measurements and inferences recorded in the Human Impacts Database are less so. We therefore strive to give an assessment of the uncertainty for all values. This can be in the form of a confidence interval, as for the entry for the global mean sea-level rise since 1900 due to thermal expansion, which reports a 90% confidence interval, or bounds on the value, as for the number of contemporary animal extinctions since 1500 CE, which reports only a lower bound. In addition to error assessment, we also aim to provide legible units for all entries. Although atmospheric CO_2_ is commonly reported in ppm units, we also report this value in other equivalent units, including the mole and mass fractions of CO_2_ and the total mass of CO_2_ in the atmosphere in kg CO_2_ (Figure 1C). Whenever possible, entries will report values in multiple units to make quantities accessible to readers coming from diverse backgrounds. Furthermore, in many cases, the global value is aggregated from local measurements. We flag entries for which regional data broadly defined are available in the database GitHub repository.

Following the numerical value is the permanent Human Impacts Database identifier, which we abbreviate as HuID (Figure 1D). The HuID is a randomly generated five-digit integer that serves as a permanent and static identifier that can be used for in-line referencing. Rather than identifying a single value, we consider the HuID a pointer to a particular *entry*, so that HuID 81043 can be used to reference the atmospheric CO_2_ concentration in 2021 and 1980 (Figure 1E). For example, to reference the present-day atmospheric CO_2_ concentration, one could report the value as “≈ 416 ppm (HuID 81043:2021).” In addition, since each entry comes from a single source, we may have more than one HuID reporting similar quantities. For example, HuIDs 69674 and 72086 report recent measurements of the temperature of the upper ocean.

The “Summary” field (Figure 1F) gives a succinct description of the quantity and its relationship to “human impacts” broadly construed, along with other pertinent information. This could include a more detailed definition of terms used in the quantity, such as the entry for “sea ice extent loss in March,” which defines the term “sea ice extent,” or useful historical information about the measurement. In our example of atmospheric CO_2_ concentration, the summary explains that the measurement is made at the Mauna Loa observatory and points out the seasonal oscillations that are observed. The following “Method” field describes the method by which the quantity was measured, inferred, or estimated (Figure 1G). This field also provides an assessment of the uncertainty in the value, which may include a description of how confidence intervals were computed or a list of critical assumptions that were made to estimate missing data.

All fields through “Method” (Figures 1A–1G) depend on manual curation and interpretation by database administrators. The following two fields, “Source” and “Dataset” (Figures 1H and 1I), provide direct links to the primary source reference and the relevant data. Both of these fields are direct links (shown as insets in Figure 1). The “Source” field can point to either the published scientific literature or the resource page of a governmental, industrial, or non-governmental organization data deposition URL. The “Dataset” field links directly to either a CSV format of the data or to a folder with global and regional values within the corresponding GitHub repository. As discussed in Note S1, the vast majority of these data files have been converted into a “tidy-data” format^38^ by database administrators, which maximizes programmatic readability.

When possible, a graphical time series of the data is also presented as an interactive plot (Figure 1J). These plots enable users to quickly apprehend time-dependent trends in the data without downloading or processing the dataset. The data sources we rely on in building the database are remarkably varied, coming from governmental, industrial, and primary scientific sources, each with their own specific data use protection policies. Each entry (Figure 1K) also provides a link to the data use policy for each individual dataset. While not available for every entry, the majority of quantities we have curated in the Human Impacts Database contain measurements over time. The last field gives the username of the administrator who generated this entry (Figure 1L). Their affiliation and contact information are available on the database’s “About” page. We invite the reader to contact the administrators collectively—through our “Contact” page or directly through our personal emails as provided on the “About” page—with questions, concerns, or suggestions.

While Figure 1 is a representative example, each quantity in the Human Impacts Database tells a different story. Easy and centralized access to different entries allows users to learn about the magnitude of human impacts and also study the interactions between different human activities, which, as we discuss in the next section, are deeply intertwined.

### Global magnitudes

**Figure 2:**
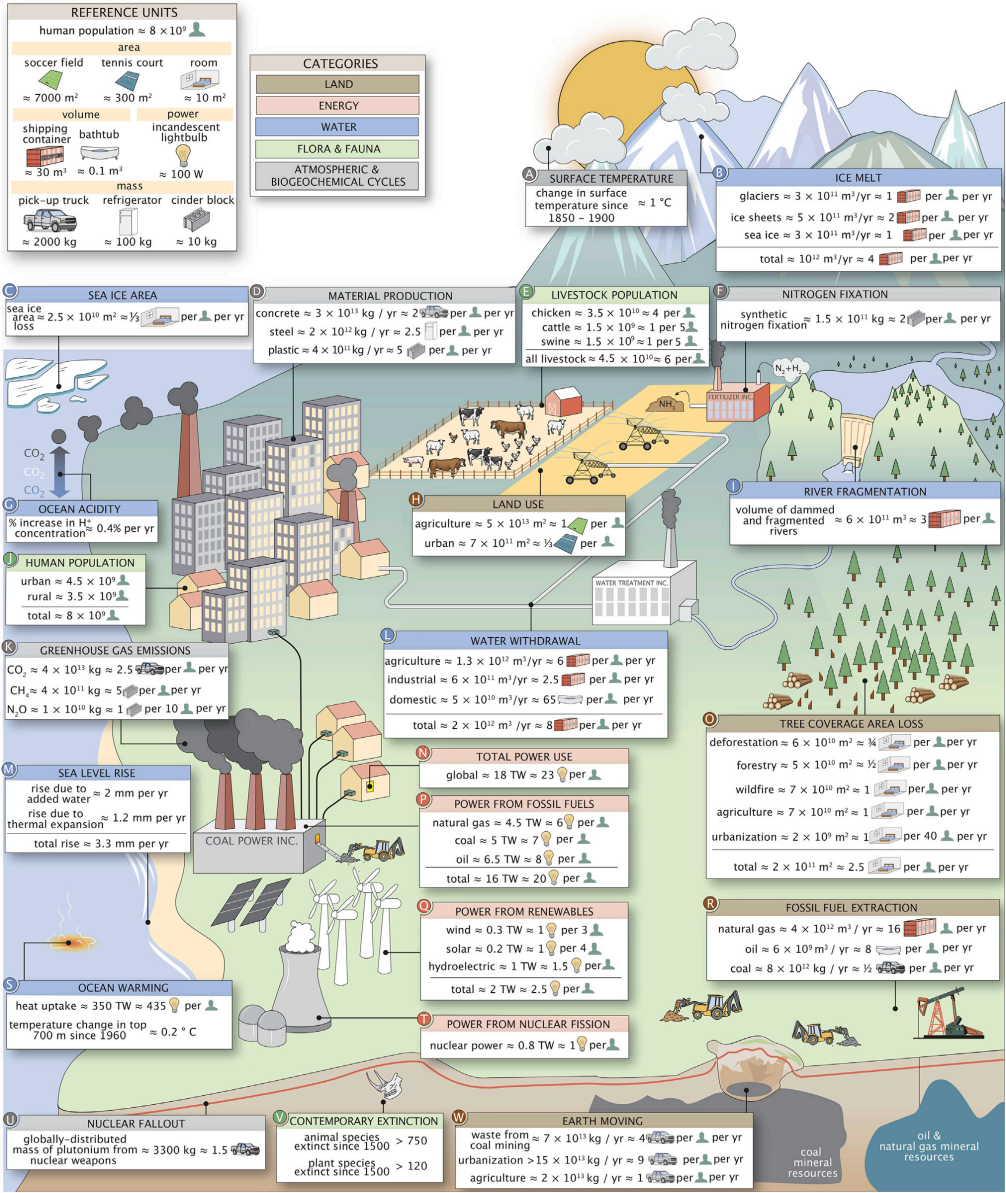
Human impacts on the planet and their relevant magnitudes Relative units and the broad organizational categories are shown in the top left. Source information and contextual comments for each subpanel are presented in Note S2.

In Figure 2, we provide an array of quantities that we believe to be key in developing a “feeling for the numbers” associated with human impacts on the Earth system. All of the quantities in Figure 2 are drawn from entries in the database and grouped into the same categories used in the database: land, water, flora and fauna, atmosphere and biogeochemical cycles, and energy (see color scheme at the top of Figure 2). Although the impacts considered here necessarily constitute an incomplete description of human interaction with the planet, these numbers encompass many that are critically important, such as the volume of liquid water resulting from ice melt (Figure 2B), the extent of urban and agricultural land use (Figure 2H), global power consumption (Figure 2N), and the heat uptake and subsequent warming of the ocean surface (Figure 2S). In many cases, the raw numbers are astoundingly large and can therefore be difficult to fathom. Rather than reporting only bare “scientific” units, we present each quantity (when possible) in units that are intended to be relatable as “per capita” values to a broad audience who are members of (or familiar with) typical Western lifestyles. Consider, for example, the 18 TW global power consumption (Figure 2N). For most audiences, it can be difficult to conceptualize what a watt is, let alone the sheer magnitude of a *terawatt*. However, most prospective users of this database likely have a familiarity with the warmth of a 100 W light bulb. With this in mind, we can do a simple conversion to say that the global average power use per person is comparable to constantly running ≈ 23 light bulbs per person, making the impact a bit more tangible.

Exploring these numbers reveals a number of intriguing quantities and relationships. For example, agriculture repeatedly appears as a major contributor to many human impacts, dominating both global land (Figure 2H) and global water use (Figure 2L) and accounting for approximately a third of global tree cover area loss (Figure 2O). In addition, an enormous mass of nitrogen is synthetically fixed through the Haber-Bosch process, primarilyto produce fertilizer (Figure 2F), which is a major cause of emissions of N_2_O (Figure 2K), which is a potent greenhouse gas. About 45 billion livestock are raised on agricultural lands (Figure 2E), which, together with rice paddies, produce a majority of anthropogenic methane emissions (the greenhouse gas CH_4_; Figure 2K). On the other hand, urban land area accounts for a very small fraction of land area use (≈ 1%, Figure 2H), and the expansion of cities and suburbs accounts for only ≈ 1% of global tree cover area loss (Figure 2O). This is not to say, however, that urban centers are negligible in their global impacts. As urban and suburban areas currently house more than half of the global human population (Figure 2J), many human impacts are linked to industries that directly or indirectly support urban populations’ demand for food, housing, travel, electronics, and other goods. For example, the pursuit of urbanization is the dominating factor in the mass of earth moved on an annual basis (Figure 2W).

Collectively, the ≈ 8 billion humans on Earth (Figure 2J) consume nearly 20 TW of power, equivalent to 23 100 W light bulbs per person (Figure 2N). Around 80% of this energy derives from the combustion of fossil fuels (Figure 2P). This results in a tremendous mass of CO_2_ being emitted annually (Figure 2K), of which only ≈ 50% remains in the atmosphere (HuID 70632). A sizable portion of the emissions are absorbed by the oceans (HuID 99089), leading to a steady increase in ocean acidity (Figure 2G) and posing risks to marine ecosystems.^39^ Furthermore, increasing average global temperatures, primarily caused by greenhouse gas emissions, contribute to sea-level rise not only in the form of added water from melting ice (Figure 2B and 2M), but also due to thermal expansion of ocean water (Figure 2M), which accounts for ≈ 30% of observed sea-level rise.^40^ These are just a few ways in which one can traverse the impacts illustrated in Figure 2, revealing the remarkable extent to which these impacts are interconnected. We encourage the reader to explore this figure in a similar manner, blazing their own trail through the values.

### Regional distribution

**Figure 3:**
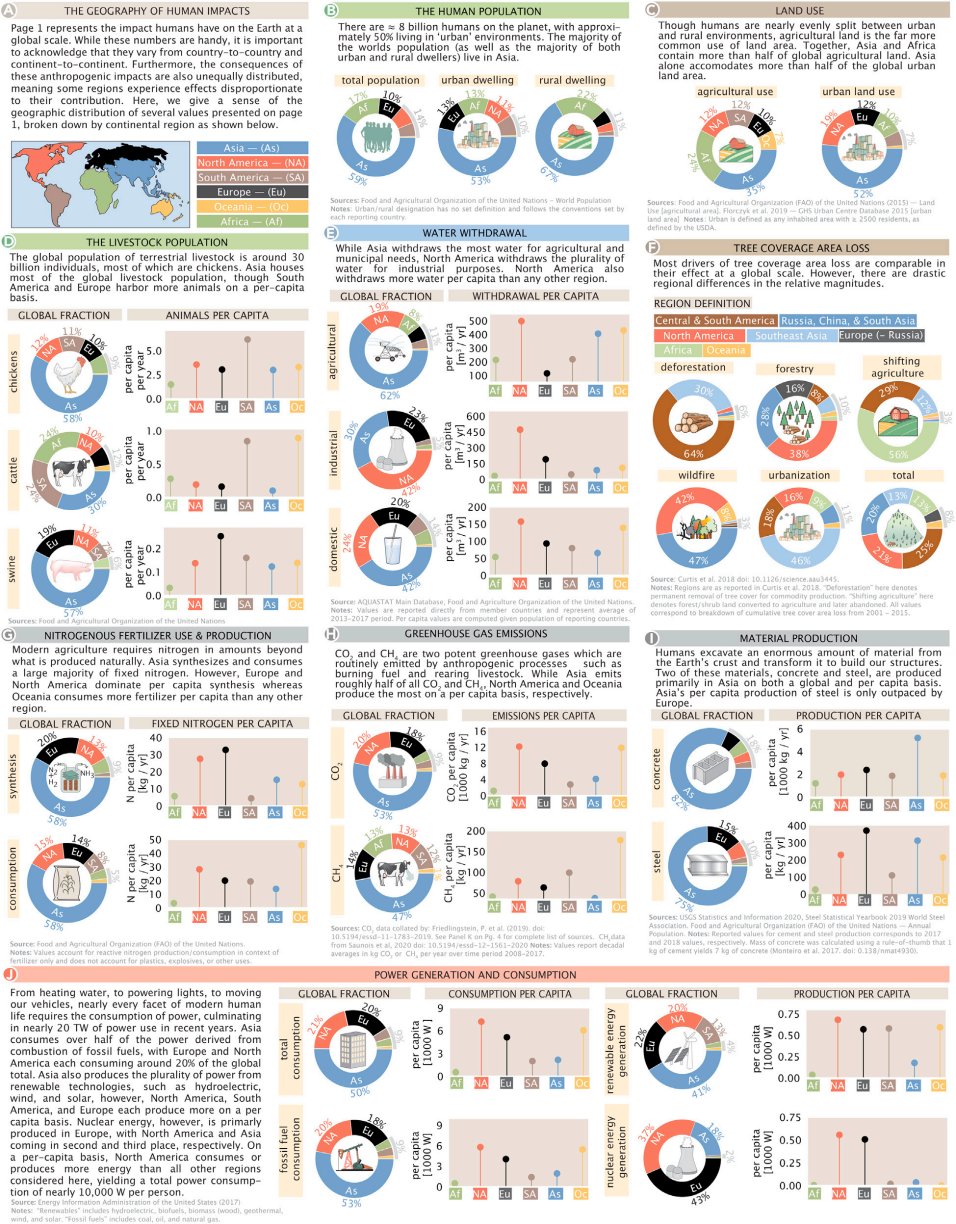
Regional distribution of anthropogenic effects (A) Several quantities from Figure 2 were selected, and the relative magnitudes were broken down by subcontinental area. (B–J) Donut charts in all sections show the relative contributions of each quantity by region. Ball-and-stick plots show the per-capita breakdown of each quantity across geographic regions. All data for global and per-capita breakdowns correspond to the latest year for which data were available. The regional breakdown for deforestation uses the regional convention as reported in the source data.^41^

While Figure 2 presents the magnitude of human impacts at a global scale, it is important to recognize that these impacts—both their origins and their repercussions—are variable across the globe. That is, different societies vary in their preferences for food (e.g., Americans consume relatively little fish) and modes of living (e.g., apartments versus houses), have different levels of economic development (e.g., Canada compared with Malaysia), rely on different natural resources to build infrastructure (e.g., wood versus concrete) and generate power (e.g., nuclear versus coal), and promote different extractive or polluting industries (e.g., lithium mining versus palm oil farming). Some of these regional differences are evident in Figure 3, which summarizes regional breakdowns of several drivers of global human impacts, e.g., livestock populations and greenhouse gas emissions.

Just as impactful human activities like coal power generation and swine farming are more common in some regions than others (Figure 2), the impacts of human activities affect some regions more than others.^42^ Figure 3 displays a coarse regional breakdown of the numbers from Figure 2 for which regional distributions could be determined from the literature. The region definitions used in Figure 3 are similar to the definitions set forth by the Food and Agricultural Organization (FAO) of the United Nations, assigning the semi-continental regions of North America, South America, Africa, Europe (including Russia), Asia, and Oceania. Here, we specify both the total contribution of each region and the per-capita value, given the population of that region as of the year(s) in which the quantity was measured.

Much as in the case of Figure 2, interesting details emerge from Figure 3. For example, Asia dominates global agricultural water withdrawal (excluding natural watering via rainfall), using about 62% of the total, while North America takes the lead in industrial water withdrawal. Interestingly, on a per-capita basis, North America withdraws the most water for all uses: agricultural, industrial, and domestic.

North America also emits more CO_2_ per capita than any other region, with Oceania and Europe coming second and third, respectively. This disparity can be partially understood by considering the regional distribution of fossil fuel consumption, the dominant source of CO_2_ emissions (Figure 3J). While Asia consumes more than half of the total fossil fuel energy, per-capita consumption is markedly lower than in North America, Europe, and Oceania (Figure 3J). Interestingly, the story is different when it comes to methane. Oceania and South America are the largest emitters of anthropogenic CH_4_, mainly due to a standing population of cattle that rivals that of humans in those regions (Figure 3D) and produces this potent greenhouse gas through enteric fermentation.^33^ Regional disparities are also apparent in the means of energy production. While consuming only 4% of the total power, South America generates about 14% of the renewable energy. Nuclear power generation, on the other hand, is dominated by North America and Europe, while Oceania, which has a single research-grade nuclear reactor, generates nearly zero nuclear energy.

Investigating the causes of forest loss by geographic region likewise highlights interesting differences. At a global level, all drivers of forest loss are comparable in magnitude, except for urbanization, which accounts for ≈ 1% of total annual tree cover area loss (Figure 2O). Despite comparable magnitudes, different drivers of forest loss have different long-term consequences.^30^ Forest loss due to wildfires and forestry often result in regrowth, while commodity-driven harvesting and urbanization tend to be drivers of long-lasting deforestation.^43^^,^^44^ Central and South America account for about 65% of commodity-driven deforestation (meaning clear-cutting and human-induced fires with no substantial regrowth of tree cover), whereas a majority of forest loss due to shifting agriculture occurs in Africa (where regrowth does occur). Together, wildfires in North America, Russia, China, and South Asia make up nearly 90% of losses due to fire.^41^ While urbanization is the smallest driver of tree cover loss globally, it can still have strong impacts at the regional level, perturbing local ecosystems and biodiversity.^45^^,^^46^

### Time series

**Figure 4:**
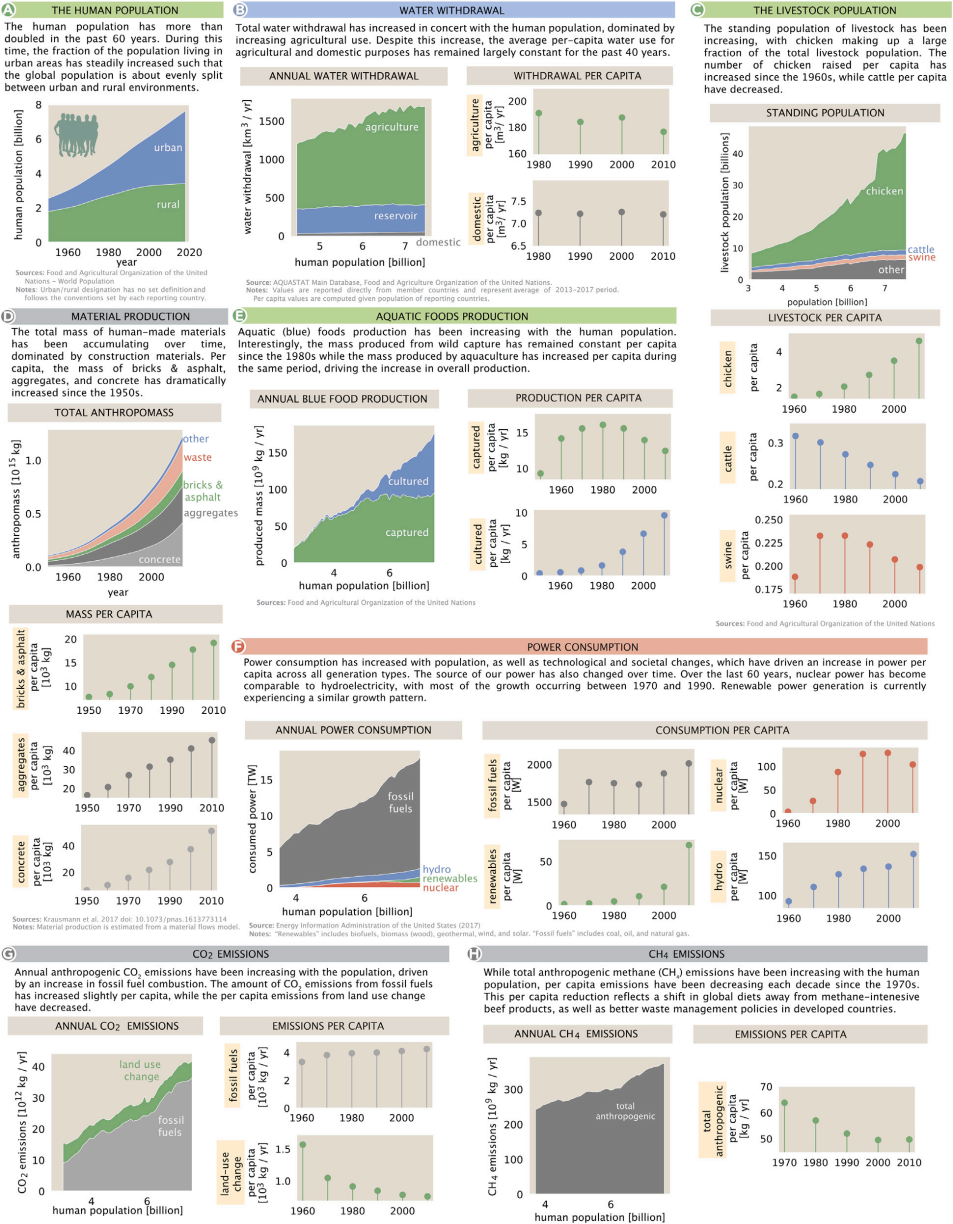
Temporal dynamics of key human impacts (A) Several quantities from Figure 2 were selected, and the magnitudes were plotted as a function of either time (for cumulative quantities such as anthropomass) or human population. (B–H) Ball-and-stick plots show the per-capita breakdown as decadal averages to give a more reflective view of cultural and technological shifts than year-to-year variation.

When available, the Human Impacts Database includes time-series data for each quantity. Just as the regional distributions of impactful human activities help us understand differences between societies and regions, studying the history of these activities highlights recent technological and economic developments that intensify or reduce their impacts. When considering the history of human impacts on the Earth, it is natural to start by considering the growth of the human population over time. As shown in Figure 4, the global human population grew nearly continually over the past 80 years, with the current population nearing 8 billion. Historically, most of the global human population lived in rural areas (about 70% as of 1950, HuID 93995). Recent decades have been marked by a substantial shift in how humans live globally, with around half of the human population now living in urban or suburban settings (≈ 55%, HuID 93995).

Given the growth of the human population, it is reasonable to consider that human population may be the most natural scale to measure human impacts.^47^ To assess this possibility, we plotted per-capita impacts over several decades (Figure 4). If impacts are growing in direct proportion to the human population, per-capita impacts would be constant over time. Indeed, this is roughly true for per-capita water withdrawals over the past 40 years (Figure 4B). Deviations from proportionality may indicate important changes in human activities. For example, in recent decades, per-capita chicken populations grew by nearly 2-fold, while per-capita cattle populations shrunk by roughly 25%, reflecting a modest transition away from beef and toward chicken as a source of animal protein in global diets (HuIDs 40696 and 79776).

One very visible impact accompanying the shift of the human population to urban environments is the increase in production of anthropogenic mass: materials such as concrete, steel, lumber, and plastics used to build roads, buildings, machines, packaging, and other useful human-made items. Since these materials are degraded very slowly, anthropogenic mass has been accumulating over time. In addition, the mass of concrete, aggregates like asphalt, and bricks per capita has been increasing since the 1950s (Figure 4D). Concrete, in particular, has increased from less than 10 tons per person in the 1950s to almost 30 tons per person in the 2010s. This increase in per-capita anthropogenic mass means that the increase in production of these materials is outpacing the growth of the human population.

These material production trends have been enabled, in part, by a sustained increase in power generation. As evident from Figure 4, total power consumption has been increasing roughly proportionally with the human population. Per-capita consumption has also increased across all generation types, including fossil fuels, hydropower, nuclear, and renewables. The growth among nuclear and renewables has been especially dramatic, and nuclear power now roughly equals hydropower production. Production of crops, aquaculture, and populations of livestock are all likewise correlated with growth in the human population (Figures 4C and 4E). The total number of livestock has increased with the human population, primarily due to increasing chicken populations as discussed above. The dominant means of aquatic food production has also shifted over this time: until roughly 1980, nearly all seafood was captured wild, but since then aquaculture has grown to account for roughly ½ of aquatic food production (HuID 61233, Figure 4E).

Turning our focus to greenhouse gases, we see that annual anthropogenic CO_2_ emissions have been increasing with the population (Figure 4G). Burning of fossil fuels is the dominant contributor to anthropogenic emissions and has increased slightly on a per-capita basis over the past 60 years. In contrast, as the pace of global deforestation has slowed,^48^^,^^49^ emissions of CO_2_ due to land-use change have decreased per capita. These two trends roughly neutralize each other, leading to little overall change in CO_2_ emissions per capita since the 1960s. Akin to CO_2_ emissions due to land-use change, CH_4_ emissions show a sublinear trend with human population, partially due to a decline in ruminant livestock per capita (Figures 4C and 4H).

## Discussion

Quantitative literacy is necessary for “understanding” in nearly all branches of science. As our collective knowledge of anthropogenic impacts expands, it has become challenging to sift through the literature to collect specific numbers useful for both calculation and communication. We have attempted to reduce this barrier to entry on several fronts. We have canvassed the scientific literature, governmental, industrial, and international reports to assemble a broad, quantitative picture of how human activities have affected the Earth's atmosphere, oceans, rivers, lands, biota, chemistry, and geology. In doing so, we have created an online, searchable database housing an array of quantities and data that describe different facets of the human-Earth interface. We view this database as an accessory, rather than a replacement, for the myriad scientific databases that exist and are publicly available on the internet (some of which are listed on the database website www.anthroponumbers.org/catalog/databases). While these databases are invaluable resources for accessing scientific data, the Human Impacts Database is built from the ground up with the intention of being broadly accessible to scientists and the curious general public alike to help build the collective quantitative literacy of the Anthropocene. Beyond the database, we have assembled these data into a comprehensive snapshot, released alongside this writing as a standalone graphical document (Data S1), with all underlying data, associated uncertainties, and referencing housed in the Human Impacts Database. While necessarily incomplete, these resources provide a broad view of the ways in which human activities are having an impact on the Earth on multiple fronts.

One insight that emerges from a holistic consideration of these diverse human activities together is that they are deeply intertwined and driven by a small number of pivotal factors: the size of the human population, the composition of our diets, and our demand for materials and energy to build and power our increasingly complex and mechanized societies. Understanding the scale of human agriculture and water and power usage provides a framework for understanding most of the numerical gallery presented in Figure 2. Perhaps unsurprisingly, we find that feeding the growing human population is a major driver of a large swath of human impacts on Earth, dominating global land (Figure 2H, HuID 29582) and water use (Figure 2L; HuIDs 84545, 43593, 95345), as well as significantly contributing to tree cover loss (Figure 2O, HuID 24388), earth moving (Figure 2W; HuIDs 19415, 41496), and anthropogenic nitrogen fixation (Figure 2F; HuIDs 60580, 61614), to name a few such examples. The Human Impacts Database provides a resource to explore relationships between values temporally, globally, and locally, and go beyond the standalone values often reported in isolation or cast solely through the lenses of impact, population, affluence, and technology (I = PAT) relationships.

It is common in this setting to argue that the bewildering breadth and scale of human impacts should motivate some specific remediation at the global or local scale. We, instead, take a more modest "just the facts" approach. The numbers presented here show that human activities affect our planet to a large degree in many different and incommensurate ways, but they do not provide a roadmap for the future. Rather, we contend that any plans for the future should be made in the light of a comprehensive and quantitative understanding of the interconnected ways in which human activities impact the Earth system globally (Figure 2), locally (Figure 3), and temporally (Figure 4). Achieving such an understanding will require the synthesis of a broad literature across many disciplines. While the quantities we have chosen to explore are certainly not exhaustive, they represent some of the key axes that frequently drive scientific and public discourse and shape policy across the globe.

Earth is the only habitable planet we know of, so it is crucial to understand how we got here and where we are going. That is, how (and why) have human impacts changed over time? How are they expected to change in the future? For every aspect of human entanglement with the Earth system—from water use to land use, greenhouse gas emissions, mining of precious minerals, and so on—there are excellent studies measuring impacts and predicting their future trajectories. Of particular note are the data-rich and explanatory reports from the Intergovernmental Panel on Climate Change^50^^,^^51^ and the efforts toward defining “planetary boundaries.”^52^ We hope that the Human Impacts Database and the associated resources with this work provide a reference to explore the human-induced interdependencies between many axes of the human-Earth system and will engage the scientific community, ultimately helping humanity coexist stably with the only planet we have.

## Experimental procedures

### Resource availability

#### Lead contact

Requests for further information should be directed to and will be fulfilled by the lead contact, Griffin Chure (griffinchure@gmail.com).

#### Materials availability

No materials were used in the generation of this work, other than the code and data as described below. We have collated all data shown in Figure 2, Figure 3, Figure 4, along with all information in Note S2 as a printable, “graphical snapshot” (Data S1).

#### Data and code availability

For every dataset included in the database, there is a folder in the GitHub repository https://github.com/rpgroup-pboc/human_impacts (DOI: 10.5281/zenodo.4453276) that includes the source data, the processed data, and the code to generate the “tidy” data from the source data. Each folder also includes a README file that includes information about the dataset. In addition, all of the code used to generate the figures can be found in the GitHub repository under the “figures” folder. We strongly encourage the scientific community to fork this repository, submit pull requests, and open new constructive issues through the GitHub repository interface.

### The database and the FAIR principles of data reuse

The primary goal of the Human Impacts Database is to provide a resource for the rapid discovery quantities related to the human-Earth system while minimizing the grunt work needed to access (and understand) the underlying data. This means that facilitating data reuse and reproducibility of any analyses is paramount to the importance of the database. To that end, we abide by the *FAIR Guiding Principles for Scientific Data Management and Stewardship* (www.go-fair.org/fair-principles/). These principles are guidelines to maximize the findability, accessibility, interoperability, and reusability of original scientific data. The database closely follows these principles, as is briefly outlined below:

• Findability: The underlying data can be easily searched and navigated, permitting rapid discovery. Individual entries are assigned a unique integer identifier that serves as a permanent referencing tool and are provided with rich metadata about the method of determination, original source, data use protection policy, and quantitative value in diverse units.
• Accessibility: The original source of the underlying data is always reported hyperlinked when legally permissible. The transformation, collation, or manipulation of the underlying data that was necessary to add it to the Human Impacts Database is preserved under a publicly accessible, version-controlled, GitHub repository (github.com/rpgroup-pboc/human_impacts) and is permanently accessible via https://doi.org/10.5281/zenodo.4453276. This protects against permanent loss of the data even if an entry is deleted from the database.
• Interoperability: The data are provided in a human readable format with an emphasis on description of the data and their source. The vast majority of datasets are transformed programmatically to follow a “tidy,” long-form format that facilitates computational analysis of the data. As the values are hand curated and the target audience is a curious human, we have not developed an API for programmatic access of the database, and do not have plans to do so in the foreseeable future.
• Reusability: All entries in the database and the corresponding GitHub repository are extensively annotated with rich metadata, preventing the need for guesswork as to how the data were collected or what the column names refer to in the original or processed data. Furthermore, all data held in the database and repository follow the legal guidelines as presented by their original owner. This licensing is directly linked to in each entry.

## Web resources

BioNumbers Database, https://bionumbers.hms.harvard.edu
Human Impacts Database, http://anthroponumbers.org

## Supplemental information

Document S1. Notes S1 and S2

Data S1. Graphical Snapshot

Document S3. Article plus supplemental information

## References

[1] Fischer W.W., Hemp J., Johnson J.E. (2016). Evolution of oxygenic photosynthesis. Annu. Rev. Earth Planet Sci..

[2] Hodgskiss M.S.W., Crockford P.W., Peng Y., Wing B.A., Horner T.J. (2019). A productivity collapse to end Earth’s Great Oxidation. Proc. Natl. Acad. Sci. USA.

[3] Gumsley A.P., Chamberlain K.R., Bleeker W., Söderlund U., de Kock M.O., Larsson E.R., Bekker A. (2017). Timing and tempo of the great oxidation event. Proc. Natl. Acad. Sci. USA.

[4] Sessions A.L., Doughty D.M., Welander P.V., Summons R.E., Newman D.K. (2009). The continuing puzzle of the great oxidation event. Curr. Biol..

[5] Estes J.A., Terborgh J., Brashares J.S., Power M.E., Berger J., Bond W.J., Carpenter S.R., Essington T.E., Holt R.D., Jackson J.B.C. (2011). Trophic downgrading of planet earth. Science.

[6] Springer A.M., Estes J.A., van Vliet G.B., Williams T.M., Doak D.F., Danner E.M., Forney K.A., Pfister B. (2003). Sequential megafaunal collapse in the North Pacific Ocean: an ongoing legacy of industrial whaling?. Proc. Natl. Acad. Sci. USA.

[7] Hale S.L., Koprowski J.L. (2018). Ecosystem-level effects of keystone species reintroduction: a literature review: effects of keystone species reintroduction. Restor. Ecol..

[8] Holdo R.M., Sinclair A.R.E., Dobson A.P., Metzger K.L., Bolker B.M., Ritchie M.E. (2009). A disease-mediated trophic cascade in the Serengeti and its implications for ecosystem. PLoS Biol..

[9] Ceballos G., Ehrlich P.R., Barnosky A.D., García A., Pringle R.M., Palmer T.M. (2015). Accelerated modern human–induced species losses: entering the sixth mass extinction. Sci. Adv..

[10] Davidson A.D., Hamilton M.J., Boyer A.G., Brown J.H., Ceballos G. (2009). Multiple ecological pathways to extinction in mammals. Proc. Natl. Acad. Sci. USA.

[11] Fortin D., Beyer H.L., Boyce M.S., Smith D.W., Duchesne T., Mao J.S. (2005). Wolves influence Elk movements: Behavior shapes a trophic cascade in Yellowstone National Park. Ecology.

[12] Tronstad L.M., Hall R.O., Koel T.M., Gerow K.G. (2010). Introduced lake trout produced a four-level trophic cascade in Yellowstone Lake. Trans. Am. Fish. Soc..

[13] Palumbi S.R. (2001). Humans as the world’s greatest evolutionary force. Science.

[14] Balmaseda M.A., Trenberth K.E., Källén E. (2013). Distinctive climate signals in reanalysis of global ocean heat content. Geophys. Res. Lett..

[15] Cheng L., Trenberth K.E., Fasullo J., Boyer T., Abraham J., Zhu J. (2017). Improved estimates of ocean heat content from 1960 to 2015. Sci. Adv..

[16] Bar-On Y.M., Phillips R., Milo R. (2018). The biomass distribution on Earth. Proc. Natl. Acad. Sci. USA.

[17] Elhacham E., Ben-Uri L., Grozovski J., Bar-On Y.M., Milo R. (2020). Global human-made mass exceeds all living biomass. Nature.

[18] IPCC (2019).

[19] Peng H., Ke C., Shen X., Li M., Shao Z. (2020). Summer albedo variations in the Arctic Sea ice region from 1982 to 2015. Int. J. Climatol..

[20] Marcianesi F., Aulicino G., Wadhams P. (2021). Arctic sea ice and snow cover albedo variability and trends during the last three decades. Polar Sci.

[21] Mouginot J., Rignot E., Bjørk A.A., van den Broeke M., Millan R., Morlighem M., Noël B., Scheuchl B., Wood M. (2019). Forty-six years of Greenland Ice Sheet mass balance from 1972 to 2018. Proc. Natl. Acad. Sci. USA.

[22] MacGregor J.A., Fahnestock M.A., Catania G.A., Aschwanden A., Clow G.D., Colgan W.T., Gogineni S.P., Morlighem M., Nowicki S.M.J., Paden J.D. (2016). A synthesis of the basal thermal state of the Greenland Ice Sheet. J. Geophys. Res. Earth Surf..

[23] Kwok R. (2018). Arctic sea ice thickness, volume, and multiyear ice coverage: losses and coupled variability (1958–2018). Environ. Res. Lett..

[24] Massom R.A., Scambos T.A., Bennetts L.G., Reid P., Squire V.A., Stammerjohn S.E. (2018). Antarctic ice shelf disintegration triggered by sea ice loss and ocean swell. Nature.

[25] Notz D., Stroeve J. (2016). Observed Arctic sea-ice loss directly follows anthropogenic CO_2_ emission. Science.

[26] Stroeve J., Notz D. (2018). Changing state of Arctic sea ice across all seasons. Environ. Res. Lett..

[27] Goode P.R., Pallé E., Shoumko A., Shoumko S., Montañes-Rodriguez P., Koonin S.E. (2021). Earth’s albedo 1998–2017 as measured from earthshine. Geophys. Res. Lett..

[28] Friedlingstein P., Jones M.W., O'sullivan M., Andrew R.M., Hauck J., Peters G.P., Peters W., Pongratz J., Sitch S., Le Quéré C. (2019). Global carbon budget 2019. Earth Syst. Sci. Data.

[29] Houghton R.A., Nassikas A.A. (2017). Global and regional fluxes of carbon from land use and land cover change 1850-2015: Carbon emissions from land use. Glob. Biogeochem. Cycles.

[30] Hansis E., Davis S.J., Pongratz J. (2015). Relevance of methodological choices for accounting of land use change carbon fluxes. Glob. Biogeochem. Cycles.

[31] Tian H., Xu R., Canadell J.G., Thompson R.L., Winiwarter W., Suntharalingam P., Davidson E.A., Ciais P., Jackson R.B., Janssens-Maenhout G. (2020). A comprehensive quantification of global nitrous oxide sources and sinks. Nature.

[32] Keeling C.D. (1960). The concentration and isotopic abundances of carbon dioxide in the atmosphere. Tellus.

[33] Saunois M., Stavert A.R., Poulter B., Bousquet P., Canadell J.G., Jackson R.B., Raymond P.A., Dlugokencky E.J., Houweling S., Patra P.K. (2020). The global methane budget 2000–2017. Earth Syst. Sci. Data.

[34] Yoon J.-H., Kravitz B., Rasch P.J., Simon Wang S.Y., Gillies R.R., Hipps L. (2015). Extreme fire season in California: A glimpse into the future?. Bull. Am. Meteorol. Soc..

[35] Seager R., Hoerling M., Schubert S., Wang H., Lyon B., Kumar A., Nakamura J., Henderson N. (2015). Causes of the 2011–14 California drought. J. Clim..

[36] Milo R., Jorgensen P., Moran U., Weber G., Springer M. (2010). BioNumbers—the database of key numbers in molecular and cell biology. Nucleic Acids Res..

[37] Milo R., Phillips R. (2016).

[38] Wickham H. (2014). Tidy data. J. Stat. Softw..

[39] Andersson A., Kline D., Edmunds P., Archer S., Bednaršek N., Carpenter R., Chadsey M., Goldstein P., Grottoli A., Hurst T. (2015). Understanding ocean acidification impacts on organismal to ecological scales. Oceanography.

[40] Frederikse T., Landerer F., Caron L., Adhikari S., Parkes D., Humphrey V.W., Dangendorf S., Hogarth P., Zanna L., Cheng L., Wu Y.H. (2020). The causes of sea-level rise since 1900. Nature.

[41] Curtis P.G., Slay C.M., Harris N.L., Tyukavina A., Hansen M.C. (2018). Classifying drivers of global forest loss. Science.

[42] Patz J.A., Campbell-Lendrum D., Holloway T., Foley J.A. (2005). Impact of regional climate change on human health. Nature.

[43] Bowman D.M.J.S., Balch J.K., Artaxo P., Bond W.J., Carlson J.M., Cochrane M.A., D’Antonio C.M., DeFries R.S., Doyle J.C., Harrison S.P. (2009). Fire in the earth system. Science.

[44] Santín C., Doerr S.H., Preston C.M., González-Rodríguez G. (2015). Pyrogenic organic matter production from wildfires: A missing sink in the global carbon cycle. Glob. Change Biol..

[45] Olivier T., Thébault E., Elias M., Fontaine B., Fontaine C. (2020). Urbanization and agricultural intensification destabilize animal communities differently than diversity loss. Nat. Commun..

[46] Newbold T., Hudson L.N., Hill S.L.L., Contu S., Lysenko I., Senior R.A., Börger L., Bennett D.J., Choimes A., Collen B. (2015). Global effects of land use on local terrestrial biodiversity. Nature.

[47] Syvitski J., Waters C.N., Day J., Milliman J.D., Summerhayes C., Steffen W., Zalasiewicz J., Cearreta A., Gałuszka A., Hajdas I. (2020). Extraordinary human energy consumption and resultant geological impacts beginning around 1950 CE initiated the proposed Anthropocene Epoch. Commun. Earth Environ..

[48] FAO (2020).

[49] Minx J.C., Lamb W.F., Andrew R.M., Canadell J.G., Crippa M., Döbbeling N., Forster P.M., Guizzardi D., Olivier J., Peters G.P. (2021). A comprehensive and synthetic dataset for global, regional, and national greenhouse gas emissions by sector 1970–2018 with an extension to 2019. Earth Syst. Sci. Data.

[50] Masson-Dellmotte V., Zhai P., Pörtner H.O., Roberts D., Skea J., Shukla P.R. (2018). Special Report: Global Warming of 1.5 oC.

[51] Pörtner, H.-O., Roberts, D.C., Masson-Delmotte, V., and Zhai, P., eds. (2019). IPCC Special Report on the Ocean and Cryosphere in a Changing Climate (IPCC).

[52] Steffen W., Richardson K., Rockström J., Cornell S.E., Fetzer I., Bennett E.M., Biggs R., Carpenter S.R., de Vries W., de Wit C.A. (2015). Sustainability. Planetary boundaries: guiding human development on a changing planet. Science.

