# Supplementary material for: Anthroponumbers.org: A quantitative database of human impacts on Planet Earth
Source: Patterns (N Y). 2022 Aug 3;3(9):100552. doi: 10.1016/j.patter.2022.100552 (PMC9481956; doi:10.1016/j.patter.2022.100552)
Supplement: Data S1. Graphical Snapshot [file mmc2.pdf]

THE ANTHROPOCENE
by the numbers

Griffin Chure[1,2,†,*], Rachel A. Banks[3,4,5,†], Avi I. Flamholz[3,4], Nicholas S. Sarai[6], Mason Kamb[5], Ignacio Lopez-Gomez[4,7], Yinon M. Bar-On[8], Ron Milo[8], Rob Phillips[3,5,9,*]

1. Department of Biology, Stanford University, Palo Alto, CA, USA.
California Institute of Technology, Pasadena, CA, USA, 91125:
2. Department of Applied Physics
3. Division of Biology and Biological Engineering
4. Resnick Institute of Sustainability
6. Division of Chemistry and Chemical Engineering
7. Department of Environmental Science and Engineering;
9. Department of Physics
5. Chan-Zuckerberg BioHub, San Francisco, CA, 94158
8. Weizmann Institute of Science, Rehovot 7610001, Israel: Department of Plant and Environmental Sciences
†. contributed equally *. Address correspondence to griffinchure@gmail.com and phillips@pboc.caltech.edu

ABSTRACT

A candidate for the greatest experiment of the last 10,000 years is the presence and action of modern human beings on planet Earth, the often complex results of which are now being felt on various fronts. While there has been a deluge of careful studies exploring each facet of these "human impacts" on Earth, they are often highly focused and necessarily technical, rarely displaying their integration with other human impacts as a whole. In this snapshot, we present a diverse (yet necessarily incomplete) array of quantities that summarize the broad reach of human action across the planet.

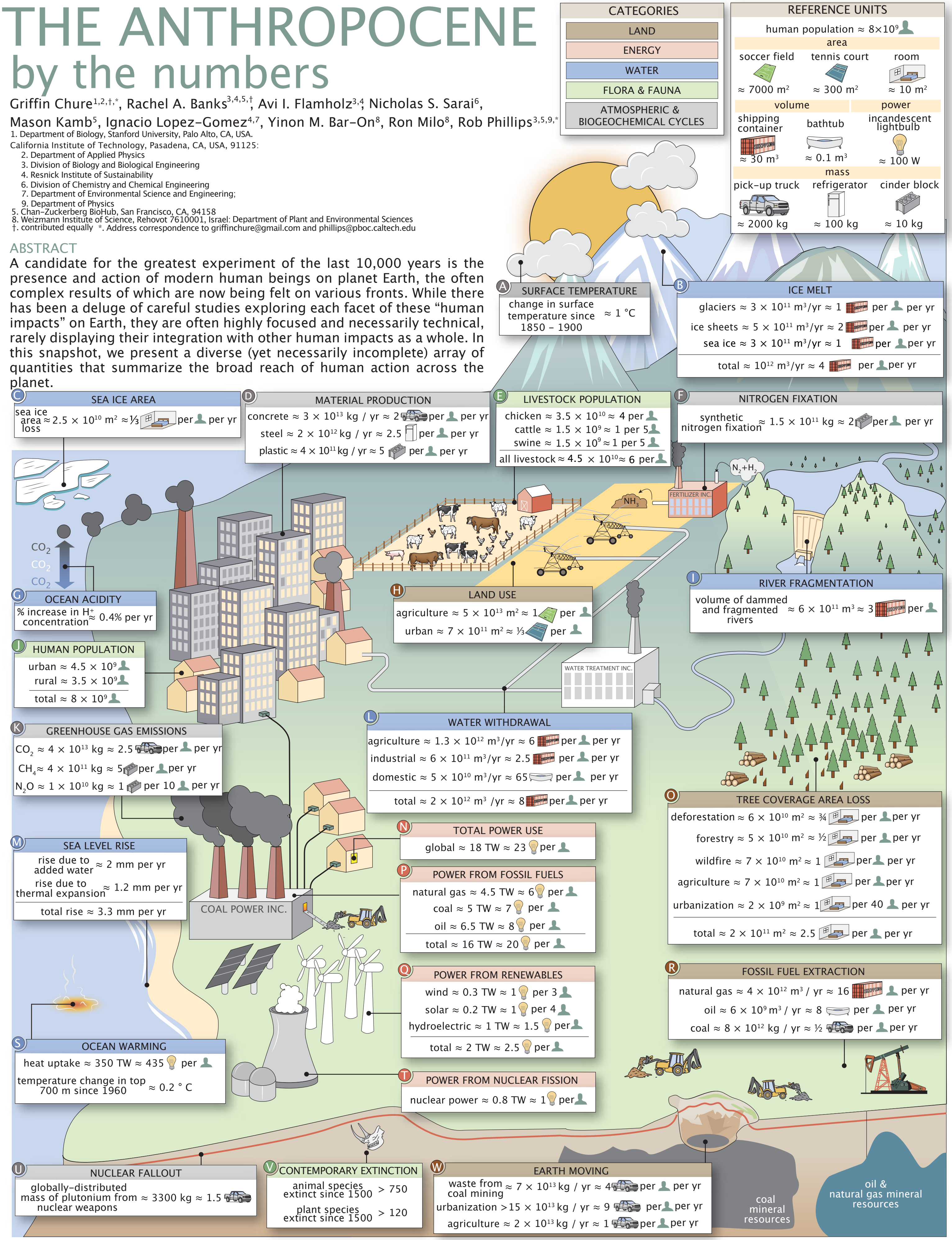

The Anthropocene by the Numbers — Impacts By Region

A THE GEOGRAPHY OF HUMAN IMPACTS

Page 1 represents the impact humans have on the Earth at a global scale. While these numbers are handy, it is important to acknowledge that they vary from country–to–country and continent–to–continent. Furthermore, the consequences of these anthropogenic impacts are also unequally distributed, meaning some regions experience effects disproportionate to their contribution. Here, we give a sense of the geographic distribution of several values presented on page 1, broken down by continental region as shown below.

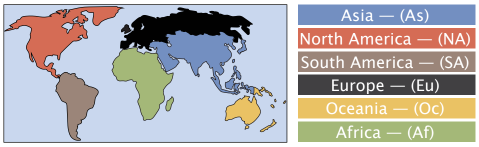

B THE HUMAN POPULATION

There are ≈ 8 billion humans on the planet, with approximately 50% living in 'urban' environments. The majority of the worlds population (as well as the majority of both urban and rural dwellers) live in Asia.

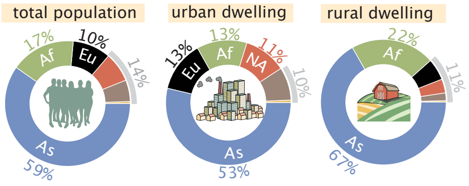

Sources: Food and Agricultural Organization of the United Nations – World Population
Notes: Urban/rural designation has no set definition and follows the conventions set by each reporting country.

C LAND USE

Though humans are nearly evenly split between urban and rural environments, agricultural land is the far more common use of land area. Together, Asia and Africa contain more than half of global agricultural land. Asia alone accomodates more than half of the global urban land area.

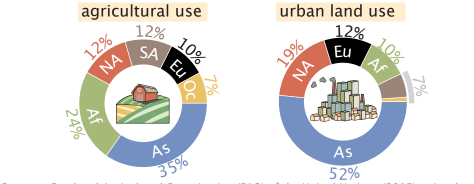

Sources: Food and Agricultural Organization (FAO) of the United Nations (2015) — Land Use [agricultural area]. Florczyk et al. 2019 — GHS Urban Centre Database 2015 [urban land area] Notes: Urban is defined as any inhabited area with ≥ 2500 residents, as defined by the USDA.

D THE LIVESTOCK POPULATION

The global population of terrestrial livestock is around 30 billion individuals, most of which are chickens. Asia houses most of the global livestock population, though South America and Europe harbor more animals on a per-capita basis.

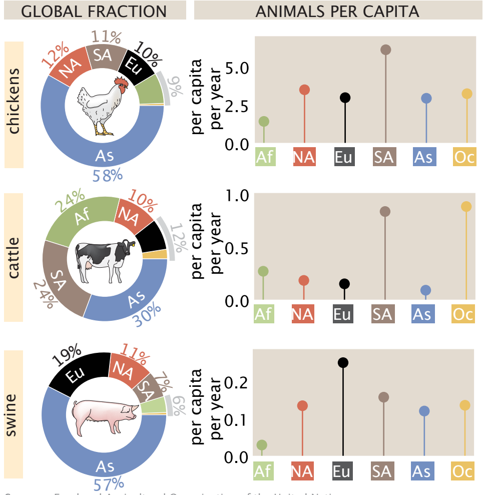

Sources: Food and Agricultural Organization of the United Nations

E WATER WITHDRAWAL

While Asia withdraws the most water for agricultural and municipal needs, North America withdraws the plurality of water for industrial purposes. North America also withdraws more water per capita than any other region.

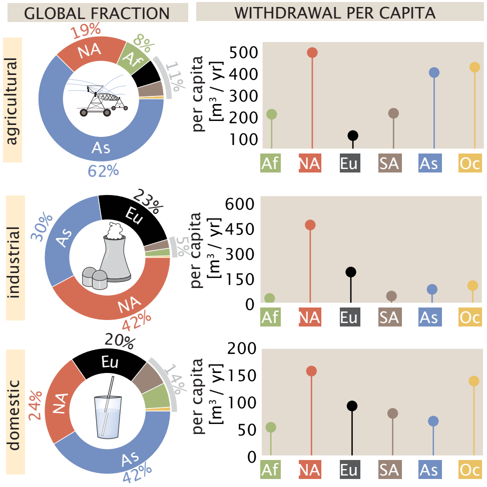

Source: AQUASTAT Main Database, Food and Agriculture Organization of the United Nations.
Notes: Values are reported directly from member countries and represent average of 2013–2017 period. Per capita values are computed given population of reporting countries.

F TREE COVERAGE AREA LOSS

Most drivers of tree coverage area loss are comparable in their effect at a global scale. However, there are drastic regional differences in the relative magnitudes.

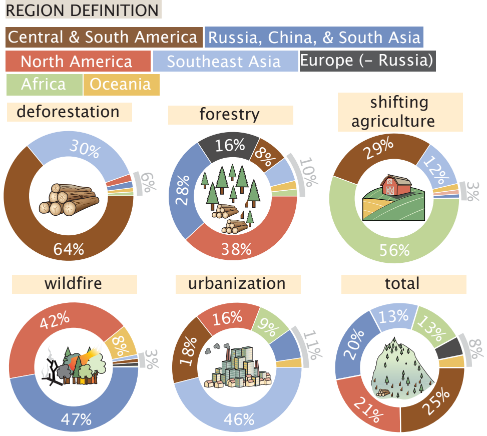

Source: Curtis et al. 2018 doi: 10.1126/science.aau3445.
Notes: Regions are as reported in Curtis et al. 2018. "Deforestation" here denotes permanent removal of tree cover for commodity production. "Shifting agriculture" here denotes forest/shrub land converted to agriculture and later abandoned. All values correspond to breakdown of cumulative tree cover area loss from 2001 – 2015.

G NITROGENOUS FERTILIZER USE & PRODUCTION

Modern agriculture requires nitrogen in amounts beyond what is produced naturally. Asia synthesizes and consumes a large majority of fixed nitrogen. However, Europe and North America dominate per capita synthesis whereas Oceania consumes more fertilizer per capita than any other region.

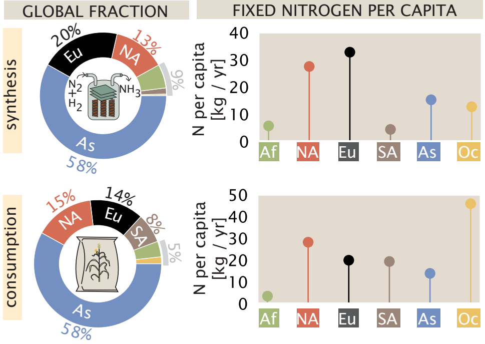

Source: Food and Agricultural Organization (FAO) of the United Nations.
Notes: Values account for reactive nitrogen production/consumption in context of fertilizer only and does not account for plastics, explosives, or other uses.

H GREENHOUSE GAS EMISSIONS

CO_2 and CH_4 are two potent greenhouse gases which are routinely emitted by anthropogenic processes such as burning fuel and rearing livestock. While Asia emits roughly half of all CO_2 and CH_4, North America and Oceania produce the most on a per capita basis, respectively.

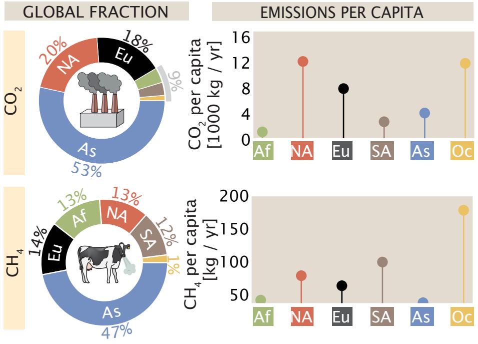

Sources: CO_2 data collated by: Friedlingstein, P. et al. (2019). doi: 10.5194/essd–11–1783–2019. See Panel K on Pg. 4 for complete list of sources. CH_4 data from Saunois et al, 2020 doi: 10.5194/essd–12–1561–2020 Notes: Values report decadal averages in kg CO_2 or CH_4 per year over time period 2008–2017.

I MATERIAL PRODUCTION

Humans excavate an enormous amount of material from the Earth's crust and transform it to build our structures. Two of these materials, concrete and steel, are produced primarily in Asia on both a global and per capita basis. Asia's per capita production of steel is only outpaced by Europe.

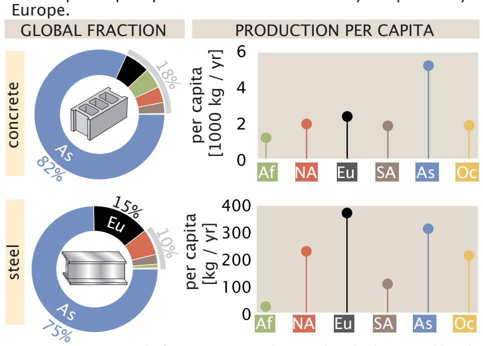

Sources: USGS Statistics and Information 2020, Steel Statistical Yearbook 2019 World Steel Association. Food and Agricultural Organization (FAO) of the United Nations — Annual Population. Notes: Reported values for cement and steel production corresponds to 2017 and 2018 values, respectively. Mass of concrete was calculated using a rule–of–thumb that 1 kg of cement yields 7 kg of concrete (Monteiro et al. 2017. doi: 0.138/nmat4930).

J POWER GENERATION AND CONSUMPTION

From heating water, to powering lights, to moving our vehicles, nearly every facet of modern human life requires the consumption of power, culminating in nearly 20 TW of power use in recent years. Asia consumes over half of the power derived from combustion of fossil fuels, with Europe and North America each consuming around 20% of the global total. Asia also produces the plurality of power from renewable technologies, such as hydroelectric, wind, and solar, however, North America, South America, and Europe each produce more on a per capita basis. Nuclear energy, however, is primarily produced in Europe, with North America and Asia coming in second and third place, respectively. On a per-capita basis, North America consumes or produces more energy than all other regions considered here, yielding a total power consumption of nearly 10,000 W per person.

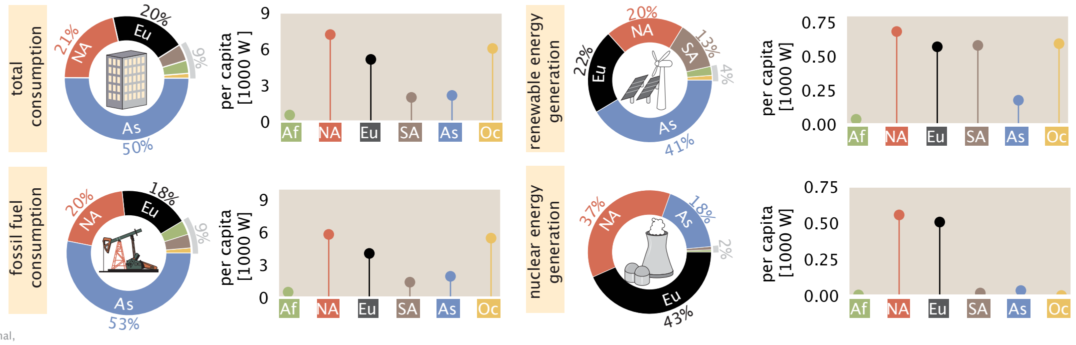

Source: Energy Information Administration of the United States (2017)
Notes: "Renewables" includes hydroelectric, biofuels, biomass (wood), geothermal, wind, and solar. "Fossil fuels" includes coal, oil, and natural gas.

The Anthropocene by the Numbers — Dynamics of Global Magnitudes

A THE HUMAN POPULATION

The human population has more than doubled in the past 60 years. During this time, the fraction of the population living in urban areas has steadily increased such that the global population is about evenly split between urban and rural environments.

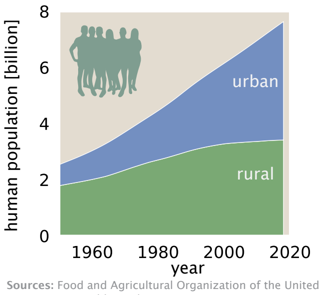

Sources: Food and Agricultural Organization of the United Nations – World Population
Notes: Urban/rural designation has no set definition and follows the conventions set by each reporting country.

B WATER WITHDRAWAL

Total water withdrawal has increased in concert with the human population, dominated by increasing agricultural use. Despite this increase, the average per-capita water use for agricultural and domestic purposes has remained largely constant for the past 40 years.

ANNUAL WATER WITHDRAWAL

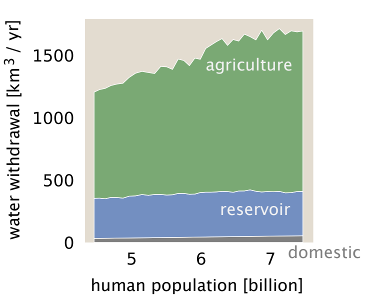

WITHDRAWAL PER CAPITA

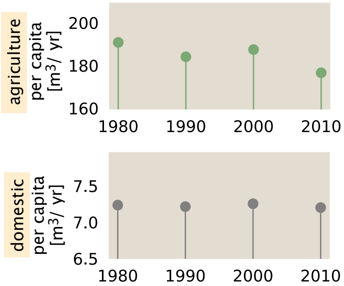

Source: AQUASTAT Main Database, Food and Agricultural Organization of the United Nations.
Notes: Values are reported directly from member countries and represent average of 2013–2017 period. Per capita values are computed given population of reporting countries.

C THE LIVESTOCK POPULATION

The standing population of livestock has been increasing, with chicken making up a large fraction of the total livestock population. The number of chicken raised per capita has increased since the 1960s, while cattle per capita have decreased.

STANDING POPULATION

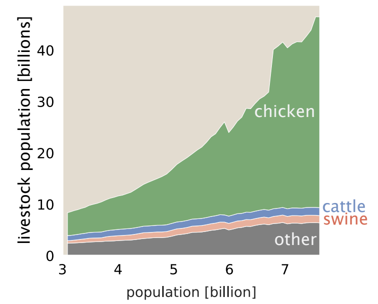

LIVESTOCK PER CAPITA

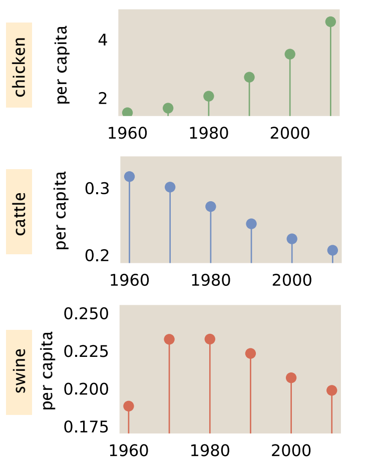

Sources: Food and Agricultural Organization of the United Nations

D MATERIAL PRODUCTION

Total mass of human-made materials hasbeen accumulating over time, dominated by construction materials. Per capita, the mass of bricks & asphalt, aggregates, and concrete has increased since the 1950s.

TOTAL ANTHROPOMASS

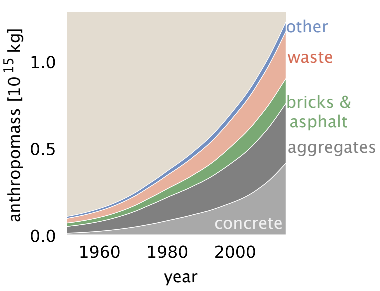

MASS PER CAPITA

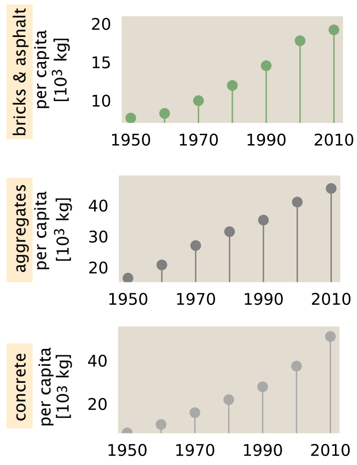

Sources: Krausmann et al. 2017 doi: 10.1073/pnas.1613773114
Notes: Material production is estimated from a material flows model.

E AQUATIC FOODS PRODUCTION

Aquatic (blue) foods production has been increasing with the human population. Interestingly, the mass produced from wild capture has remained constant per capita since the 1980s while the mass produced by aquaculture has increased per capita during the same period, driving the increase in overall production.

ANNUAL BLUE FOOD PRODUCTION

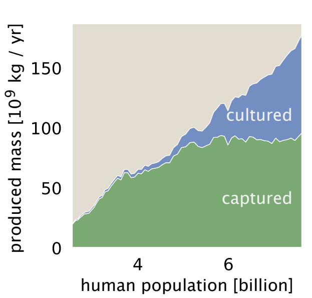

PRODUCTION PER CAPITA

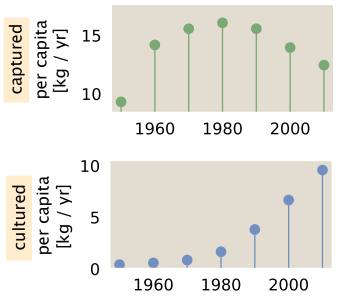

Sources: Food and Agricultural Organization of the United Nations

F POWER CONSUMPTION

Power consumption has increased with population, as well as technological and societal changes, which have driven an increase in power per capita across all generation types. The source of our power has also changed over time. Over the last 60 years, nuclear power has become comparable to hydroelectricity, with most of the growth occurring between 1970 and 1990. Renewable power generation is currently experiencing a similar growth pattern.

ANNUAL POWER CONSUMPTION

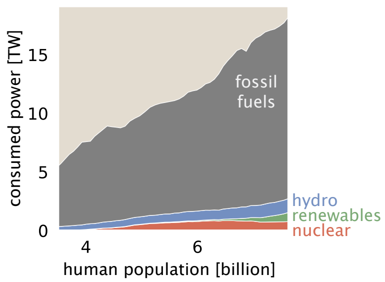

CONSUMPTION PER CAPITA

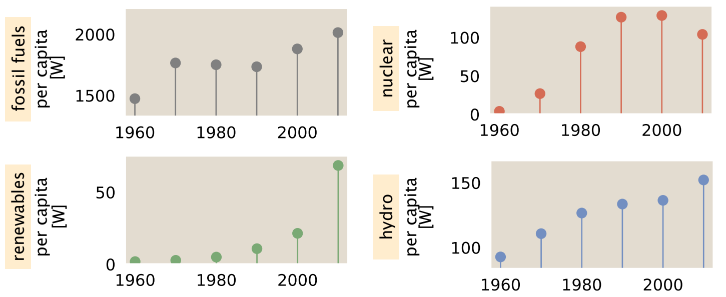

Source: Energy Information Administration of the United States (2017)
Notes: "Renewables" includes hydroelectric, biofuels, biomass (wood), geothermal, wind, and solar. "Fossil fuels" includes coal, oil, and natural gas.

G CO_2 EMISSIONS

Annual anthropogenic CO_2 emissions have been increasing with the population, driven by an increase in fossil fuel combustion. The amount of CO_2 emissions from fossil fuels has increased slightly per capita, while the per capita emissions from land use change have decreased.

ANNUAL CO_2 EMISSIONS

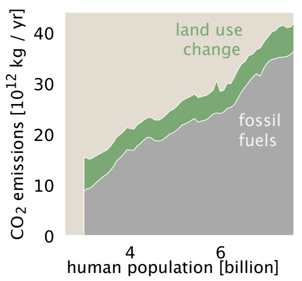

EMISSIONS PER CAPITA

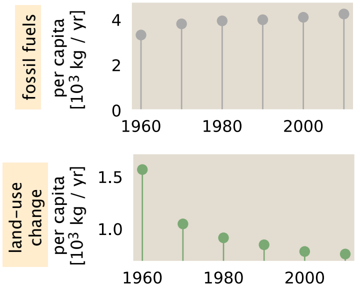

Sources: Data collated by: Friedlingstein, P. et al. (2019). doi: 10.5194/essd-11-1783-2019.
See Panel K on Pg. 4 for complete list of sources

H CH_4 EMISSIONS

While total anthropogenic methane (CH_4) emissions have been increasing with the human population, per capita emissions have been decreasing each decade since the 1970s. This per capita reduction reflects a shift in global diets away from methane-intenesive beef products, as well as better waste management policies in developed countries.

ANNUAL CH_4 EMISSIONS

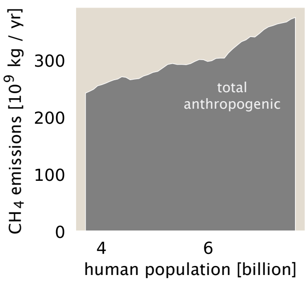

EMISSIONS PER CAPITA

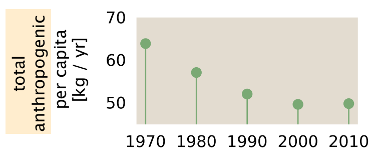

Sources: Saunois et al, 2020 doi: 10.5194/essd-12-1561-2020

The Anthropocene by the Numbers — Supporting Information

About: Here, we present citations and notes corresponding to each quantity assessed here. Each value presented on page 1 is assigned a Human Impacts Database identifier (HuID), accessible via anthroponumbers.org. When possible, primary data sources have been collated and stored as files in comma-separated-value (csv) format on the GitHub repository associated with this snapshot, accessible via DOI: 10.5281/zenodo.4453277 and https://github.com/rpgroup-pboc/human_impacts

A SURFACE TEMPERATURE

Surface temperature change relative to 1850-1900 average ≈ 1 - 1.4° C — HuID: 79598, 76539, 12147

Data Source(s): HadCRUT.4.6 (Morice et al., 2012, DOI: 10.1029/2011JD017187), GISTEMP v4 (GISTEMP Team, 2020: GISS Surface Temperature Analysis (GISTEMP), version 4. NASA Goddard Institute for Space Studies. Dataset accessed 2020-12-17 at https://data.giss.nasa.gov/gistemp/ & Lenssen et al., 2019, DOI: 10.1029/2018JD029522) and NOAAGlobalTemp v5 (Zhang et al, 2019, DOI: 10.1029/2019EO128229) datasets. Notes: The global mean surface temperature captures near-surface air temperature over the planet's land and ocean surface. The value reported represents the spread of the three estimates and their 95% confidence intervals. Temperature changes from all three datasets are expressed relative to the 1850-1900 average temperature from the HadCRUT.4.6 dataset. Since data for the period 1850-1880 are missing in GISTEMP v4 and NOAAGlobalTemp v5, data are centered by setting the 1880-1900 mean of all datasets to the HadCRUT.4.6 mean over the same period.

B ANNUAL ICE MELT

glaciers = (3.0 ± 1.2) × 10^{11} m³ / yr — HuID: 32459

Data Source(s): Intergovernmental Panel on Climate Change (IPCC) 2019 Special Report on the Ocean and Cryosphere in a Changing Climate. Table 2.A.1 on pp. 199-202. Notes: Value corresponds to the trend of annual glacial ice volume loss (reported as ice mass loss) from major glacierized regions (2006-2015) based on aggregation of observation methods (original data source: Zemp et al. 2019, DOI:10.1038/s41586-019-1071-0) with satellite gravimetric observations (original data source: Wouters et al. 2019, DOI:10.3389/feart.2019.00096). Ice volume loss was calculated from ice mass loss assuming a standard pure ice density of 920 kg / m³. Uncertainty represents a 95% confidence interval calculated from standard error propagation of the 95% confidence intervals reported in the original sources assuming them to be independent.

ice sheets = (4.7 ± 0.4) × 10^{11} m³ / yr — HuID: 95798, 93137

Data Source(s): D. N. Wiese et al. 2019 JPL GRACE and GRACE-FO Mascon Ocean, Ice, and Hydrology Equivalent HDR Water Height RL06M CRI Filtered Version 2.0, Ver. 2.0, PO.DAAC, CA, USA. Dataset accessed [2022-Feb-09]. DOI: 10.5067/TEM- SC-3MJ62

Notes: Value corresponds to the trends of combined annual ice volume loss (reported as ice mass loss) from the Greenland and Antarctic Ice Sheets (2002-2021) measured by satellite gravimetry. Ice volume loss was calculated from ice mass loss assuming a standard pure ice density of 920 kg / m³. Uncertainty represents one standard deviation and considers only propagation of monthly uncertainties in measurement.

Arctic sea ice = (3.0 ± 1.0) × 10^{11} m³ / yr — HuID: 89520

Data Source(s): PIOMAS Arctic Sea Ice Volume Reanalysis, Figure 1 of webpage as of January 31, 2022. Original method source: Schweiger et al. 2011, DOI:10.1029/2011JC007084 Notes: Value reported corresponds to the trend of annual volume loss from Arctic sea ice (1979-2022). The uncertainty in the trend represents the range in trends calculated from three ice volume determination methods.

C SEA ICE AREA

extent loss at yearly maximum cover (September) ≈ 4.8 × 10^{10} m² / yr — HuID: 66277

extent loss at yearly minimum cover (March) ≈ 0.4 × 10^{10} m² / yr — HuID: 66277

average annual extent loss ≈ 2.5× 10^{10} m² / yr — HuID: 66277

Data Source(s): Fetterer et al. 2017, updated daily. Sea Ice Index, Version 3, Boulder, Colorado USA. NSIDC: National Snow and Ice Data Center, DOI:10.7265/N5K072F8, [Accessed 2022-Feb-16]. Notes: Sea ice area is calculated by multiplying the percentage of sea ice in each pixel by pixel area and taking the integral sum of these products. Annual value corresponds to the linear trend of annual extent loss calculated by averaging over every month in a given year (2.45 × 10^{10} m² / yr, HuID: 66277). The minimum cover area loss corresponds to the linear trend of Arctic sea ice area in September from 1979-2021 and the maximum cover area loss corresponds to the linear trend of sea ice area in March from 1979-2021. The Antarctic sea ice area trend is not shown because a significant long-term trend over the satellite observation period is not observed and short-term trends are not yet identifiable.

D MATERIAL PRODUCTION

concrete production ≈ (2 - 3) × 10^{13} kg / yr — HuID: 25488, 81346, 16995

Data Source(s): United States Geological Survey (USGS) National Minerals Information Center, Commodity Statistics and Information, Cement Statistics and Information Miller et al., 2016 DOI:10.1088/1748-9326/11/074029. Monteiro et al. 2017, DOI:10.1038/nmat4930. Krausmann et al. 2017, DOI:10.1073/pnas.1613773114 Notes: Concrete is formed when aggregate material is bonded together by hydrated cement. The USGS reports the mass of cement produced in 2019 as 4.1 × 10^{12} kg in 2019. As most cement is used to form concrete, cement production can be used to estimate concrete mass using a multiplicative conversion factor of 7 (Monteiro et al.). Miller et al. report that the cement, aggregate and water used in concrete in 2012 sum to 2.3 × 10^{13} kg. Krausmann et al. report an estimated value from 2010 based on a material input, stocks, and outputs model. The value is net annual addition to concrete stocks plus annual waste and recycling to estimate gross production of concrete.

steel production ≈ 1.9× 10^{12} kg / yr — HuID: 51453, 44894, 85981

Data Source(s): United States Geological Survey (USGS) National Minerals Information Center, Commodity Statistics and Information, Iron and Steel Statistics and Information. World Steel Association, World Steel in Figures 2021, p.7. Notes: Crude steel includes stainless steels, carbon steels, and other alloys. The USGS reports the mass of crude steel produced in 2019 as 1.860 × 10^{12} kg. The World Steel Association reports a production value of 1.874 × 10^{12} kg in 2019. Krausmann et al. report an estimated value from 2010 based on a material input, stocks, and outputs model. The value is net annual addition to steel stocks plus annual waste and recycling to estimate gross production of steel.

plastic production ≈ 4 × 10^{11} kg / yr — HuID: 97241, 25437

Data Source(s): Geyer et al. 2017, Table S1, DOI:10.1126/sciadv.1700782. ; Krausmann et al. 2017, DOI:10.1073/pnas.1613773114. Notes: Value represents the approximate sum total global production of plastic fibers and plastic resin during the calendar year of 2015. Comprehensive data about global plastic production is sorely lacking. Geyer et al. draw data from various industry groups to estimate total production of different polymers and additives. Some of the underlying data is not publicly available, and data from financially-interested parties is inherently suspect. Krausmann et al. report an estimated value from 2010 based on a material input, stocks, and outputs model. The value is net annual addition to stocks plus annual waste and end-of-life recycling to estimate gross production of plastics.

E LIVESTOCK POPULATION

chicken standing population ≈ 3.5 × 10^{10} — HuID: 94934

cattle standing population ≈ 1.5 × 10^{9} — HuID: 92006

swine standing population ≈ 1.5 × 10^{9} — HuID: 21368

all livestock standing population ≈ 4.6× 10^{10} — HuID: 43599

Data Source(s): Food and Agriculture Organization (FAO) of the United Nations Statistical Database (2020) — Live Animals. Notes: Counts correspond to the estimated standing populations in 2019. Values are reported directly by countries. The FAO uses non-governmental statistical sources to address uncertainty and missing (non-reported) data. Reported values are therefore approximations.

F SYNTHETIC NITROGEN FIXATION

annual mass of synthetically fixed nitrogen ≈ (1.4 - 1.5)× 10^{11} kg N / yr — HuID: 60580, 61614

Data Source(s):United States Geological Survey (USGS) National Minerals Information Center, Commodity Statistics and Information, Nitrogen Statistics and Information International Fertilizer Association (IFA) Statistical Database (2021) Smith et al. 2020DOI:10.1039 /c9ee02873k Notes: Ammonia (NH_3) produced globally is compiled by the USGS and IFA from major factories that report output. The USGS estimates the approximate mass of nitrogen in ammonia produced in 2019 as 1.42 ×10^{11} kg N and the International Fertilizer Association reports a production value of 1.50 ×10^{11} kg N in 2019. Nearly all of this mass is produced by the Haber-Bosch process (>96%, Smith et al. 2020). In the United States, most of this mass is used for fertilizer, with the remainder being used to synthesize nitrogen-containing chemicals including explosives, plastics, and (≈ 88%, USGS Mineral Commodity Summaries 2020 - Nitrogen

G OCEAN ACIDITY

surface ocean [H+] ≈ 0.2 parts per billion — HuID: 90472

annual change in [H+] = 0.36 ± 0.03 % — HuID: 19394

Data Source(s): Figures 1-2 of European Environment Agency report CLIM 043 (2020). Original data source of the report is "Global Mean Sea Water pH" from Copernicus Marine Environment Monitoring Service. Notes: Reported value is calculated from the global average annual change in pH over years 1985-2018. The average oceanic pH was ≈ 8.057 in 2018 and decreases annually by ≈ 0.002 units, giving a change in [H+] of roughly $10^{-8.056}$ - $10^{-8.057}$ ≈ 4×10^{-11} mol/L or about 0.4% of the global average. [H+] is calculated as 10^{-pH} ≈ 10^{-8} mol/L or 0.2 parts per billion (ppb) which is calculated by noting that [H_2O] ≈ 55 mol / L. Uncertainty for annual change is the standard error of the mean.

H LAND USE

agricultural ≈ 5 × 10^{13} m² — HuID: 29582

Data Source(s): Food and Agriculture Organization (FAO) of the United Nations Statistical Database (2020) — Land Use. Notes: Agricultural land is defined as all land that is under agricultural management including pastures, meadows, permanent crops, temporary crops, land under fallow, and land under agricultural structures (such as barns). Reported value corresponds to 2017 estimates by the FAO.

urban ≈ (6 - 8) × 10^{11} m² — HuID: 41339, 39341

Data Source(s): Florczyk et al. 2019 (https://tinyurl.com/yyxxgtll) and Table 3 of Liu et al. 2018 DOI: 10.1016/j.rse.2018.02.055. Notes: Urban land area is determined from satellite imagery. An area is determined to be "urban" if the total population is greater than 5,000 and has a minimum population density of 300 people per km². Reported value gives the range of recent measurements of ≈ 6.5 × 10^{11} m² (2015) and ≈(7.5 ± 1.5) × 10^{11} m² (2010) from Florczyk et al. 2019 and Liu et al. 2018, respectively.

I RIVER FRAGMENTATION

global fragmented river volume ≈ 6 × 10^{11} m³ — HuID: 61661

Data Source(s): Grill et al. 2019 DOI: 10.1038/s41586-019-1111-9. Notes: Value corresponds to the water volume contained in rivers that fall below the connectivity threshold required to classify them as free-flowing. Value considers only rivers with upstream catchment areas greater than 10 km² or discharge volumes greater than 0.1 m³ per second. The ratio of global river volume in disrupted rivers to free-flowing rivers is approximately 0.9. The exact value depends on the cutoff used to define a "free-flowing" river. We direct the reader to the source for thorough detail.

J HUMAN POPULATION

urban-dwelling fraction of population ≈ 55% — HuID: 93995

total population ≈ 7.6 × 10^{9} — HuID: 85255

Data Source(s): Food and Agricultural Organization (FAO) of the United Nations Report on Annual Population, 2019. Notes: Value for total population in 2018 comes from a combination of direct population reports from country governments as well as inferences of underreported or missing data. The definition of "urban" differs between countries and the data does not distinguish between urban and suburban populations despite substantive differences between these land uses (Jones and Kammen 2013, doi: 10.1021/es4034364). As explained by the United Nations population division, "When the definition used in the latest census was not the same as in previous censuses, the data were adjusted whenever possible so as to maintain consistency." Rural population is computed from this fraction along with the total human population, implying that the total population is composed only of "urban" and "rural" communities.

K GREENHOUSE GAS EMISSIONS

anthropogenic CO_2 = (4.25 ± 0.33) × 10^{13} kg CO_2 / yr — HuID: 24789, 54608, 98043, 60670

Data Source(s): Table 6 of Friedlingstein et al. 2019, DOI: 10.5194/essd-11-1783-2019. Original data sources relevant to this study compiled in Friedlingstein et al.: 1) Gilfillan et al. https://energy.appstate.edu/CDIAC 2) Average of two bookkeeping models: Houghton and Nassikas 2017 DOI: 10.1002/2016GB005546; Hansis et al. 2015 DOI: 10.1002/2014GB004997) Dlugokencky and Tans, NOAA/GMLhttps://www.esrl.noaa.gov/gmd/ccgg/trends/. Notes: Value corresponds to total CO_2 emissions from fossil fuel combustion, industry (predominantly cement production), and land-use change during calendar year 2018. Emissions from land-use change are due to the burning or degradation of plant biomass. In 2018, 1.88 × 10^{13} kg CO_2 / yr accumulated in the atmosphere, reflecting the balance of emissions and CO_2 uptake by plants and oceans. Uncertainty corresponds to one standard deviation.

K GREENHOUSE GAS EMISSIONS (CONTINUED)

anthropogenic CH_4 = (3.4 – 3.9) × 10^{11} kg CH_4 / yr — HuID: 96837, 30725

Data Source(s): Table 3 of Saunois, et al. 2020. DOI: 10.5194/essd-12-1561-2020. Notes: Value corresponds to 2008-2017 decadal average mass of CH_4 emissions from anthropogenic sources. Includes emissions from agriculture and lalndfill, fossil fuels, and burning of biomass and biofuels, but other inventories of anthropogenic methane emissions are also considered. Reported range represents the minimum and maximum estimated emissions from a combination of "bottom-up" and "top-down" models.

anthropogenic N_2O = 1.1 (+0.6, – 0.5) × 10^{10} kg N_2O / yr — HuID: 44575

Data Source(s): Table 1 of Tian, H., et al. 2020. DOI: 10.1038/s41586-020-2780-0. Notes: Value corresponds to annualized N_2O emissions from anthropogenic sources in the years 2007-2016. The value reported in the source is 7.3 (4.2, 11.4) Tg N / year. This is converted to a mass of N_2O using the fact that N ≈ 14/22 of the mass of N_2O. Reported value is mean with the uncertainty bounds (+,–) representing the maximum and minimum values observed in the 2007-2016 time period.

L WATER WITHDRAWAL

agricultural withdrawal = 1.3 × 10^{12} m³ / yr	HuID: 84545, 43593, 95345
industrial withdrawal = 5.9 × 10^{11} m³ / yr	HuID: 27142
domestic withdrawal = 5.4 × 10^{10} m³ / yr	HuID: 69424
total withdrawal = (1.7 – 2.2) × 10^{12} m³ / yr	HuID: 27342, 68004

Data Source(s): Figure 1 of Qin et al. 2019. DOI: 10.1038/s41893-019-0294-2. AQUASTAT Main Database, Food and Agriculture Organization of the United Nations Notes: "Agricultural" and "total" withdrawal include one value from Qin et al. (who reports "consumption") and one value from the AQUASTAT database. Industrial water withdrawal is from AQUASTAT and domestic withdrawal value is from Qin et al. Values in AQUASTAT are self-reported by countries and have missing values from some countries, probably accounting for a few percent underreporting. All values represent withdrawals. For agricultural and domestic, water withdrawal is assumed to be the same as water consumption as reported in Qin et al.

M SEA LEVEL RISE

added water = 1.97 (+0.36, –0.34) mm / yr	HuID: 97108
thermal expansion = 1.19 (+0.25, –0.24) mm / yr	HuID: 97688
total observed sea-level rise = 3.35 (+0.47, –0.44) mm / yr	HuID: 81373

Data Source(s): Table 1 of Frederikse et al. 2020. DOI:10.1038/s41586-020-2591-3. Notes: Values correspond to the average global sea level rise for the years 1993 – 2018. "Added water" (barystatic) change includes effects from meltwater from glaciers and ice sheets, added mass from sea-ice discharge, and changes in the amount of terrestrial water storage. Thermal expansion accounts for the volume change of water with increasing temperature. Values for "thermal expansion" and "added water" come from direct observations of ocean temperature and gravimetry/altimetry, respectively. Total sea level rise is the observed value using a combination of measurement methods. "Other sources" reported on page 1 accounts for observed residual sea level rise not attributed to a source in the model. Values in brackets correspond to the upper and lower bounds of the 90% confidence interval.

N TOTAL POWER USE

global power use ≈ 19 – 20 TW — HuID: 31373, 85317

Data Source(s): bp Statistical Review of World Energy, 2020; U.S. Energy Information Administration, 2020. Notes: Value represents the sum of total primary energy consumed from oil, natural gas, coal, and nuclear energy and electricity generated by hydroelectric and other renewables. Value is calculated using annual primary energy consumption as reported in data sources assuming uniform use throughout a year, yielding ≈ 19 – 20 TW.

O TREE COVERAGE AREA LOSS

commodity-driven deforestation = (5.7 ± 1.1) × 10^{10} m² / yr	HuID: 96098
forestry = (5.4 ± 0.8) × 10^{10} m² / yr	HuID: 38352
urbanization = (2 ± 1) × 10^9 m² / yr	HuID: 19429
shifting agriculture = (7.5 ± 0.9) × 10^{10} m² / yr	HuID: 24388
wildfire = (7.2 ± 1.3) × 10^{10} m² / yr	HuID: 92221
total loss ≈ 2 × 10^{11} m² / yr	HuID: 78576

Data Source(s): Table 1 of Curtis et al. 2018 DOI:10.1126/science.aau3445. Hansen et al. 2013 DOI:10.1126/science.1244693. Global Forest Watch, 2020. Reported values in source correspond to total loss from 2001 – 2015. Values given are averages over this 15 year window. Notes: Commodity-driven deforestation is "long-term, permanent, conversion of forest and shrubland to a non-forest land use such as agriculture, mining, or energy infrastructure." Forestry is defined as large-scale operations occurring within managed forests and tree plantations with evidence of forest regrowth in subsequent years. Urbanization converts forest and shrubland for the expansion and intensification of existing urban centers. Disruption due to "shifting agriculture" is defined as "small- to medium-scale forest and shrubland conversion for agriculture that is later abandoned and followed by subsequent forest regrowth". Disruption due to wildfire is "large-scale forest loss resulting from the burning of forest vegetation with no visible human conversion or agricultural activity afterward". Uncertainty corresponds to the 95% confidence interval. Uncertainty is approximate for "urbanization" as the source reports an ambiguous error of "± <1%".

P POWER FROM FOSSIL FUELS

natural gas = 4.5 – 4.9 TW	HuID: 49947, 86175
oil = 6.1 – 6.6 TW	HuID: 4121, 39756
coal = 5.0 – 5.6 TW	HuID: 10400, 60490
total = 16 – 17 TW	HuID: 29470, 29109

Data Source(s): bp Statistical Review of World Energy, 2022. U.S. Energy Information Administration, 2020. Notes: Values are self-reported by countries. values from bp Statistical Review and EIA correspond to 2019. Reported TW are computed from primary energy (e.g. kg coal) units assuming uniform use throughout the year. Oil volume includes crude oil, shale oil, oil sands, condensates, and natural gas liquids separate from specific natural gas mining. Natural gas value excludes gas flared or recycled and includes natural gas produced for gas-to-liquids transformation. Coal value includes 2019 value exclusively for solid commercial fuels such as bituminous coal and anthracite, lignite and subbituminous coal, and other solid fuels. This includes coal used directly in power production as well as coal used in coal-to-liquids and coal-to-gas transformations.

Q POWER FROM RENEWABLE RESOURCES

wind ≈ 0.36– 0.43 TW	HuID: 30581, 85919
solar ≈ 0.18 – 0.21 TW	HuID: 99885, 58303
hydroelectric = 1.2 TW	HuID: 15765, 50558
total renewable power ≈ 1.9 – 2.1 TW	HuID: 75741, 20246

Data Source(s): bp Statistical Review of World Energy, 2022. U.S. Energy Information Administration, 2020. Notes: Reported values correspond to estimates for the 2019 calendar year. Renewable resources are defined as wind, geothermal, solar, biomass and waste. Hydroelectric, while presented here, is not defined as a renewable in the bp dataset. All values are reported as input-equivalent energy, meaning the input energy that would have been required if the power was produced by fossil fuels. bp reports that fossil fuel efficiency used to make this conversion was about 40% in 2017.

R FOSSIL FUEL EXTRACTION

volume of natural gas = (3.9 – 4.0) × 10^{12} m³ / yr	HuID: 11468, 20532
volume of oil = (5.5 ± 5.8) × 10^9 m³ / yr	HuID: 66789, 97719
mass of coal = (7.8 – 8.1) × 10^{12} kg / yr	HuID: 78435, 48928

Data Source(s): bp Statistical Review of World Energy, 2022. U.S. Energy Information Administration, 2020. Notes: Oil volume includes crude oil, shale oil, oil sands, condensates, and natural gas liquids separate from specific natural gas mining. Natural gas value excludes gas flared or recycled and includes natural gas produced for gas-to-liquids transformation. Coal value includes solid commercial fuels such as bituminous coal, anthracite, lignite, subbituminous coal, and other solid fuels. All values correspond to 2019 estimates.

S OCEAN WARMING

heat uptake by ocean ≈ 346 ± 51 TW	HuID: 94108
upper ocean (0 – 700 m) temperature increase since 1960 = 0.18 – 0.2 °C	HuID: 69674 72086

Data Source(s): Table S1 of Cheng et al. 2017. doi: 10.1126/sciadv.1601545. NOAA National Centers for Environmental Information, 2020. doi:10.1029/2012GL051106. Notes: Heat uptake reported is the average over time period 1992-2015 with 95% confidence intervals. Range of temperatures reported captures the 95% confidence interval of temperature increase for the period 2015-2019 with respect to the 1958-1962 mean. Temperature change is considered in the upper 700 m because sea surface temperatures have high decadal variability and are a poor indicator of ocean warming; see Roemmich et al. 2015, doi: 10.1038/NCLIMATE2513.

T POWER FROM NUCLEAR FISSION

nuclear power ≈ 0.79–0.92 TW — HuID: 48387

Data Source(s): bp Statistical Review of World Energy, 2020. U.S. Energy Information Administration, 2022. Notes: Values are self-reported by countries and correspond to estimates for 2019. Values are reported as 'input-equivalent' energy, meaning the energy that would have been needed to produce a given amount of power if the input were a fossil fuel, which is converted to TW here. This is calculated by multiplying the given power by a conversion factor representing the efficiency of power production by fossil fuels. In 2017, this factor was about 40%.

U NUCLEAR FALLOUT

anthropogenic ^{239}Pu and ^{240}Pu from weapons testing ≈ 1.4 × 10^{11} kg / yr — HuID: 42526

Data Source(s): Table 1 in Hancock et al. 2014 doi: 10.1144/SP395.15. Fallout in activity from UNSCEAR 2000 Report on Sources and Effects of Ionizing Radiation Report to the UN General Assembly -- Volume 1. Notes: The approximate mass of Plutonium isotopes ^{239}Pu and ^{240}Pu released into the atmosphere from the ≈ 500 above-ground nuclear weapons tests conducted between 1945 and 1980. Naturally occurring ^{239}Pu and ^{240}Pu are rare, meaning that nearly all contemporary labile plutonium comes from human production. (Taylor 2001,doi: 10.1016/S1569-4860(01)80003-6) The total mass of radionuclides released is ≈ 3300 kg with a combined radioactive fallout of ≈11 PBq. These values do not represent the entire $^{239+240}$Pu globally distributed mass as it excludes non-weapons sources.

V CONTEMPORARY EXTINCTION

animal species extinct since 1500 > 750	HuID: 44641
plant species extinct since 1500 > 120	HuID: 86866

Data Source(s): The IUCN Red List of Threatened Species. Version 2020-2. Notes: Values correspond to absolute lower-bound count of animal extinctions caused over the past ≈ 520 years. Of the predicted ≈ 8 million animal species, the IUCN databases catalogues only ≈ 900,000 with only ≈ 75,000 being assigned a conservation status. Representation of plants and fungi is even more sparse with only ≈40,000 and ≈285 being assigned a conservation status, respectively. The number of extinct animal species is undoubtedly higher than these reported values, as signified by an inequality symbol (>).

W EARTH MOVING

waste and overburden from coal mining ≈ 6.5 × 10^{13} kg / yr	HuID: 72899
earth moved from urbanization > 1.4 × 10^{14} kg / yr	HuID: 59640

Data Source(s): Supplementary table 1 of Cooper et al. 2018. DOI: doi.org/gfwfhd. Notes: Coal mining waste and overburden mass is calculated given commodity-level stripping ratios (mass of overburden/waste per mass of coal resource mined) and reported values of global coal production by type. Urbanization mass is presented as a lower bound estimate of the mass of earth moved from global construction projects. This comes from a conservative estimate that the ratio of the mass of earth moved per mass of cement/concrete used in construction globally is 2:1. This value is highly context dependent and we encourage the reader to read the source material for a more thorough description of this estimation.

erosion from agricultural land > 1.2 – 2.4 × 10^{13} kg / yr — HuID: 19415, 41496

Data Source(s): Pg. 377 of Wang and Van Oost 2019. DOI: 10.1177/0959683618816499. Pg. 21996 of Borrelli et al. 2020 DOI: 10.1073/pnas.2001403117. Notes: Cumulative sediment mass loss over history of human agriculture due to accelerated erosion is estimated to be ≈ 30,000 Gt. Recent years have an estimated erosion rate ranging from 12 Pg / yr (Wang and Van Oost) to ≈ 24 Pg / yr (Borrelli et al.). Values come from computational models conditioned on time-resolved measurements of sediment deposition in catchment basins.

We are incredibly grateful for the generosity of a wide array of experts for their advice, suggestions, and criticism of this work. Specifically, we thank Suzy Beeler, Lars Bildsten, Justin Bois, Chris Bowler, Matthew Burgess, Ken Caldeira, Jörn Callies, Sean B. Carroll, Ibrahim Cissé, Joel Cohen, Michelle Dan, Bethany Ehlmann, Gidon Eshel, Paul Falkowski, Daniel Fisher, Thomas Frederikse, Neil Fromer, Eric Galbraith, Lea Goentoro, Evan Groover, John Grotzinger, Soichi Hirokawa, Greg Huber, Christina Hueschen, Bob Jaffe, Elizabeth Kolbert, Thomas Lecuit, Raphael Magarik, Jeff Marlow, Brad Marston, Jitu Mayor, Elliot Meyerowitz, Lisa Miller, Dianne Newman, Luke Oltrogge, Nigel Orme, Victoria Orphan, Marco Pasti, Pietro Perona, Noam Prywes, Stephen Quake, Hamza Raniwala, Manuel Razo-Mejia, Thomas Rosenbaum, Benjamin Rubin, Alex Rubinsteyn, Shyam Saladi, Tapio Schneider, Murali Sharma, Alon Shepon, Arthur Smith, Matthieu Talpe, Wati Taylor, Julie Theriot, Tadashi Tokieda, Cat Triandifillou, Sabah Ul-Hasan, Tine Valencic, and Ned Wingreen. We also thank Yue Qin for sharing data related to global water consumption. Many of the topics in this work began during the Applied Physics 150C course taught at Caltech during the early days of the COVID-19 pandemic. This work was supported by the Resnick Sustainability Institute at Caltech and the Schwartz-Reisman Collaborative Science Program at the Weizmann Institute of Science.
