# Supplementary material for: Anthroponumbers.org: A quantitative database of human impacts on Planet Earth
Source: Patterns (N Y). 2022 Aug 3;3(9):100552. doi: 10.1016/j.patter.2022.100552 (PMC9481956; doi:10.1016/j.patter.2022.100552)
Supplement: Document S3. Article plus supplemental information [file mmc3.pdf]

Descriptor

Patterns

Anthroponumbers.org: A quantitative database of human impacts on Planet Earth

Graphical abstract

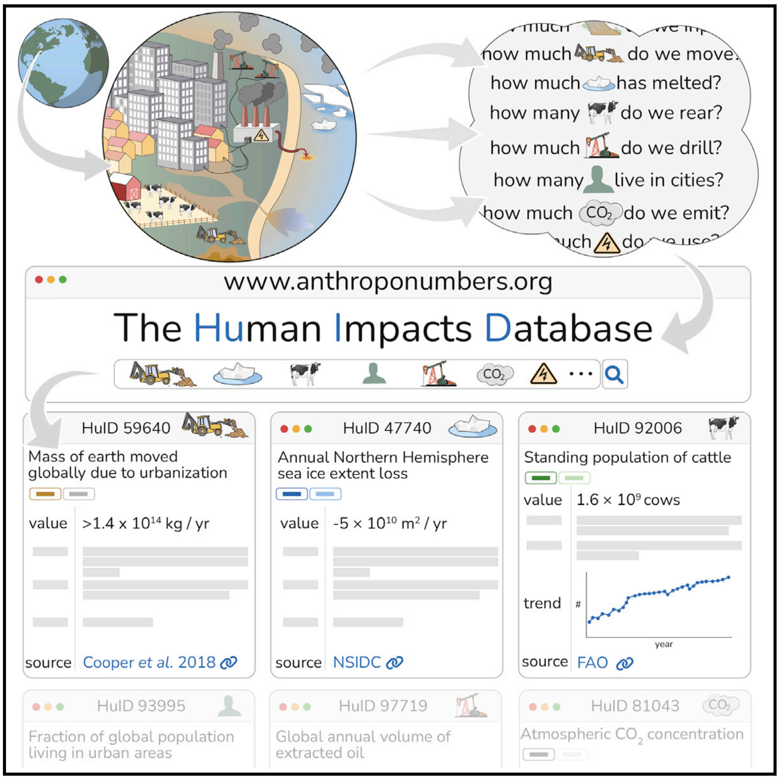

Highlights

- We present a holistic view of the many ways humans alter Earth at a global scale
- We consider how these global quantities vary across geography
- We further explore the time- and population-dependent dynamics of these impacts
- We enumerate and describe key properties associated with each entry in the database

Authors

Griffin Chure, Rachel A. Banks, Avi I. Flamholz, ..., Yinon Bar-On, Ron Milo, Rob Phillips

Correspondence

griffinchure@gmail.com (G.C.), phillips@pboc.caltech.edu (R.P.)

In brief

The environmental impacts of human action on Earth are being felt on many fronts. Despite our deep knowledge of these impacts, finding reliable quantitative information is burdensome, often requiring domain expertise and programmatic acumen. We present the Human Impacts Database, which houses a diverse array of quantities regarding human impacts, making them easily accessible and searchable. We use this database to present a broad view of the Anthropocene, exploring the global magnitudes, spatial dependence, and temporal dynamics of human impacts.

Chure et al., 2022, Patterns *3*, 100552
September 9, 2022 © 2022 The Author(s).
https://doi.org/10.1016/j.patter.2022.100552

CellPress

CellPress
OPEN ACCESS

Descriptor

Anthroponumbers.org: A quantitative database of human impacts on Planet Earth

Griffin Chure,[1,2,10,11,*] Rachel A. Banks,[3,4,5,10] Avi I. Flamholz,[3,4] Nicholas S. Sarai,[6] Mason Kamb,[5] Ignacio Lopez-Gomez,[4,7] Yinon Bar-On,[8] Ron Milo,[8] and Rob Phillips[3,5,9,*]
[1]Department of Biology, Stanford University, Stanford, CA, USA
[2]Department of Applied Physics, California Institute of Technology, Pasadena, CA, USA
[3]Division of Biology and Biological Engineering, California Institute of Technology, Pasadena, CA, USA
[4]Resnick Sustainibility Institute, California Institute of Technology, Pasadena, CA, USA
[5]Chan-Zuckerberg BioHub, San Francisco, CA, USA
[6]Division of Chemistry and Chemical Engineering, California Institute of Technology, Pasadena, CA, USA
[7]Department of Environmental Science and Engineering, California Institute of Technology, Pasadena, CA, USA
[8]Department of Plant and Environmental Sciences, Weizmann Institute of Science, Rehovot, Israel
[9]Department of Physics, California Institute of Technology, Pasadena, CA, USA
[10]These authors contributed equally
[11]Lead contact
*Correspondence: griffinchure@gmail.com (G.C.), phillips@pboc.caltech.edu (R.P.)
https://doi.org/10.1016/j.patter.2022.100552

THE BIGGER PICTURE Over the last 10,000 years, human activities have transformed Earth through farming, forestry, mining, and industry. The complex results of these activities are now observed and quantified as "human impacts" on Earth's atmosphere, oceans, biosphere, and geochemistry. While myriad studies have explored facets of human impacts on the planet, they are necessarily technical and often highly focused. Thus, finding reliable quantitative information requires a significant investment of time to assess each quantity and associated uncertainty. We present the Human Impacts Database (www.anthroponumbers.org), which houses a diverse array of such quantities. We review a subset of these values and how they help build intuition for understanding the Earth-human system. While collation alone does not tell us how to best ameliorate human impacts, we contend that any future plans should be made in light of a quantitative understanding of the interconnected ways in which humans influence the planet.

1 2 3 4 **5** **Production:** Data science output is well understood and (nearly) universally adopted

SUMMARY

The Human Impacts Database (www.anthroponumbers.org) is a curated, searchable resource housing quantitative data relating to the diverse anthropogenic impacts on our planet, with topics ranging from sea-level rise to livestock populations, greenhouse gas emissions, fertilizer use, and beyond. Each entry in the database reports a quantitative value (or a time series of values) along with clear referencing of the primary source, the method of measurement or estimation, an assessment of uncertainty, and links to the underlying data, as well as a permanent identifier called a Human Impacts ID (HuID). While there are other databases that house some of these values, they are typically focused on a single topic area, like energy usage or greenhouse gas emissions. The Human Impacts Database facilitates access to carefully curated data, acting as a quantitative resource pertaining to the myriad ways in whic h humans have an impact on the Earth, for practicing scientists, the general public, and those involved in education for sustainable development alike. We outline the structure of the database, describe our curation procedures, and use this database to generate a graphical summary of the current state of human impacts on the Earth, illustrating both their numerical values and their intimate interconnections.

This is an open access article under the CC BY license (http://creativecommons.org/licenses/by/4.0/).

INTRODUCTION

One of the most important scientific developments of the last two centuries is the realization that the evolution of Earth is deeply intertwined with the evolution of life. Perhaps the most famous example of this intimate relationship is the large-scale oxygenation of Earth's atmosphere following the emergence of photosynthesis.[1] This dramatic change in the composition of the atmosphere is believed to have caused a massive extinction, as the biosphere was not adapted to an oxygenated atmosphere.[2–4] Over the past 10,000 years, humans have likewise transformed the planet, directly affecting the rise and fall of ecosystems,[5–13] the pH and surface temperature of the oceans,[14,15] the composition of terrestrial biological and human-made mass,[16,17] the planetary albedo and ice cover,[18–27] and the chemistry of the atmosphere,[28–33] to name just a few examples. The breadth of human impacts on the planet is so diverse that it touches on nearly every facet of the Earth system and every scientific discipline.

Technological advances in remote sensing, precision measurement, and computational power have made it possible to measure these anthropogenic impacts with unprecedented depth and resolution. However, as scientists with different training use distinct methods for measurement and analysis, report data in different units and formats, and use nomenclature differently, these studies can be very challenging to understand and relate to one another. Even seemingly simple questions such as "how much water do humans use?" can be difficult to answer when search engines are not optimized for finding numeric data, and a search of the scientific literature yields an array of complicated analyses with different units, varying definitions about what constitutes water use, and distinct approaches to quantifying flows. This problem persists beyond the primary scientific literature, as governmental, intergovernmental, and industry datasets can be similarly tricky and laborious to interpret.

Writing from California, as several of the authors are, where we now have a "wildfire season" and a multi-decadal drought,[34,35] we wanted to develop a deeper understanding of the ways in which human activities might have produced such dramatic and consequential changes in our local and global environment. In pursuit of basic understanding, we asked many questions, like "how much water and land do humans use?" and "how much methane is emitted annually?" In our search for answers, even when the question is well defined (as is the case for methane emissions), we often encountered the same challenges: disparate technical studies written for expert audiences must be understood, evaluated, and synthesized just to answer simple questions. It seemed to us that a referenced compendium of "things we already know," akin to the *CRC Handbook of Chemistry and Physics*, would be very useful for us and others.

In building the Human Impacts Database, we took inspiration from our previous experience building and using the BioNumbers Database[36] (https://bionumbers.hms.harvard.edu), a compendium of quantitative values relating to cell and organismal biology. Over the past decade, the BioNumbers Database has become a widely accessed resource that serves not only as an index of biological numbers, but also as a means of finding relevant primary literature, learning about methods of measurement, and teaching basic concepts in cell biology.[37]

We believe that a centralized, searchable database for quantitative data encompassing the breadth of human impacts on Earth would be similarly transformative for researchers, students, and the interested public. While reading an entry in the Human Impacts Database is not a replacement for reading the primary literature, the database serves as a resource to expedite the process of finding quantitative data and exploring their interconnection. Importantly, we do not put forward projected scenarios or specific policy proposals for combating anthropogenic effects on Earth. However, we are convinced that such proposals should be evaluated in the light of a comprehensive and quantitative understanding of the Earth-human system.

RESULTS

Finding and compiling numbers from scientific literature, governmental and non-governmental reports, and industrial datasets

We have established the Human Impacts Database (http://anthroponumbers.org) as a repository for the rapid discovery of quantities describing the Earth-human system. We here provide a more complete description of the database structure, the values it holds, and the stories it tells us about how humans affect the Earth. As of this writing, the database holds > 300 unique and manually curated entries covering a breadth of data sources, including primary scientific literature, governmental and non-governmental reports, and industrial communiques. Before it is added to the database and made public, each entry is vetted extensively by the administrators (see Note S1 for detailed curation procedures). Included in each entry is a summary of the method by which it was determined, an assessment of the corresponding uncertainty, and an explicit statement of any known caveats important for interpretation of the data. While these ≈ 300 entries include those we consider to be essential for a quantitative understanding of human impacts on Earth, it is not an exhaustive list. This database will continue to grow and evolve as more data become publicly released, our understanding of the human-Earth system improves, and members of the scientific community suggest values to be added.

Figure 1 shows the Human Impacts Database Entry for perhaps the most emblematic anthropogenic impact: the standing atmospheric CO_2 concentration. The first two components of an entry are the quantity title and its assigned category and subcategory (Figures 1A and 1B). Primary categorization falls into one of five classes: "land," "water," "energy," "flora & fauna," and "atmospheric & biogeochemical cycles." Of course, these categories are broad, and entries can be associated with several categories. For this reason, each entry is also assigned a narrower "subcategory," such as "agriculture," "urbanization," or "carbon dioxide." While this categorization is not meant to be exhaustive, and many other schemes could be implemented, we found that this organization allowed us to quickly browse and identify quantities of interest.

Following the title and categorization, we report the measured atmospheric CO_2 concentration. This corresponds to the most recent reported measurement, which is, as of this writing, roughly 416 parts per million (ppm) in 2021 (Figure 1C). Importantly, we report an approximate value for the CO_2 concentration rather than a precise value to many significant digits. While the most

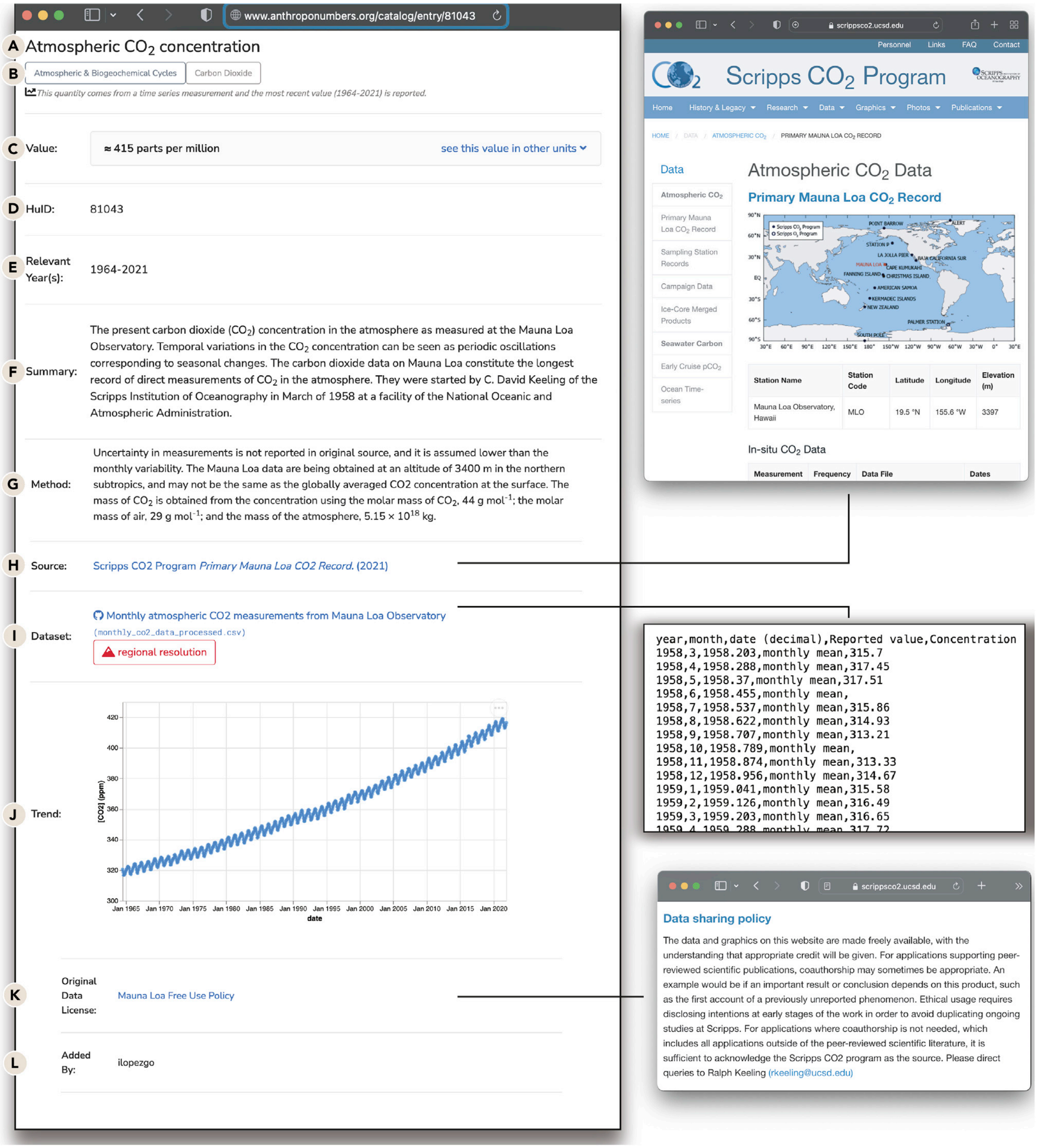

Figure 1. A representative entry in the Human Impacts Database

(A–I) The entry page for HuID 81043, "Atmospheric CO_2 concentration," is diagrammed with important features highlighted. Each entry in the Human Impacts Database has (A) a name, (B) a primary and secondary categorization, (C) the numerical value with other units when appropriate, (D) a five-digit permanent numeric identifier, (E) the years for which the measurement was determined, (F) a brief summary of the quantity, (G) the method of determination, (H) a link to the source data, and (I) a link to a processed version of the data saved as a .csv file. When possible, a time series of the data is presented.

(K) Every entry in the database also has a statement of the data use protection associated with the relevant data. When possible, this links directly to the data protection statement from the original source. In other cases, it points to the formal definition of the license by a disinterested third party.

(L) Finally, each entry lists the username of the administrator who curated the quantity. Their contact information is available on the anthroponumbers.org "About" page.

recent entry in the linked dataset (Figure 1I) gives a monthly average value of 416.43 ppm for December of 2021, this value does not account for error in the measurement, fluctuations throughout December, or seasonal oscillations in atmospheric CO_2. Therefore, we report a rounded value of 416 ppm. CO_2 measurements are quite accurate, but other measurements and inferences recorded in the Human Impacts Database are less so. We therefore strive to give an assessment of the uncertainty for all values. This can be in the form of a confidence interval, as for the entry for the global mean sea-level rise since 1900 due to thermal expansion, which reports a 90% confidence interval, or bounds on the value, as for the number of contemporary animal extinctions since 1500 CE, which reports only a lower bound. In addition to error assessment, we also aim to provide legible units for all entries. Although atmospheric CO_2 is commonly reported in ppm units, we also report this value in other equivalent units, including the mole and mass fractions of CO_2 and the total mass of CO_2 in the atmosphere in kg CO_2 (Figure 1C). Whenever possible, entries will report values in multiple units to make quantities accessible to readers coming from diverse backgrounds. Furthermore, in many cases, the global value is aggregated from local measurements. We flag entries for which regional data broadly defined are available in the database GitHub repository.

Following the numerical value is the permanent Human Impacts Database identifier, which we abbreviate as HuID (Figure 1D). The HuID is a randomly generated five-digit integer that serves as a permanent and static identifier that can be used for in-line referencing. Rather than identifying a single value, we consider the HuID a pointer to a particular *entry*, so that HuID 81043 can be used to reference the atmospheric CO_2 concentration in 2021 and 1980 (Figure 1E). For example, to reference the present-day atmospheric CO_2 concentration, one could report the value as "$\approx$ 416 ppm (HuID 81043:2021)." In addition, since each entry comes from a single source, we may have more than one HuID reporting similar quantities. For example, HuIDs 69674 and 72086 report recent measurements of the temperature of the upper ocean.

The "Summary" field (Figure 1F) gives a succinct description of the quantity and its relationship to "human impacts" broadly construed, along with other pertinent information. This could include a more detailed definition of terms used in the quantity, such as the entry for "sea ice extent loss in March," which defines the term "sea ice extent," or useful historical information about the measurement. In our example of atmospheric CO_2 concentration, the summary explains that the measurement is made at the Mauna Loa observatory and points out the seasonal oscillations that are observed. The following "Method" field describes the method by which the quantity was measured, inferred, or estimated (Figure 1G). This field also provides an assessment of the uncertainty in the value, which may include a description of how confidence intervals were computed or a list of critical assumptions that were made to estimate missing data.

All fields through "Method" (Figures 1A–1G) depend on manual curation and interpretation by database administrators. The following two fields, "Source" and "Dataset" (Figures 1H and 1I), provide direct links to the primary source reference and the relevant data. Both of these fields are direct links (shown as insets in Figure 1). The "Source" field can point to either the published scientific literature or the resource page of a governmental, industrial, or non-governmental organization data deposition URL. The "Dataset" field links directly to either a CSV format of the data or to a folder with global and regional values within the corresponding GitHub repository. As discussed in Note S1, the vast majority of these data files have been converted into a "tidy-data" format[38] by database administrators, which maximizes programmatic readability.

When possible, a graphical time series of the data is also presented as an interactive plot (Figure 1J). These plots enable users to quickly apprehend time-dependent trends in the data without downloading or processing the dataset. The data sources we rely on in building the database are remarkably varied, coming from governmental, industrial, and primary scientific sources, each with their own specific data use protection policies. Each entry (Figure 1K) also provides a link to the data use policy for each individual dataset. While not available for every entry, the majority of quantities we have curated in the Human Impacts Database contain measurements over time. The last field gives the username of the administrator who generated this entry (Figure 1L). Their affiliation and contact information are available on the database's "About" page. We invite the reader to contact the administrators collectively—through our "Contact" page or directly through our personal emails as provided on the "About" page—with questions, concerns, or suggestions.

While Figure 1 is a representative example, each quantity in the Human Impacts Database tells a different story. Easy and centralized access to different entries allows users to learn about the magnitude of human impacts and also study the interactions between different human activities, which, as we discuss in the next section, are deeply intertwined.

Global magnitudes

In Figure 2, we provide an array of quantities that we believe to be key in developing a "feeling for the numbers" associated with human impacts on the Earth system. All of the quantities in Figure 2 are drawn from entries in the database and grouped into the same categories used in the database: land, water, flora and fauna, atmosphere and biogeochemical cycles, and energy (see color scheme at the top of Figure 2). Although the impacts considered here necessarily constitute an incomplete description of human interaction with the planet, these numbers encompass many that are critically important, such as the volume of liquid water resulting from ice melt (Figure 2B), the extent of urban and agricultural land use (Figure 2H), global power consumption (Figure 2N), and the heat uptake and subsequent warming of the ocean surface (Figure 2S). In many cases, the raw numbers are astoundingly large and can therefore be difficult to fathom. Rather than reporting only bare "scientific" units, we present each quantity (when possible) in units that are intended to be relatable as "per capita" values to a broad audience who are members of (or familiar with) typical Western lifestyles. Consider, for example, the 18 TW global power consumption (Figure 2N). For most audiences, it can be difficult to conceptualize what a watt is, let alone the sheer magnitude of a *terawatt*. However, most prospective users of this database likely have a familiarity with the warmth of a 100 W light bulb. With this in mind, we can do a simple conversion to say that the global average power use per person is comparable to constantly running $\approx$ 23 light bulbs per person, making the impact a bit more tangible.

CellPress
OPEN ACCESS

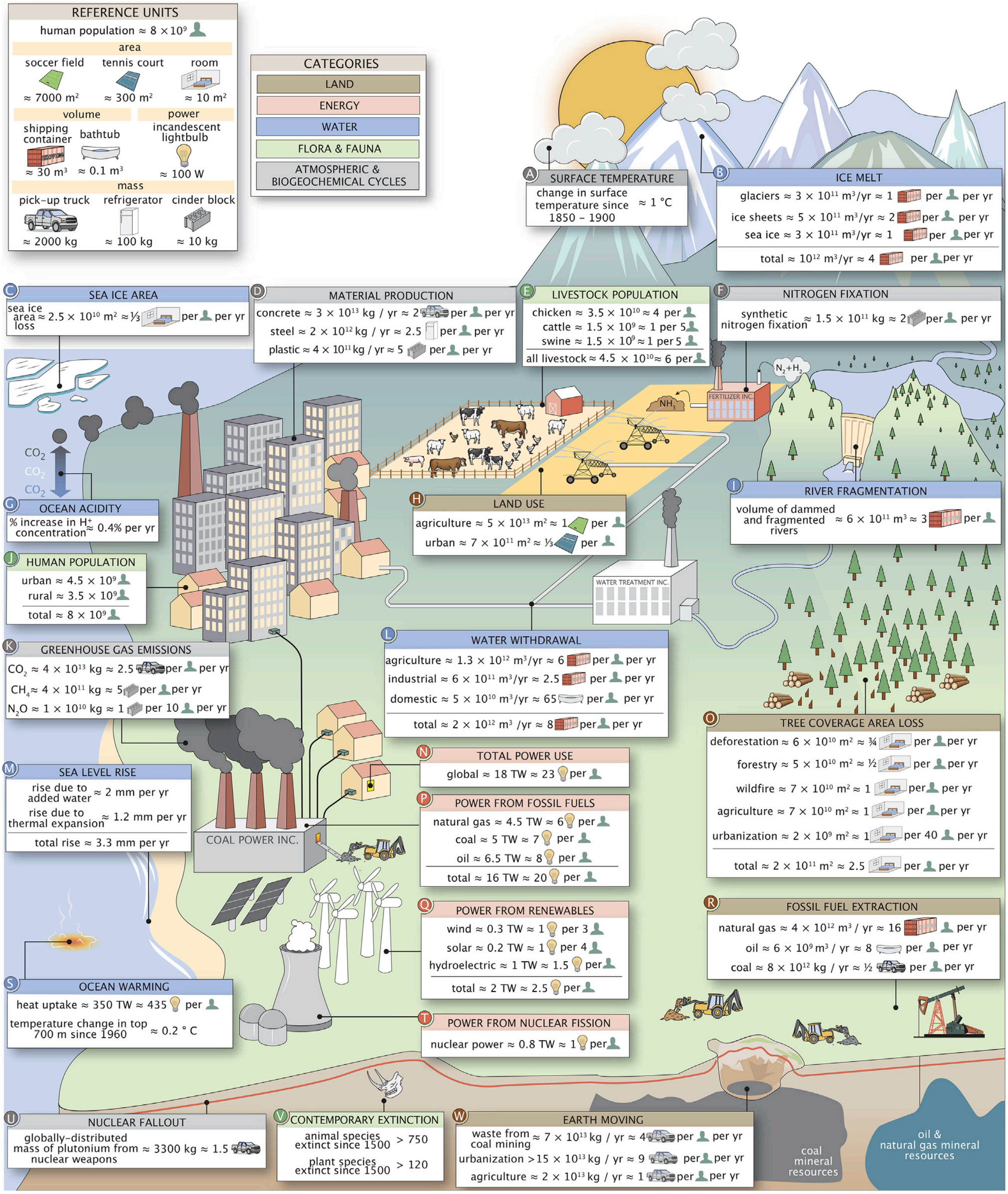

Figure 2. Human impacts on the planet and their relevant magnitudes

Relative units and the broad organizational categories are shown in the top left. Source information and contextual comments for each subpanel are presented in Note S2.

A THE GEOGRAPHY OF HUMAN IMPACTS

Page 1 represents the impact humans have on the Earth at a global scale. While these numbers are handy, it is important to acknowledge that they vary from country-to-country and continent-to-continent. Furthermore, the consequences of these anthropogenic impacts are also unequally distributed, meaning some regions experience effects disproportionate to their contribution. Here, we give a sense of the geographic distribution of several values presented on page 1, broken down by continental region as shown below.

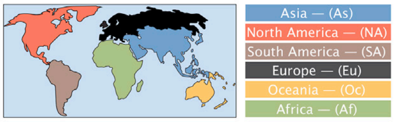

B THE HUMAN POPULATION

There are ≈ 8 billion humans on the planet, with approximately 50% living in 'urban' environments. The majority of both the worlds population (as well as the majority of both urban and rural dwellers) live in Asia.

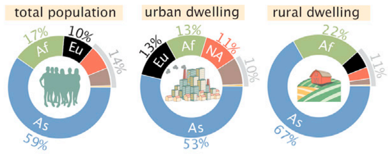

Sources: Food and Agricultural Organization of the United Nations – World Population
Notes: Urban/rural designation has no set definition and follows the conventions set by each reporting country.

C LAND USE

Though humans are nearly evenly split between urban and rural environments, agricultural land is the far more common use of land area. Together, Asia and Africa contain more than half of global agricultural land. Asia alone accomodates more than half of the global urban land area.

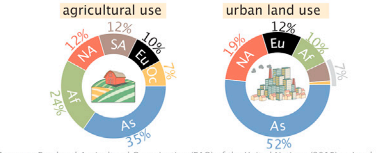

Sources: Food and Agricultural Organization (FAO) of the United Nations (2015) — Land Use [agricultural area]. Florczyk et al. 2019 — GHS Urban Centre Database 2015 [urban land area] Notes: Urban is defined as any inhabited area with ≥ 2500 residents, as defined by the USDA.

D THE LIVESTOCK POPULATION

The global population of terrestrial livestock is around 30 billion individuals, most of which are chickens. Asia houses most of the global livestock population, though South America and Europe harbor more animals on a per-capita basis.

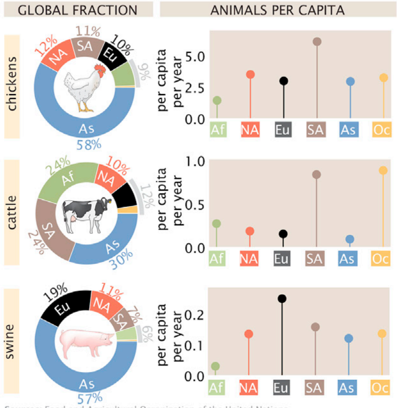

Sources: Food and Agricultural Organization of the United Nations

E WATER WITHDRAWAL

While Asia withdraws the most water for agricultural and municipal needs, North America withdraws the plurality of water for industrial purposes. North America also withdraws more water per capita than any other region.

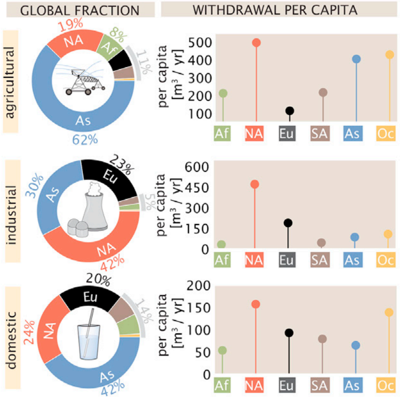

Source: AQUASTAT Main Database, Food and Agriculture Organization of the United Nations. Notes: Values are reported directly from member countries and represent average of 2013–2017 period. Per capita values are computed given population of reporting countries.

F TREE COVERAGE AREA LOSS

Most drivers of tree coverage area loss are comparable in their effect at a global scale. However, there are drastic regional differences in the relative magnitudes.

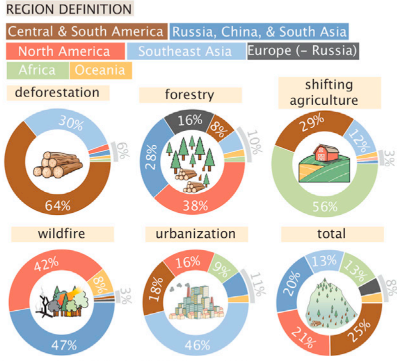

Source: Curtis et al. 2018 doi: 10.1126/science.aau3445.
Notes: Regions are as reported in Curtis et al. 2018. "Deforestation" here denotes permanent removal of tree cover for commodity production. "Shifting agriculture" here denotes forest/shrub land converted to agriculture and later abandoned. All values correspond to breakdown of cumulative tree cover area loss from 2001 – 2015.

G NITROGENOUS FERTILIZER USE & PRODUCTION

Modern agriculture requires nitrogen in amounts beyond what is produced naturally. Asia synthesizes and consumes a large majority of fixed nitrogen. However, Europe and North America dominate per capita synthesis whereas Oceania consumes more fertilizer per capita than any other region.

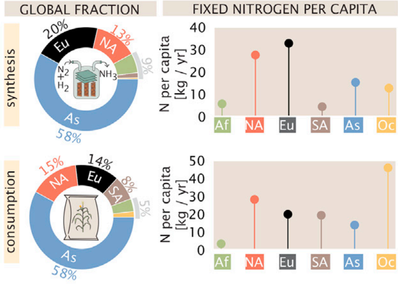

Source: Food and Agricultural Organization (FAO) of the United Nations.
Notes: Values account for reactive nitrogen production/consumption in context of fertilizer only and does not account for plastics, explosives, or other uses.

H GREENHOUSE GAS EMISSIONS

CO_2 and CH_4 are two potent greenhouse gases which are routinely emitted by anthropogenic processes such as burning fuel and rearing livestock. While Asia emits roughly half of all CO_2 and CH_4, North America and Oceania produce the most on a per capita basis, respectively.

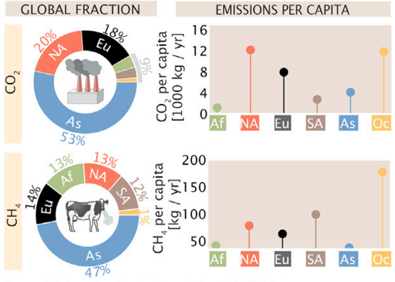

Sources: CO_2 data collated by: Friedlingstein, P. et al. (2019). doi: 10.5194/essd-11-1783-2019. See Panel K on Pg. 4 for complete list of sources. CH_4 data from Saunois et al, 2020 doi: 10.5194/essd-12-1561-2020 Notes: Values report decadal averages in kg CO_2 or CH_4 per year over time period 2008–2017.

I MATERIAL PRODUCTION

Humans excavate an enormous amount of material from the Earth's crust and transform it to build our structures. Two of these materials, concrete and steel, are produced primarily in Asia on both a global and per capita basis. Asia's per capita production of steel is only outpaced by Europe.

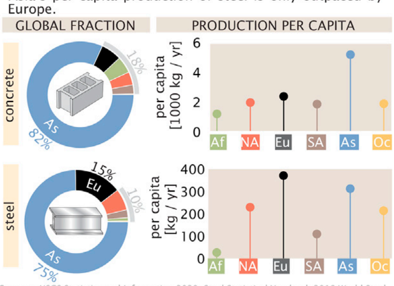

Sources: USGS Statistics and Information 2020, Steel Statistical Yearbook 2019 World Steel Association. Food and Agricultural Organization (FAO) of the United Nations — Annual Population. Notes: Reported values for cement and steel production corresponds to 2017 and 2018 values, respectively. Mass of concrete was calculated using a rule-of-thumb that 1 kg of cement yields 7 kg of concrete (Monteiro et al. 2017. doi: 0.138/nmat4930).

J POWER GENERATION AND CONSUMPTION

From heating water, to powering lights, to moving our vehicles, nearly every facet of modern human life requires the consumption of power, culminating in nearly 20 TW of power use in recent years. Asia consumes over half of the power derived from combustion of fossil fuels, with Europe and North America each consuming around 20% of the global total. Asia also produces the plurality of power from renewable technologies, such as hydroelectric, wind, and solar, however, North America, South America, and Europe each produce more on a per capita basis. Nuclear energy, however, is primarily produced in Europe, with North America and Asia coming in second and third place, respectively. On a per-capita basis, North America consumes or produces more energy than all other regions considered here, yielding a total power consumption of nearly 10,000 W per person.

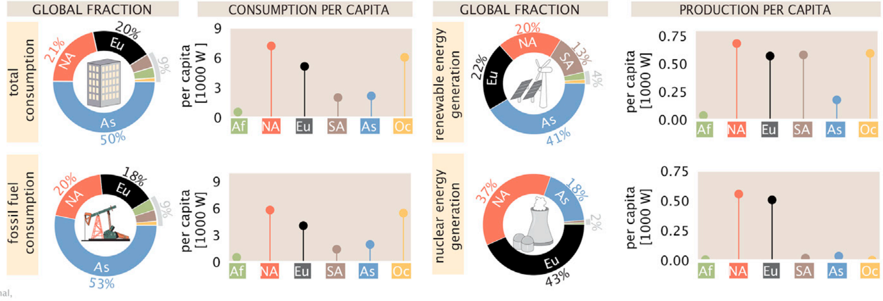

Source: Energy Information Administration of the United States (2017)
Notes: "Renewables" includes hydroelectric, biofuels, biomass (wood), geothermal, wind, and solar. "Fossil fuels" includes coal, oil, and natural gas.

(legend on next page)

Exploring these numbers reveals a number of intriguing quantities and relationships. For example, agriculture repeatedly appears as a major contributor to many human impacts, dominating both global land (Figure 2H) and global water use (Figure 2L) and accounting for approximately a third of global tree cover area loss (Figure 2O). In addition, an enormous mass of nitrogen is synthetically fixed through the Haber-Bosch process, primarilyto produce fertilizer (Figure 2F), which is a major cause of emissions of N_2O (Figure 2K), which is a potent greenhouse gas. About 45 billion livestock are raised on agricultural lands (Figure 2E), which, together with rice paddies, produce a majority of anthropogenic methane emissions (the greenhouse gas CH_4; Figure 2K). On the other hand, urban land area accounts for a very small fraction of land area use ($\approx$ 1%, Figure 2H), and the expansion of cities and suburbs accounts for only $\approx$ 1% of global tree cover area loss (Figure 2O). This is not to say, however, that urban centers are negligible in their global impacts. As urban and suburban areas currently house more than half of the global human population (Figure 2J), many human impacts are linked to industries that directly or indirectly support urban populations' demand for food, housing, travel, electronics, and other goods. For example, the pursuit of urbanization is the dominating factor in the mass of earth moved on an annual basis (Figure 2W).

Collectively, the $\approx$ 8 billion humans on Earth (Figure 2J) consume nearly 20 TW of power, equivalent to 23 100 W light bulbs per person (Figure 2N). Around 80% of this energy derives from the combustion of fossil fuels (Figure 2P). This results in a tremendous mass of CO_2 being emitted annually (Figure 2K), of which only $\approx$ 50% remains in the atmosphere (HuID 70632). A sizable portion of the emissions are absorbed by the oceans (HuID 99089), leading to a steady increase in ocean acidity (Figure 2G) and posing risks to marine ecosystems.[39] Furthermore, increasing average global temperatures, primarily caused by greenhouse gas emissions, contribute to sea-level rise not only in the form of added water from melting ice (Figure 2B and 2M), but also due to thermal expansion of ocean water (Figure 2M), which accounts for $\approx$ 30% of observed sea-level rise.[40] These are just a few ways in which one can traverse the impacts illustrated in Figure 2, revealing the remarkable extent to which these impacts are interconnected. We encourage the reader to explore this figure in a similar manner, blazing their own trail through the values.

Regional distribution

While Figure 2 presents the magnitude of human impacts at a global scale, it is important to recognize that these impacts—both their origins and their repercussions—are variable across the globe. That is, different societies vary in their preferences for food (e.g., Americans consume relatively little fish) and modes of living (e.g., apartments versus houses), have different levels of economic development (e.g., Canada compared with Malaysia), rely on different natural resources to build infrastructure (e.g., wood versus concrete) and generate power (e.g., nuclear versus coal), and promote different extractive or polluting industries (e.g., lithium mining versus palm oil farming). Some of these regional differences are evident in Figure 3, which summarizes regional breakdowns of several drivers of global human impacts, e.g., livestock populations and greenhouse gas emissions.

Just as impactful human activities like coal power generation and swine farming are more common in some regions than others (Figure 2), the impacts of human activities affect some regions more than others.[42] Figure 3 displays a coarse regional breakdown of the numbers from Figure 2 for which regional distributions could be determined from the literature. The region definitions used in Figure 3 are similar to the definitions set forth by the Food and Agricultural Organization (FAO) of the United Nations, assigning the semi-continental regions of North America, South America, Africa, Europe (including Russia), Asia, and Oceania. Here, we specify both the total contribution of each region and the per-capita value, given the population of that region as of the year(s) in which the quantity was measured.

Much as in the case of Figure 2, interesting details emerge from Figure 3. For example, Asia dominates global agricultural water withdrawal (excluding natural watering via rainfall), using about 62% of the total, while North America takes the lead in industrial water withdrawal. Interestingly, on a per-capita basis, North America withdraws the most water for all uses: agricultural, industrial, and domestic.

North America also emits more CO_2 per capita than any other region, with Oceania and Europe coming second and third, respectively. This disparity can be partially understood by considering the regional distribution of fossil fuel consumption, the dominant source of CO_2 emissions (Figure 3J). While Asia consumes more than half of the total fossil fuel energy, per-capita consumption is markedly lower than in North America, Europe, and Oceania (Figure 3J). Interestingly, the story is different when it comes to methane. Oceania and South America are the largest emitters of anthropogenic CH_4, mainly due to a standing population of cattle that rivals that of humans in those regions (Figure 3D) and produces this potent greenhouse gas through enteric fermentation.[33] Regional disparities are also apparent in the means of energy production. While consuming only 4% of the total power, South America generates about 14% of the renewable energy. Nuclear power generation, on the other hand, is dominated by North America and Europe, while Oceania, which has a single research-grade nuclear reactor, generates nearly zero nuclear energy.

Investigating the causes of forest loss by geographic region likewise highlights interesting differences. At a global level, all drivers of forest loss are comparable in magnitude, except for urbanization, which accounts for $\approx$ 1% of total annual tree cover area loss (Figure 2O). Despite comparable magnitudes, different drivers of forest loss have different long-term consequences.[30] Forest loss due to wildfires and forestry often result in regrowth, while commodity-driven harvesting and urbanization tend to be drivers of long-lasting deforestation.[43,44] Central and South America account for about 65% of commodity-driven deforestation

Figure 3. Regional distribution of anthropogenic effects

(A) Several quantities from Figure 2 were selected, and the relative magnitudes were broken down by subcontinental area.

(B–J) Donut charts in all sections show the relative contributions of each quantity by region. Ball-and-stick plots show the per-capita breakdown of each quantity across geographic regions. All data for global and per-capita breakdowns correspond to the latest year for which data were available. The regional breakdown for deforestation uses the regional convention as reported in the source data.[41]

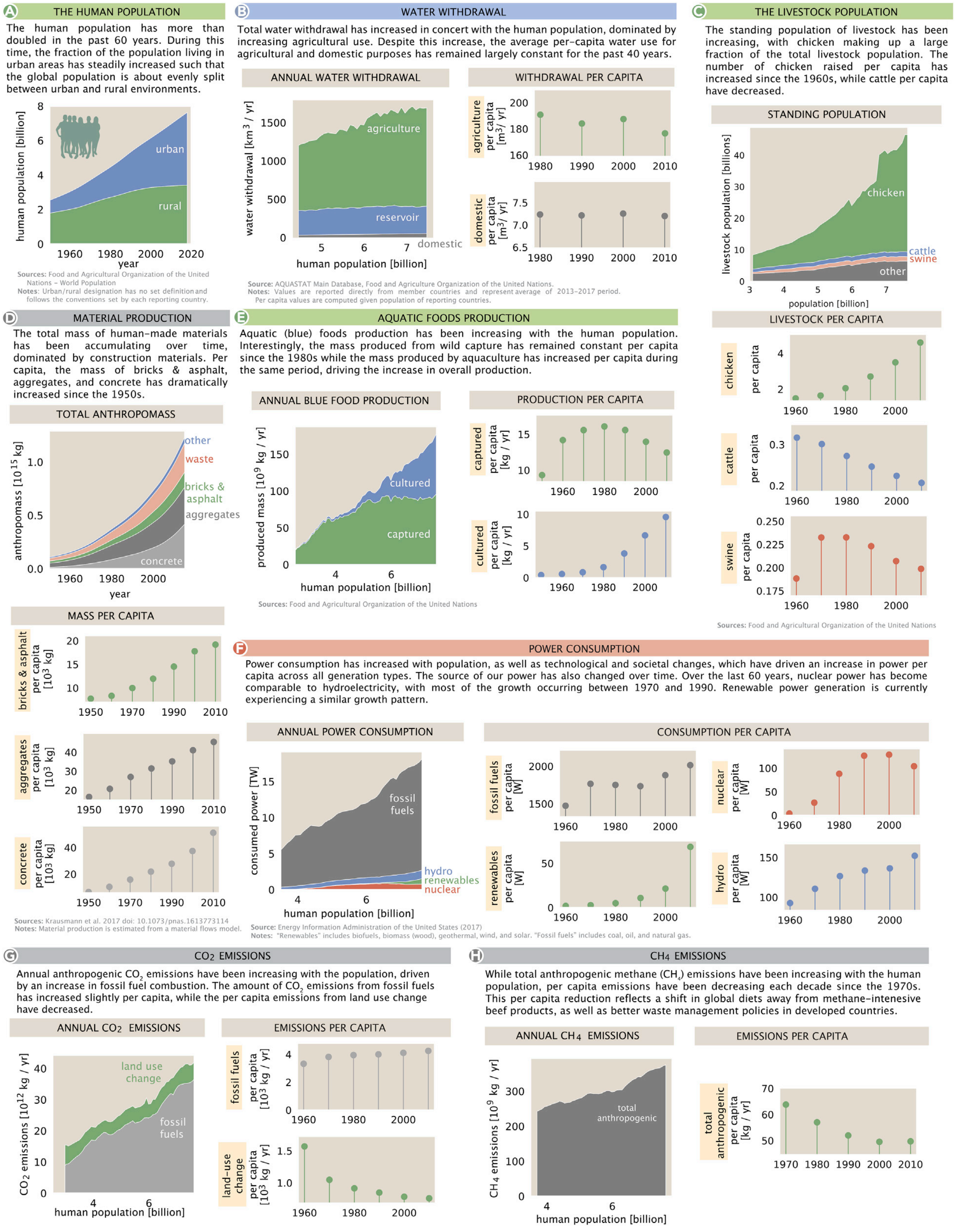

(legend on next page)

(legend on next page)

(meaning clear-cutting and human-induced fires with no substantial regrowth of tree cover), whereas a majority of forest loss due to shifting agriculture occurs in Africa (where regrowth does occur). Together, wildfires in North America, Russia, China, and South Asia make up nearly 90% of losses due to fire.[41] While urbanization is the smallest driver of tree cover loss globally, it can still have strong impacts at the regional level, perturbing local ecosystems and biodiversity.[45,46]

Time series

When available, the Human Impacts Database includes time-series data for each quantity. Just as the regional distributions of impactful human activities help us understand differences between societies and regions, studying the history of these activities highlights recent technological and economic developments that intensify or reduce their impacts. When considering the history of human impacts on the Earth, it is natural to start by considering the growth of the human population over time. As shown in Figure 4, the global human population grew nearly continually over the past 80 years, with the current population nearing 8 billion. Historically, most of the global human population lived in rural areas (about 70% as of 1950, HuID 93995). Recent decades have been marked by a substantial shift in how humans live globally, with around half of the human population now living in urban or suburban settings ($\approx$ 55%, HuID 93995).

Given the growth of the human population, it is reasonable to consider that human population may be the most natural scale to measure human impacts.[47] To assess this possibility, we plotted per-capita impacts over several decades (Figure 4). If impacts are growing in direct proportion to the human population, per-capita impacts would be constant over time. Indeed, this is roughly true for per-capita water withdrawals over the past 40 years (Figure 4B). Deviations from proportionality may indicate important changes in human activities. For example, in recent decades, per-capita chicken populations grew by nearly 2-fold, while per-capita cattle populations shrunk by roughly 25%, reflecting a modest transition away from beef and toward chicken as a source of animal protein in global diets (HuIDs 40696 and 79776).

One very visible impact accompanying the shift of the human population to urban environments is the increase in production of anthropogenic mass: materials such as concrete, steel, lumber, and plastics used to build roads, buildings, machines, packaging, and other useful human-made items. Since these materials are degraded very slowly, anthropogenic mass has been accumulating over time. In addition, the mass of concrete, aggregates like asphalt, and bricks per capita has been increasing since the 1950s (Figure 4D). Concrete, in particular, has increased from less than 10 tons per person in the 1950s to almost 30 tons per person in the 2010s. This increase in per-capita anthropogenic mass means that the increase in production of these materials is outpacing the growth of the human population.

These material production trends have been enabled, in part, by a sustained increase in power generation. As evident from Figure 4, total power consumption has been increasing roughly proportionally with the human population. Per-capita consumption has also increased across all generation types, including fossil fuels, hydropower, nuclear, and renewables. The growth among nuclear and renewables has been especially dramatic, and nuclear power now roughly equals hydropower production. Production of crops, aquaculture, and populations of livestock are all likewise correlated with growth in the human population (Figures 4C and 4E). The total number of livestock has increased with the human population, primarily due to increasing chicken populations as discussed above. The dominant means of aquatic food production has also shifted over this time: until roughly 1980, nearly all seafood was captured wild, but since then aquaculture has grown to account for roughly ½ of aquatic food production (HuID 61233, Figure 4E).

Turning our focus to greenhouse gases, we see that annual anthropogenic CO_2 emissions have been increasing with the population (Figure 4G). Burning of fossil fuels is the dominant contributor to anthropogenic emissions and has increased slightly on a per-capita basis over the past 60 years. In contrast, as the pace of global deforestation has slowed,[48,49] emissions of CO_2 due to land-use change have decreased per capita. These two trends roughly neutralize each other, leading to little overall change in CO_2 emissions per capita since the 1960s. Akin to CO_2 emissions due to land-use change, CH_4 emissions show a sub-linear trend with human population, partially due to a decline in ruminant livestock per capita (Figures 4C and 4H).

DISCUSSION

Quantitative literacy is necessary for "understanding" in nearly all branches of science. As our collective knowledge of anthropogenic impacts expands, it has become challenging to sift through the literature to collect specific numbers useful for both calculation and communication. We have attempted to reduce this barrier to entry on several fronts. We have canvassed the scientific literature, governmental, industrial, and international reports to assemble a broad, quantitative picture of how human activities have affected the Earth's atmosphere, oceans, rivers, lands, biota, chemistry, and geology. In doing so, we have created an online, searchable database housing an array of quantities and data that describe different facets of the human-Earth interface. We view this database as an accessory, rather than a replacement, for the myriad scientific databases that exist and are publicly available on the internet (some of which are listed on the database website www.anthroponumbers.org/catalog/databases). While these databases are invaluable resources for accessing scientific data, the Human Impacts Database is built from the ground up with the intention of being broadly accessible to scientists and the curious general public alike to help build the collective quantitative literacy of the Anthropocene. Beyond the database, we have assembled

Figure 4. Temporal dynamics of key human impacts

(A) Several quantities from Figure 2 were selected, and the magnitudes were plotted as a function of either time (for cumulative quantities such as anthropomass) or human population.

(B–H) Ball-and-stick plots show the per-capita breakdown as decadal averages to give a more reflective view of cultural and technological shifts than year-to-year variation.

these data into a comprehensive snapshot, released alongside this writing as a standalone graphical document (Data S1), with all underlying data, associated uncertainties, and referencing housed in the Human Impacts Database. While necessarily incomplete, these resources provide a broad view of the ways in which human activities are having an impact on the Earth on multiple fronts.

One insight that emerges from a holistic consideration of these diverse human activities together is that they are deeply intertwined and driven by a small number of pivotal factors: the size of the human population, the composition of our diets, and our demand for materials and energy to build and power our increasingly complex and mechanized societies. Understanding the scale of human agriculture and water and power usage provides a framework for understanding most of the numerical gallery presented in Figure 2. Perhaps unsurprisingly, we find that feeding the growing human population is a major driver of a large swath of human impacts on Earth, dominating global land (Figure 2H, HuID 29582) and water use (Figure 2L; HuIDs 84545, 43593, 95345), as well as significantly contributing to tree cover loss (Figure 2O, HuID 24388), earth moving (Figure 2W; HuIDs 19415, 41496), and anthropogenic nitrogen fixation (Figure 2F; HuIDs 60580, 61614), to name a few such examples. The Human Impacts Database provides a resource to explore relationships between values temporally, globally, and locally, and go beyond the standalone values often reported in isolation or cast solely through the lenses of impact, population, affluence, and technology (I = PAT) relationships.

It is common in this setting to argue that the bewildering breadth and scale of human impacts should motivate some specific remediation at the global or local scale. We, instead, take a more modest "just the facts" approach. The numbers presented here show that human activities affect our planet to a large degree in many different and incommensurate ways, but they do not provide a roadmap for the future. Rather, we contend that any plans for the future should be made in the light of a comprehensive and quantitative understanding of the interconnected ways in which human activities impact the Earth system globally (Figure 2), locally (Figure 3), and temporally (Figure 4). Achieving such an understanding will require the synthesis of a broad literature across many disciplines. While the quantities we have chosen to explore are certainly not exhaustive, they represent some of the key axes that frequently drive scientific and public discourse and shape policy across the globe.

Earth is the only habitable planet we know of, so it is crucial to understand how we got here and where we are going. That is, how (and why) have human impacts changed over time? How are they expected to change in the future? For every aspect of human entanglement with the Earth system—from water use to land use, greenhouse gas emissions, mining of precious minerals, and so on—there are excellent studies measuring impacts and predicting their future trajectories. Of particular note are the data-rich and explanatory reports from the Intergovernmental Panel on Climate Change[50,51] and the efforts toward defining "planetary boundaries."[52] We hope that the Human Impacts Database and the associated resources with this work provide a reference to explore the human-induced interdependencies between many axes of the human-Earth system and will engage the scientific community, ultimately helping humanity coexist stably with the only planet we have.

EXPERIMENTAL PROCEDURES

Resource availability

Lead contact

Requests for further information should be directed to and will be fulfilled by the lead contact, Griffin Chure (griffinchure@gmail.com).

Materials availability

No materials were used in the generation of this work, other than the code and data as described below. We have collated all data shown in Figures 2–4, along with all information in Note S2 as a printable, "graphical snapshot" (Data S1).

Data and code availability

For every dataset included in the database, there is a folder in the GitHub repository https://github.com/rpgroup-pboc/human_impacts (DOI: 10.5281/zenodo.4453276) that includes the source data, the processed data, and the code to generate the "tidy" data from the source data. Each folder also includes a README file that includes information about the dataset. In addition, all of the code used to generate the figures can be found in the GitHub repository under the "figures" folder. We strongly encourage the scientific community to fork this repository, submit pull requests, and open new constructive issues through the GitHub repository interface.

The database and the FAIR principles of data reuse

The primary goal of the Human Impacts Database is to provide a resource for the rapid discovery quantities related to the human-Earth system while minimizing the grunt work needed to access (and understand) the underlying data. This means that facilitating data reuse and reproducibility of any analyses is paramount to the importance of the database. To that end, we abide by the *FAIR Guiding Principles for Scientific Data Management and Stewardship* (www.go-fair.org/fair-principles/). These principles are guidelines to maximize the findability, accessibility, interoperability, and reusability of original scientific data. The database closely follows these principles, as is briefly outlined below:

- Findability: The underlying data can be easily searched and navigated, permitting rapid discovery. Individual entries are assigned a unique integer identifier that serves as a permanent referencing tool and are provided with rich metadata about the method of determination, original source, data use protection policy, and quantitative value in diverse units.
- Accessibility: The original source of the underlying data is always reported hyperlinked when legally permissible. The transformation, collation, or manipulation of the underlying data that was necessary to add it to the Human Impacts Database is preserved under a publicly accessible, version-controlled, GitHub repository (github.com/rpgroup-pboc/human_impacts) and is permanently accessible via https://doi.org/10.5281/zenodo.4453276. This protects against permanent loss of the data even if an entry is deleted from the database.
- Interoperability: The data are provided in a human readable format with an emphasis on description of the data and their source. The vast majority of datasets are transformed programmatically to follow a "tidy," long-form format that facilitates computational analysis of the data. As the values are hand curated and the target audience is a curious human, we have not developed an API for programmatic access of the database, and do not have plans to do so in the foreseeable future.
- Reusability: All entries in the database and the corresponding GitHub repository are extensively annotated with rich metadata, preventing the need for guesswork as to how the data were collected or what the column names refer to in the original or processed data. Furthermore, all data held in the database and repository follow the legal guidelines as presented by their original owner. This licensing is directly linked to in each entry.

SUPPLEMENTAL INFORMATION

Supplemental information can be found online at https://doi.org/10.1016/j.patter.2022.100552.

CellPress
OPEN ACCESS

ACKNOWLEDGMENTS

We are incredibly grateful for the generosity of a wide array of experts for their advice, suggestions, and criticism of this work. Specifically, we thank Suzy Beeler, Joseph Berry, Lars Bildsten, Justin Bois, Chris Bowler, Matthew Burgess, Ken Caldeira, Jörn Callies, Sean B. Carroll, Ibrahim Cissé, Joel Cohen, Michelle Dan, Bethany Ehlmann, Gidon Eshel, Moi Exposito-Alonso, Paul Falkowski, Daniel Fisher, Thomas Frederikse, Neil Fromer, Eric Galbraith, Lea Goentoro, Evan Groover, John Grotzinger, Soichi Hirokawa, Greg Huber, Christina Hueschen, Bob Jaffe, Elizabeth Kolbert, Thomas Lecuit, Raphael Magarik, Jeff Marlow, Brad Marston, Jitu Mayor, Elliot Meyerowitz, Lisa Miller, Dianne Newman, Luke Oltrogge, Nigel Orme, Victoria Orphan, Marco Pasti, Pietro Perona, Noam Prywes, Stephen Quake, Hamza Raniwala, Manuel Razo-Mejia, Thomas Rosenbaum, Benjamin Rubin, Alex Rubinsteyn, Shyam Saladi, Tapio Schneider, Murali Sharma, Alon Shepon, Arthur Smith, Matthieu Talpe, Wati Taylor, Julie Theriot, Tadashi Tokieda, Cat Triandifillou, Sabah Ul-Hasan, Tine Valencic, Ned Wingreen, and Emily Zakem. We also thank Yue Qin for sharing data related to global water consumption. Many of the topics in this work began during the Applied Physics 150C course taught at Caltech during the early days of the COVID-19 pandemic. This work was supported by the Resnick Sustainability Institute at Caltech and the Schwartz-Reisman Collaborative Science Program at the Weizmann Institute of Science. G.C. acknowledges support by the NSF Postdoctoral Research Fellowships in Biology Program (grant no. 2010807).

AUTHOR CONTRIBUTIONS

Conceptualization, G.C., R.A.B., R.M., and R.P.; investigation, G.C., R.A.B., A.I.F., N.S.S., M.K., I.L.G., and Y.M.B.; data curation, G.C., R.A.B., N.S.S., M.K., and I.L.G.; software, G.C.; writing – original draft, G.C., R.A.B., A.I.F., N.S.S., I.L.G., R.M., and R.P.; writing – review & editing, G.C., R.A.B., and R.P.; visualization, G.C. and R.A.B.; project administration, G.C., R.A.B., and R.P.

DECLARATION OF INTERESTS

The authors declare no competing interests.

Received: March 4, 2022
Revised: May 26, 2022
Accepted: June 23, 2022
Published: August 3, 2022

WEB RESOURCES

BioNumbers Database, https://bionumbers.hms.harvard.edu
Human Impacts Database, http://anthroponumbers.org

REFERENCES

1. Fischer, W.W., Hemp, J., and Johnson, J.E. (2016). Evolution of oxygenic photosynthesis. Annu. Rev. Earth Planet Sci. *44*, 647–683. https://doi.org/10.1146/annurev-earth-060313-054810.
2. Hodgskiss, M.S.W., Crockford, P.W., Peng, Y., Wing, B.A., and Horner, T.J. (2019). A productivity collapse to end Earth's Great Oxidation. Proc. Natl. Acad. Sci. USA *116*, 17207–17212. https://doi.org/10.1073/pnas.1900325116.
3. Gumsley, A.P., Chamberlain, K.R., Bleeker, W., Söderlund, U., de Kock, M.O., Larsson, E.R., and Bekker, A. (2017). Timing and tempo of the great oxidation event. Proc. Natl. Acad. Sci. USA *114*, 1811–1816. https://doi.org/10.1073/pnas.1608824114.
4. Sessions, A.L., Doughty, D.M., Welander, P.V., Summons, R.E., and Newman, D.K. (2009). The continuing puzzle of the great oxidation event. Curr. Biol. *19*, R567–R574. https://doi.org/10.1016/j.cub.2009.05.054.
5. Estes, J.A., Terborgh, J., Brashares, J.S., Power, M.E., Berger, J., Bond, W.J., Carpenter, S.R., Essington, T.E., Holt, R.D., Jackson, J.B.C., et al. (2011). Trophic downgrading of planet earth. Science *333*, 301–306. https://doi.org/10.1126/science.1205106.
6. Springer, A.M., Estes, J.A., van Vliet, G.B., Williams, T.M., Doak, D.F., Danner, E.M., Forney, K.A., and Pfister, B. (2003). Sequential megafaunal collapse in the North Pacific Ocean: an ongoing legacy of industrial whaling? Proc. Natl. Acad. Sci. USA *100*, 12223–12228. https://doi.org/10.1073/pnas.1635156100.
7. Hale, S.L., and Koprowski, J.L. (2018). Ecosystem-level effects of keystone species reintroduction: a literature review: effects of keystone species reintroduction. Restor. Ecol. *26*, 439–445. https://doi.org/10.1111/rec.12684.
8. Holdo, R.M., Sinclair, A.R.E., Dobson, A.P., Metzger, K.L., Bolker, B.M., Ritchie, M.E., et al. (2009). A disease-mediated trophic cascade in the Serengeti and its implications for ecosystem. PLoS Biol. *7*, e1000210. https://doi.org/10.1371/journal.pbio.1000210.
9. Ceballos, G., Ehrlich, P.R., Barnosky, A.D., García, A., Pringle, R.M., and Palmer, T.M. (2015). Accelerated modern human–induced species losses: entering the sixth mass extinction. Sci. Adv. *1*, e1400253. https://doi.org/10.1126/sciadv.1400253.
10. Davidson, A.D., Hamilton, M.J., Boyer, A.G., Brown, J.H., and Ceballos, G. (2009). Multiple ecological pathways to extinction in mammals. Proc. Natl. Acad. Sci. USA *106*, 10702–10705. https://doi.org/10.1073/pnas.0901956106.
11. Fortin, D., Beyer, H.L., Boyce, M.S., Smith, D.W., Duchesne, T., and Mao, J.S. (2005). Wolves influence Elk movements: Behavior shapes a trophic cascade in Yellowstone National Park. Ecology *86*, 1320–1330. https://doi.org/10.1890/04-0953.
12. Tronstad, L.M., Hall, R.O., Koel, T.M., and Gerow, K.G. (2010). Introduced lake trout produced a four-level trophic cascade in Yellowstone Lake. Trans. Am. Fish. Soc. *139*, 1536–1550. https://doi.org/10.1577/t09-151.1.
13. Palumbi, S.R. (2001). Humans as the world's greatest evolutionary force. Science *293*, 1786–1790. https://doi.org/10.1126/science.293.5536.1786.
14. Balmaseda, M.A., Trenberth, K.E., and Källén, E. (2013). Distinctive climate signals in reanalysis of global ocean heat content. Geophys. Res. Lett. *40*, 1754–1759. https://doi.org/10.1002/grl.50382.
15. Cheng, L., Trenberth, K.E., Fasullo, J., Boyer, T., Abraham, J., and Zhu, J. (2017). Improved estimates of ocean heat content from 1960 to 2015. Sci. Adv. *3*, e1601545. https://doi.org/10.1126/sciadv.1601545.
16. Bar-On, Y.M., Phillips, R., and Milo, R. (2018). The biomass distribution on Earth. Proc. Natl. Acad. Sci. USA *115*, 6506–6511. https://doi.org/10.1073/pnas.1711842115.
17. Elhacham, E., Ben-Uri, L., Grozovski, J., Bar-On, Y.M., and Milo, R. (2020). Global human-made mass exceeds all living biomass. Nature *588*, 442–444. https://doi.org/10.1038/s41586-020-3010-5.
18. IPCC (2019). The Ocean and Cryosphere in a Changing Climate.
19. Peng, H., Ke, C., Shen, X., Li, M., and Shao, Z. (2020). Summer albedo variations in the Arctic Sea ice region from 1982 to 2015. Int. J. Climatol. *40*, 3008–3020. https://doi.org/10.1002/joc.6379.
20. Marcianesi, F., Aulicino, G., and Wadhams, P. (2021). Arctic sea ice and snow cover albedo variability and trends during the last three decades. Polar Sci *28*, 100617. https://doi.org/10.1016/j.polar.2020.100617.
21. Mouginot, J., Rignot, E., Bjørk, A.A., van den Broeke, M., Millan, R., Morlighem, M., Noël, B., Scheuchl, B., and Wood, M. (2019). Forty-six years of Greenland Ice Sheet mass balance from 1972 to 2018. Proc. Natl. Acad. Sci. USA *116*, 9239–9244. https://doi.org/10.1073/pnas.1904242116.
22. MacGregor, J.A., Fahnestock, M.A., Catania, G.A., Aschwanden, A., Clow, G.D., Colgan, W.T., Gogineni, S.P., Morlighem, M., Nowicki, S.M.J., Paden, J.D., et al. (2016). A synthesis of the basal thermal state of the Greenland Ice Sheet. J. Geophys. Res. Earth Surf. *121*, 1328–1350. https://doi.org/10.1002/2015jf003803.
23. Kwok, R. (2018). Arctic sea ice thickness, volume, and multiyear ice coverage: losses and coupled variability (1958–2018). Environ. Res. Lett. *13*, 105005. https://doi.org/10.1088/1748-9326/aae3ec.
24. Massom, R.A., Scambos, T.A., Bennetts, L.G., Reid, P., Squire, V.A., and Stammerjohn, S.E. (2018). Antarctic ice shelf disintegration triggered by

sea ice loss and ocean swell. Nature *558*, 383–389. https://doi.org/10.1038/s41586-018-0212-1.

25. Notz, D., and Stroeve, J. (2016). Observed Arctic sea-ice loss directly follows anthropogenic CO_2 emission. Science *354*, 747–750. https://doi.org/10.1126/science.aag2345.
26. Stroeve, J., and Notz, D. (2018). Changing state of Arctic sea ice across all seasons. Environ. Res. Lett. *13*, 103001. https://doi.org/10.1088/1748-9326/aade56.
27. Goode, P.R., Pallé, E., Shoumko, A., Shoumko, S., Montañes-Rodriguez, P., and Koonin, S.E. (2021). Earth's albedo 1998–2017 as measured from earthshine. Geophys. Res. Lett. *48*. https://doi.org/10.1029/2021gl094888.
28. Friedlingstein, P., Jones, M.W., O'sullivan, M., Andrew, R.M., Hauck, J., Peters, G.P., Peters, W., Pongratz, J., Sitch, S., Le Quéré, C., et al. (2019). Global carbon budget 2019. Earth Syst. Sci. Data *11*, 1783–1838.
29. Houghton, R.A., and Nassikas, A.A. (2017). Global and regional fluxes of carbon from land use and land cover change 1850-2015: Carbon emissions from land use. Glob. Biogeochem. Cycles *31*, 456–472. https://doi.org/10.1002/2016gb005546.
30. Hansis, E., Davis, S.J., and Pongratz, J. (2015). Relevance of methodological choices for accounting of land use change carbon fluxes. Glob. Biogeochem. Cycles *29*, 1230–1246. https://doi.org/10.1002/2014gb004997.
31. Tian, H., Xu, R., Canadell, J.G., Thompson, R.L., Winiwarter, W., Suntharalingam, P., Davidson, E.A., Ciais, P., Jackson, R.B., Janssens-Maenhout, G., et al. (2020). A comprehensive quantification of global nitrous oxide sources and sinks. Nature *586*, 248–256. https://doi.org/10.1038/s41586-020-2780-0.
32. Keeling, C.D. (1960). The concentration and isotopic abundances of carbon dioxide in the atmosphere. Tellus *12*, 200–203. https://doi.org/10.1111/j.2153-3490.1960.tb01300.x.
33. Saunois, M., Stavert, A.R., Poulter, B., Bousquet, P., Canadell, J.G., Jackson, R.B., Raymond, P.A., Dlugokencky, E.J., Houweling, S., Patra, P.K., et al. (2020). The global methane budget 2000–2017. Earth Syst. Sci. Data *12*, 1561–1623.
34. Yoon, J.-H., Kravitz, B., Rasch, P.J., Simon Wang, S.Y., Gillies, R.R., and Hipps, L. (2015). Extreme fire season in California: A glimpse into the future? Bull. Am. Meteorol. Soc. *96*, S5–S9. S9. https://doi.org/10.1175/bams-d-15-00114.1.
35. Seager, R., Hoerling, M., Schubert, S., Wang, H., Lyon, B., Kumar, A., Nakamura, J., and Henderson, N. (2015). Causes of the 2011–14 California drought. J. Clim. *28*, 6997–7024. https://doi.org/10.1175/jcli-d-14-00860.1.
36. Milo, R., Jorgensen, P., Moran, U., Weber, G., and Springer, M. (2010). BioNumbers—the database of key numbers in molecular and cell biology. Nucleic Acids Res. *38*, D750–D753. https://doi.org/10.1093/nar/gkp889.
37. Milo, R., and Phillips, R. (2016). Cell Biology by the Numbers (Garland Science).
38. Wickham, H. (2014). Tidy data. J. Stat. Softw. *59*. https://doi.org/10.18637/jss.v059.i10.
39. Andersson, A., Kline, D., Edmunds, P., Archer, S., Bednaršek, N., Carpenter, R., Chadsey, M., Goldstein, P., Grottoli, A., Hurst, T., et al. (2015). Understanding ocean acidification impacts on organismal to ecological scales. Oceanography *25*, 16–27. https://doi.org/10.5670/oceanog.2015.27.
40. Frederikse, T., Landerer, F., Caron, L., Adhikari, S., Parkes, D., Humphrey, V.W., Dangendorf, S., Hogarth, P., Zanna, L., Cheng, L., and Wu, Y.H. (2020). The causes of sea-level rise since 1900. Nature *584*, 393–397. https://doi.org/10.1038/s41586-020-2591-3.
41. Curtis, P.G., Slay, C.M., Harris, N.L., Tyukavina, A., and Hansen, M.C. (2018). Classifying drivers of global forest loss. Science *361*, 1108–1111. https://doi.org/10.1126/science.aau3445.
42. Patz, J.A., Campbell-Lendrum, D., Holloway, T., and Foley, J.A. (2005). Impact of regional climate change on human health. Nature *438*, 310–317. https://doi.org/10.1038/nature04188.
43. Bowman, D.M.J.S., Balch, J.K., Artaxo, P., Bond, W.J., Carlson, J.M., Cochrane, M.A., D'Antonio, C.M., DeFries, R.S., Doyle, J.C., Harrison, S.P., et al. (2009). Fire in the earth system. Science *324*, 481–484. https://doi.org/10.1126/science.1163886.
44. Santín, C., Doerr, S.H., Preston, C.M., and González-Rodríguez, G. (2015). Pyrogenic organic matter production from wildfires: A missing sink in the global carbon cycle. Glob. Change Biol. *21*, 1621–1633. https://doi.org/10.1111/gcb.12800.
45. Olivier, T., Thébault, E., Elias, M., Fontaine, B., and Fontaine, C. (2020). Urbanization and agricultural intensification destabilize animal communities differently than diversity loss. Nat. Commun. *11*, 2686. https://doi.org/10.1038/s41467-020-16240-6.
46. Newbold, T., Hudson, L.N., Hill, S.L.L., Contu, S., Lysenko, I., Senior, R.A., Börger, L., Bennett, D.J., Choimes, A., Collen, B., et al. (2015). Global effects of land use on local terrestrial biodiversity. Nature *520*, 45–50. https://doi.org/10.1038/nature14324.
47. Syvitski, J., Waters, C.N., Day, J., Milliman, J.D., Summerhayes, C., Steffen, W., Zalasiewicz, J., Cearreta, A., Gałuszka, A., Hajdas, I., et al. (2020). Extraordinary human energy consumption and resultant geological impacts beginning around 1950 CE initiated the proposed Anthropocene Epoch. Commun. Earth Environ. *1*, 32. https://doi.org/10.1038/s43247-020-00029-y.
48. FAO (2020). State of the World's Forests 2020.
49. Minx, J.C., Lamb, W.F., Andrew, R.M., Canadell, J.G., Crippa, M., Döbbeling, N., Forster, P.M., Guizzardi, D., Olivier, J., Peters, G.P., et al. (2021). A comprehensive and synthetic dataset for global, regional, and national greenhouse gas emissions by sector 1970–2018 with an extension to 2019. Earth Syst. Sci. Data *13*, 5213–5252. https://doi.org/10.5194/essd-13-5213-2021.
50. Masson-Dellmotte, V., Zhai, P., Pörtner, H.O., Roberts, D., Skea, J., Shukla, P.R., et al. (2018). In Special Report: Global Warming of 1.5 oC (IPCC).
51. Pörtner, H.-O., Roberts, D.C., Masson-Delmotte, V., and Zhai, P., eds. (2019). IPCC Special Report on the Ocean and Cryosphere in a Changing Climate (IPCC).
52. Steffen, W., Richardson, K., Rockström, J., Cornell, S.E., Fetzer, I., Bennett, E.M., Biggs, R., Carpenter, S.R., de Vries, W., de Wit, C.A., et al. (2015). Sustainability. Planetary boundaries: guiding human development on a changing planet. Science *347*, 1259855. https://doi.org/10.1126/science.1259855.

Patterns, Volume *3*

Supplemental information

Anthroponumbers.org: A quantitative database of human impacts on Planet Earth

Griffin Chure, Rachel A. Banks, Avi I. Flamholz, Nicholas S. Sarai, Mason Kamb, Ignacio Lopez-Gomez, Yinon Bar-On, Ron Milo, and Rob Phillips

Supplemental Note 1: Selection, Validation, and Curation of Values

The breadth of measurements of human impacts on the planet is enormous, covering a wide array of disciplines and methods. While this is a boon for science, this imposes a very important burden – any value we care to enter into the Human Impacts database must be carefully examined and deemed credible and appropriate for the database. While we certainly acknowledge we are not domain experts in *all* of these fields, the members of the administrative team span a broad range of backgrounds, and are all quantitative scientists who both deeply value the utility of quantitative measurements and have the domain expertise to assess whether the reported values make sense and are determined with trustworthy methods. In this section, we briefly outline the general procedure undertaken before a value is entered into the Human Impacts Database.

Identifying a Potential Entry

Our first action is identifying a value or set of values and determining whether they are pertinent to Human Impacts. We take a broad definition of "Human Impacts", but enforce that any value must be either (i) a direct result of anthropogenic action, (ii) contributed to by anthropogenic activities, or (iii) is directly relevant to human consumption and/or production. Most importantly, any candidate entry must reflect an impact on some natural process. For example, a value quantifying the standing population of all livestock on Earth would fall under criterion (i) making it an appropriate candidate entry. As a counter example, the fraction of a country's GDP resulting from fossil fuel export would not be considered as a candidate value as it describes an economic impact rather than an impact on a natural process. Of course, rigid lines cannot always be drawn and inclusion of a value is ultimately at the discretion of the administration team.

Vetting a Potential Entry

Next, we determine if the quantity is scientifically valid and appropriate. This not only includes the precise value of the quantity, but the reliability of the source and the methods of measurement.

In general, we consider data from large, international efforts such as the Food and Agriculture Organization of the UN (FAO) or the Intergovernmental Panel on Climate Change (IPCC) to be highly reliable sources of information. We take these sources to be reliable as they clearly report the methods of their measurements or meta-analyses, emphasizing where assumptions and approximations have been made. Furthermore, given the internationality of its contributors and the deep well of scientists they consult and employ, we find that the FAO and IPCC are largely free of bias as they have little stake in reporting overly-rosy or negative results. For this reason, we are less likely to include values from industry reports, which have potential conflicts of interest. Whenever industry reports are used, we try to find multiple sources for that particular value to place it in context. For example, we extensively use the BP Annual Statistical Report on Energy in the human impacts database. As BP is a private company with financial interests in reporting global energy use, we compare these values with those from the US Energy

Information Administration (EIA) and the International Energy Agency (IEA) to judge their consistency.

We draw a large number of the values in the Human Impacts Database from peer-reviewed scientific reports. For these data sources we thoroughly examine the reported methods used to determine the value. If details regarding the method are not clearly reported (e.g. the value "was fitted" without explaining the fitting procedure), we are strongly inclined to not trust that particular source. Furthermore, if the method is not stated or the code/data are available under only "reasonable request", the value is not considered as appropriate. When possible, we also compare the reported value to other measurements and check if the source explains any discrepancy between their measurement and others. In many cases, however, there are not multiple reported values for a given quantity. In these cases, we assess the trustworthiness of the reported value and reach out to domain experts as needed. With rare exceptions, we do not factor the publishing journal in assessing the veracity of a value.

Once a value is entered into the database, we label it with a primary and secondary category. Human impacts are inherently connected by webs of interactions and often affect multiple subsystems within the Earth system. Meanwhile, most human impacts can be categorized according to the systems with which they interact most strongly. While incomplete, these category labels are meant to give users an impression of the subsystems that are most strongly influenced by or related to the value. Users are able to filter the database by these categories and subcategories.

Considering Uncertainty
While the numeric value of a candidate quantity is an important factor we consider, so too is the reported uncertainty. Many scientific reports will give an assessment of uncertainty, either at the statistical, measurement, or systematic level. The clarity of the presented uncertainty analysis is critical in our determination of whether a candidate value should be entered in the database. While scientific reports often address the uncertainty, this is rarely reported in governmental and industry reports. Many numbers from governmental or intergovernmental bodies come from surveys and are thus self-reported by countries, adding some uncertainty to the data and requiring some level of interpolation from the reporting agency. These numbers are still considered, though we are cognizant of the number of significant digits that are reported. Often, we report these numbers as approximate, representing the uncertainty with the data. In all cases, we state a concise yet sufficiently detailed description of the method and quantification of uncertainty in the "method" field of an HuID entry.

Considering Data Use Protections
As we do not directly generate the data presented in this work, we are very careful to ensure that the data we add to the database follows all legal requirements. All data presented in the database must be explicitly stated to be under a generally permissive license such as a Creative Commons Attribution license (CC-BY). Data sources which reserve all rights to their data are not included in the database in any form. While we ensure that we have the legal right to share

these data, we strongly implore the users of the Human Impacts Database to directly cite the original data source alongside the database if a value or entry is used in a later publication.

Continued Curation and Maintenance of the Database

Unlike similar databases in chemistry and biology (such as BioNumbers or the CRC Handbook of Chemistry and Physics), the Human Impacts Database faces a unique maintenance challenge as the values it houses will undoubtedly change with time as will our understanding of the facets of the Earth system that are impacted by human activities. This means that a concerted effort to keep the values in the database up to date, within reason, is needed. In this section, we outline steps we have taken to ensure that the database can be properly maintained and be useful for many years to come.

Composition of the Administrative Team

The primary authors on this work (GC, RAB, AIF, ILG, NSS, and MK) are the primary members of the administrative team of the Human Impacts Database. All of these authors are practicing research scientists working at the interface of biology, chemistry, physics, and earth science. As a result, this database will be an invaluable resource for our specific research objectives, imposing a self-interest in keeping the entries up to date. All members of the administrative team frequently read primary scientific literature covering these topics, meaning that critical new values or updates to extant entries can be reliably found. Furthermore, the majority of the administrative team intend to enter into leadership positions in academic and industry contexts, allowing us to mentor and train more administrators with different domain expertises. As this database is primarily a scientific tool, we believe our specific yet diverse training well prepares us as careful curators of the database. Furthermore, all authors are well-versed in computational methods with some administrators having expertise in web development technologies. This added expertise helps ensure that the database will reliably operate at both the front and backend levels. In addition, the two PIs who have led this work, RP and RM, have support from the Resnick Sustainability Center at Caltech and the Weizmann Institute to continue work on this project.

Many of the sources behind the HuID entries are updated on a regular basis, but updates may not be immediately updated on the database itself. For example, the FAO routinely updates their data as new data arrive or corrections/improvements to previously reported data are released. The frequent nature of these releases precludes a mirror reflection of these values in the Human Impacts Database. For continually updating sources, we update these values at an annual basis within the third quarter of the calendar year. Other sources, such as the BP statistical report on energy and IPCC reports, also typically release updates around this time. For values that are more frequently updated (such as the atmospheric CO_2 concentration, which is updated on a near-daily basis), we update these values semiannually coinciding with the spring and fall of the calendar year.

While the administration team is diverse in their scientific interests and expertise, it is unreasonable to believe that our collective knowledge is all-encompassing of Human Impacts. There will invariably be important values that we are unaware of that should be included in the database. To this end, we have developed a community-feedback system into the database (https://anthroponumbers.org/catalog/contact) where the general public can submit recommendations for new values or updates and/or corrections to extant values in the database. Whenever feedback is submitted, the administrative team is notified, preventing important feedback from being cast into the void. Furthermore, contact information is provided for each administrative member (https://anthroponumbers.org/catalog/about) if a user wishes to contact us individually.

As the curation procedures enumerated in the preceding sections are laborious and require a level of comfort in digesting scientific methods and data, we have opted to not open core maintenance privileges to the general public. However, all values housed within the database are also housed within a public GitHub repository (https://github.com/rpgroup-pboc/human_impacts) where we enthusiastically encourage forking of the repository and submission of new issues and pull requests. The issues and pull requests are also monitored by the administrative team.

Supplemental Note 2: References and Explanations For Values Reported in Figure 1

In this section, we report our extensive and detailed referencing for each and every quantity reported in the subpanels of Figure 1 of the main text. As described in the Materials & Methods, each value comes from the manual curation of a piece of scientific, industrial, governmental, or non-governmental organization reports, articles, or databases. Each value listed here contains information about the original source, the method used to obtain the value, as well as accession identification numbers for the Human Impacts Database (https://anthroponumbers.org), listed as HuIDs.

For each value, we attempt to provide an assessment of the uncertainty. For some values, this corresponds to the uncertainty in the measurement or inference as stated in the source material. In cases where a direct assessment of the uncertainty was not clearly presented, we sought other reported values for the same quantity from different data sources to present a range of the values. For others, this uncertainty represents the upper- and lower-bounds of the measurement or estimation.

Each value reported here is prefixed with a symbol representing our confidence in the value. A symbol of equality (=) represents that either i) the value is known within a measurable uncertainty or b) multiple sources confirm this value. A symbol of approximation (≈) represents that we are confident in the reported value within a factor of a multiplicative factor less than 10. In some cases, an approximation symbol (≈) represents a range where the values from different sources differ within three significant digits and the range is then reported. Some values in the database are only known with a lower-bound limit. In these cases, the value is reported with an inequality symbol (>).

A. Surface Warming

Surface temperature change from the 1850-1900 average ≈ 1.0 - 1.3 ℃ (HuID: 79598, 76539, 12147)

Data Source(s): HadCRUT.4.6 (Morice et al., 2012, DOI: 10.1029/2011JD017187), GISTEMP v4 (GISTEMP Team, 2020: GISS Surface Temperature Analysis (GISTEMP), version 4. NASA Goddard Institute for Space Studies. Dataset accessed 2020-12-17 at https://data.giss.nasa.gov/gistemp/ & Lenssen et al., 2019, DOI: 10.1029/2018JD029522) and NOAAGlobalTemp v5 (Huang et al, 2020, DOI: 10.1175/JCLI-D-19-0395.1) datasets.

Notes: The global mean surface temperature captures near-surface air temperature over the planet's land and ocean surface. The value reported represents the spread of three estimates and their 95% confidence intervals for the year 2019. Since data for the period 1850-1880 are missing in GISTEMP v4 and NOAAGlobalTemp v5, data are centered by setting the 1880-1900 mean of all datasets to the HadCRUT.4.6 mean over the same period.

B. Annual Ice Melt

Glaciers = (3.0 ± 1.2) × 10^{11} m^3 / yr (HuID: 32459)

Data Sources: Intergovernmental Panel on Climate Change (IPCC) 2019 Special Report on the Ocean and Cryosphere in a Changing Climate. Table 2.A.1 on pp. 199-202.

Notes: Value corresponds to the trend of annual glacial ice volume loss (reported as ice mass loss) from major glacierized regions (2006-2015) based on aggregation of observation methods (original data source: Zemp et al. 2019, DOI:10.1038/s41586-019-1071-0) with satellite gravimetric observations (original data source: Wouters et al. 2019, DOI:10.3389/feart.2019.00096). Ice volume loss was calculated from ice mass loss assuming a standard pure ice density of 920 kg / m^3. Uncertainty represents a 95% confidence interval calculated from standard error propagation of the 95% confidence intervals reported in the original sources assuming them to be independent.

Ice sheets = (4.6 ± 0.4) × 10^{11} m^3 / yr (HuIDs: 95798; 93137)

Data Source(s): D. N. Wiese et al. 2019 JPL GRACE and GRACE-FO Mascon Ocean, Ice, and Hydrology Equivalent HDR Water Height RL06M CRI Filtered Version 2.0, Ver. 2.0, PO.DAAC, CA, USA. Dataset accessed [2022-Feb-09]. DOI: 10.5067/TEM- SC-3MJ62

Notes: Value corresponds to the trends of combined annual ice volume loss (reported as ice mass loss) from the Greenland and Antarctic Ice Sheets (2002-2021) measured by satellite gravimetry. Ice volume loss was calculated from ice mass loss assuming a standard pure ice density of 920 kg / m^3. Uncertainty represents one standard deviation and considers only propagation of monthly uncertainties in measurement.

Arctic sea ice = (3.0 ± 1.0) × 10^{11} m^3 / yr (HuID: 89520)

Data Source(s): PIOMAS Arctic Sea Ice Volume Reanalysis, Figure 1 of webpage as of January 31, 2022. Original method source: Schweiger et al. 2011, DOI:10.1029/2011JC007084

Notes: Value reported corresponds to the trend of annual volume loss from Arctic sea ice (1979-2022). The uncertainty in the trend represents the range in trends calculated from three ice volume determination methods.

C. Sea Ice Area

Extent of loss at yearly maximum cover (September) ≈ 4.8 × 10^{10} m^2 / yr (HuID: 66277)
Extent loss at yearly minimum cover (March) ≈ 0.4 × 10^{10} m^2 / yr (HuID: 66277)
Average annual extent loss = 2.5 × 10^{10} m^2 / yr (HuID: 66277)

Data Source(s): Fetterer et al. 2017, updated daily. Sea Ice Index, Version 3, Boulder, Colorado USA. NSIDC: National Snow and Ice Data Center, DOI:10.7265/N5K072F8, [Accessed 2022-Feb-16].
Notes: Sea ice area is calculated by multiplying the percentage of sea ice in each pixel by pixel area and taking the integral sum of these products. Annual value corresponds to the linear trend of annual extent loss calculated by averaging over every month in a given year (2.45×10^{10} m^2 / yr HuID: 66277). The minimum cover area loss corresponds to the linear trend of Arctic sea ice area in September from 1979-2021 and the maximum cover area loss corresponds to the linear trend of sea ice area in March from 1979-2021. The Antarctic sea ice area trend is not shown because a significant long-term trend over the satellite observation period is not observed and short-term trends are not yet identifiable.

D. Annual Material Production

Concrete production ≈ (2 - 3) × 10^{13} kg / yr (HuID: 25488; 81346; 16995)
Data Source(s): United States Geological Survey (USGS) National Minerals Information Center, Commodity Statistics and Information, Cement Statistics and Information. Miller et al. 2016, Table 1, DOI:10.1088/1748-9326/11/7/074029. Monteiro et al. 2017, DOI:10.1038/nmat4930. Krausmann et al. 2017, DOI:10.1073/pnas.1613773114
Notes: Concrete is formed when aggregate material is bonded together by hydrated cement. The USGS reports the mass of cement produced in 2019 as 4.1×10^{12} kg. As most cement is used to form concrete, cement production can be used to estimate concrete mass using a multiplicative conversion factor of 7 (Monteiro et al.). Miller et al. report that the cement, aggregate and water used in concrete in 2012 sum to 2.3×10^{13} kg. Krausmann et al. report an estimated value from 2010 based on a material input, stocks, and outputs model. The value is net annual addition to concrete stocks plus annual waste and recycling to estimate gross production of concrete.

Steel production ≈ 1.9 × 10^{12} kg / yr (HuID: 51453; 44894; 85981)
Data Source(s): United States Geological Survey (USGS) National Minerals Information Center, Commodity Statistics and Information, Iron and Steel Statistics and Information. World Steel Association, World Steel in Figures 2021, p. 7. Krausmann et al. 2017, DOI:10.1073/pnas.1613773114
Notes: Crude steel includes stainless steels, carbon steels, and other alloys. The USGS reports the mass of crude steel produced in 2019 as 1.860×10^{12} kg. The World Steel Association reports a production value of 1.874×10^{12} kg in 2019. Krausmann et al. report an estimated value from 2010 based on a material input, stocks, and outputs model. The value is net annual addition to steel stocks plus annual waste and recycling to estimate gross production of steel.

Plastic production ≈ 4 × 10^{11} kg / yr (HuID: 97241; 25437)
Data Source(s): Geyer et al. 2017, Table S1, DOI:10.1126/sciadv.1700782. Krausmann et al. 2017, DOI:10.1073/pnas.1613773114
Notes: Value represents the approximate sum total global production of plastic fibers and plastic resin during the calendar year of 2015. Comprehensive data about global plastic production is sorely lacking. Geyer et al. draw data from various industry groups to estimate total production of different polymers and additives. Some of the underlying data is not publicly available, and data from financially-interested parties is inherently suspect. Krausmann et al. report an estimated value from 2010 based on a material input, stocks, and outputs model. The value is net annual addition to stocks plus annual waste and end-of-life recycling to estimate gross production of plastics.

E. Livestock Population

Chicken standing population ≈ 3.5 × 10^{10} (HuID: 94934)

Cattle standing population ≈ 1.5 × 10^9 (HuID: 92006)
Swine standing population ≈ 1.5 × 10^9 (HuID: 21368)
All livestock standing population ≈ 4.6 × 10^{10} (HuID: 43599)
Data Source(s): Food and Agriculture Organization (FAO) of the United Nations Statistical Database (2022) — Live Animals.
Notes: Counts correspond to the estimated standing populations in 2019. Values are reported directly by countries. The FAO uses non-governmental statistical sources to address uncertainty and missing (non-reported) data. Reported values are therefore approximations.

F. Annual Synthetic Nitrogen Fixation

Annual mass of synthetically fixed nitrogen ≈ (1.4 - 1.5) × 10^{11} kg N / yr (HuID: 60580; 61614)
Data Source(s): United States Geological Survey (USGS) National Minerals Information Center, Commodity Statistics and Information, Nitrogen Statistics and Information. International Fertilizer Association (IFA) Statistical Database (2021) — Ammonia Production & Trade Tables by Region. Smith et al. 2020, DOI: 10.1039/c9ee02873k.
Notes: Ammonia (NH_3) produced globally is compiled by the USGS and IFA from major factories that report output. The USGS estimates the approximate mass of nitrogen in ammonia produced in 2019 as 1.42 × 10^{11} kg N and the International Fertilizer Association reports a production value of 1.50 × 10^{11} kg N in 2019. Nearly all of this mass is produced by the Haber-Bosch process (>96%, Smith et al. 2020). In the United States most of this mass is used for fertilizer, with the remainder being used to synthesize nitrogen-containing chemicals including explosives, plastics, and pharmaceuticals (≈ 88%, USGS Mineral Commodity Summaries 2020 – Nitrogen).

G. Ocean Acidity

Surface ocean [H+] ≈ 0.2 parts per billion (HuID: *90472*)
Annual change in [H+] = *0.36 ± 0.03% (HuID: 19394)*
Data Source(s): Figures 1-2 of European Environment Agency report CLIM 043 (2020). Original data source of the report is “Global Mean Sea Water pH” from Copernicus Marine Environment Monitoring Service.
Notes: Reported value is calculated from the global average annual change in pH over years 1985-2018. The average oceanic surface pH was ≈ 8.057 in 2018 and decreases annually by ≈ 0.002 units, giving a change in [H+] of roughly $10^{-8.055}$ - $10^{-8.057}$ ≈ 4x10^{-11} mol/L or about 0.4% of the global average. [H+] is calculated as 10^{-pH} ≈ 10^{-8} mol/L or 0.2 parts per billion (ppb), noting that [H_2O] ≈ 55 mol/L. Uncertainty for annual change is the standard error of the mean.

H. Land Use

Agriculture ≈ 5 × 10^{13} m^2 (HuID: 29582)
Data Source(s): Food and Agriculture Organization (FAO) of the United Nations Statistical Database (2020) — Land Use.
Notes: Agricultural land is defined as all land that is under agricultural management including pastures, meadows, permanent crops, temporary crops, land under fallow, and land under agricultural structures (such as barns). Reported value corresponds to 2017 estimates by the FAO.
Urban ≈ (6 - 8) × 10^{11} m^2 (HuID: 41339; 39341)
Data Source(s): Florczyk et al. 2019 (https://tinyurl.com/yyxxgtll) and Table 3 of Liu et al. 2018 DOI: 10.1016/j.rse.2018.02.055
Notes: Urban land area is determined from satellite imagery. An area is determined to be “urban” if the total population is greater than 5,000 and has a minimum population density of 300 people per km^2. Reported

value gives the range of recent measurements of ≈ 6.5×10^{11} m^2 (2015) and ≈ (7.5 ± 1.5) $\times10^{11}$ m^2 (2010) from Florczyk et al. 2019 and Liu et al. 2018, respectively.

I. River Fragmentation

Global fragmented river volume ≈ 6×10^{11} m^3 (HuID: 61661)

Data Source(s): Grill et al. 2019 DOI: 10.1038/s41586-019-1111-9

Notes: Value corresponds to the water volume contained in rivers that fall below the connectivity threshold required to classify them as free-flowing. Value considers only rivers with upstream catchment areas greater than 10 km^2 or discharge volumes greater than 0.1 m^3 per second. The ratio of global river volume in disrupted rivers to free-flowing rivers is approximately 0.9. The exact value depends on the cutoff used to define a "free-flowing" river. We direct the reader to the source for thorough detail.

J. Human Population

Urban population ≈ 55% (HuID: 93995)

Global population ≈ 7.6×10^9 people (HuID: 85255)

Data Source(s): Food and Agricultural Organization (FAO) of the United Nations Report on Annual Population, 2019.

Notes: Value for total population in 2018 comes from a combination of direct population reports from country governments as well as inferences of underreported or missing data. The definition of "urban" differs between countries and the data does not distinguish between urban and suburban populations despite substantive differences between these land uses (Jones & Kammen 2013, DOI: 10.1021/es4034364). As explained by the United Nations population division, "When the definition used in the latest census was not the same as in previous censuses, the data were adjusted whenever possible so as to maintain consistency." Rural population is computed from this fraction along with the total human population, implying that the total population is composed only of "urban" and "rural" communities.

K. Greenhouse Gas Emissions

Anthropogenic CO_2 = (4.25 ± 0.33) × 10^{13} kg CO_2 / yr (HuID: 24789; 54608; 98043; 60670)

Data Source(s): Table 6 of Friedlingstein et al. 2019, DOI: 10.5194/essd-11-1783-2019. Original data sources relevant to this study compiled in Friedlingstein et al.: 1) Gilfillan et al. https://energy.appstate.edu/CDIAC 2) Average of two bookkeeping models: Houghton and Nassikas 2017 DOI: 10.1002/2016GB005546; Hansis et al. 2015 DOI: 10.1002/2014GB004997. 3) Dlugokencky and Tans, National Oceanic & Atmospheric Administration, Earth System Research Laboratory (NOAA/ESRL), https://www.esrl.noaa.gov/gmd/ccgg/trends/global.html, [Accessed 3-Nov-2019].

Notes: Value corresponds to total CO_2 emissions from fossil fuel combustion, industry (predominantly cement production), and land-use change during calendar year 2018. Emissions from land-use change are due to the burning or degradation of plant biomass. In 2018, roughly 1.88 × 10^{13} kg CO_2 / yr accumulated in the atmosphere, reflecting the balance of emissions and CO_2 uptake by plants and oceans (Dlugokencky and Tans). Uncertainty corresponds to one standard deviation.

Anthropogenic CH_4 = (3.4 - 3.9) × 10^{11} kg CH_4 / yr (HuID: 96837; 30725)

Data Source(s): Table 3 of Saunois, et al. 2020. DOI: 10.5194/essd-12-1561-2020.

Notes: Value corresponds to 2008-2017 decadal average mass of CH_4 emissions from anthropogenic sources. Includes emissions from agriculture and landfill, fossil fuels, and burning of biomass and biofuels, but

other inventories of anthropogenic methane emissions are also considered. Reported range represents the minimum and maximum estimated emissions from a combination of “bottom-up” and “top-down” models.

Anthropogenic N_2O = 1.1 (+0.6, -0.5) × 10^{10} kg N_2O / yr (HuID: 44575)

Data Source(s): Table 1 of Tian, H., et al. 2020. DOI: 10.1038/s41586-020-2780-0

Notes: Value corresponds to annualized N_2O emissions from anthropogenic sources in the years 2007-2016. The value reported in the source is 7.3 [4.2, 11.4] Tg N / year. This is converted to a mass of N_2O using the fact that N ≈ 14/22 of the mass of N_2O. Reported value is mean with the uncertainty bounds (+,-) representing the maximum and minimum values observed in the 2007-2016 time period.

L. Water Withdrawal

Agricultural = 1.3 × 10^{12} m^3 / year (HuID: 84545, 43593, 95345)

Industrial = 5.9 × 10^{11} m^3 / year (HuID: 27142)

Domestic = 5.4 × 10^{10} m^3 / year (HuID: 69424)

Total = (1.7 - 2.2) × 10^{12} m^3 / year (HuID: 27342, 68004)

Data Source(s): Figure 1 of Qin et al. 2019. DOI: 10.1038/s41893-019-0294-2. AQUASTAT Main Database, Food and Agriculture Organization of the United Nations

Notes: “Agricultural” and “total” withdrawal include one value from Qin et al. (who reports “consumption”) and one value from the AQUASTAT database. Industrial water withdrawal is from AQUASTAT and domestic withdrawal value is from Qin et al. Values in AQUASTAT are self-reported by countries and have missing values from some countries, probably accounting for a few percent underreporting. All values represent water withdrawals. For agricultural and domestic, water withdrawal is assumed to be the same as water consumption, which is reported in Qin et al.

M. Sea Level Rise

Added water = 1.97 (+0.36, -0.34) mm / yr (HuID: 97108)

Thermal expansion = 1.19 (+0.25, -0.24) mm / yr (HuID: 97688)

Total observed sea-level rise = 3.35 (+0.47, -0.44) mm / yr (HuID: 81373)

Data Source(s): Table 1 of Frederikse et al. 2020. DOI:10.1038/s41586-020-2591-3.

Notes: Values correspond to the average global sea level rise for the years 1993 - 2018. “Added water” (barystatic) change includes effects from meltwater from glaciers and ice sheets, added mass from sea-ice discharge, and changes in the amount of terrestrial water storage. Thermal expansion accounts for the volume change of water with increasing temperature. Values for “thermal expansion” and “added water” come from direct observations of ocean temperature and gravimetry/altimetry, respectively. Total sea level rise is the observed value using a combination of measurement methods. “Other sources” reported in Figure 1 accounts for observed residual sea level rise not attributed to a source in the model. Values in brackets correspond to the upper and lower bounds of the 90% confidence interval.

N. Total Power Use

Global power use ≈ 19 - 20 TW (HuID: 31373; 85317)

Data Source(s): bp Statistical Review of World Energy, 2020; U.S. Energy Information Administration, 2020.

Notes: Value represents the sum of total primary energy consumed from oil, natural gas, coal, and nuclear energy and electricity generated by hydroelectric and other renewables. Value is calculated using annual primary energy consumption as reported in data sources assuming uniform use throughout a year, yielding ≈ 19 - 20 TW.

O. Tree Coverage Area Loss

Commodity-driven deforestation = (5.7 ± 1.1) × 10^{10} m^2 / yr (HuID: 96098)
Forestry = (5.4 ± 0.8) × 10^{10} m^2 / yr (HuID: 38352)
Urbanization = (2 ± 1) × 10^9 m^2 / yr (HuID: 19429)
Shifting agriculture = (7.5 ± 0.9) × 10^{10} m^2 / yr (HuID: 24388)
Wildfire = (7.2 ± 1.3) × 10^{10} m^2 / yr (HuID: 92221)
Total tree cover area loss ≈ 2 × 10^{11} m^2 / yr (HuID: 78576)
Data Source(s): Table 1 of Curtis et al. 2018 DOI:10.1126/science.aau3445. Hansen et al. 2013 DOI:10.1126/science.1244693. Global Forest Watch, 2020. Reported values in source correspond to total loss from 2001 - 2015. Values given are averages over this 15 year window.
Notes: Commodity-driven deforestation is "long-term, permanent, conversion of forest and shrubland to a non-forest land use such as agriculture, mining, or energy infrastructure." Forestry is defined as large-scale operations occurring within managed forests and tree plantations with evidence of forest regrowth in subsequent years. Urbanization converts forest and shrubland for the expansion and intensification of existing urban centers. Disruption due to "shifting agriculture" is defined as "small- to medium-scale forest and shrubland conversion for agriculture that is later abandoned and followed by subsequent forest regrowth". Disruption due to wildfire is "large-scale forest loss resulting from the burning of forest vegetation with no visible human conversion or agricultural activity afterward." Uncertainty corresponds to the reported 95% confidence interval. Uncertainty is approximate for "urbanization" as the source reports an ambiguous error of "± <1%."

P. Power From Fossil Fuels

Natural gas = 4.5 - 4.9 TW (HuID: 49947; 86175)
Oil = 6.1 - 6.6 TW (HuID: 42121; 39756)
Coal = 5.0 - 5.6 TW (HuID: 10400; 60490)
Total = 16 - 17.0 TW (HuID: 29470; 29109)
Data Source(s): bp Statistical Review of World Energy, 2020. U.S. Energy Information Administration, 2022.
Notes: Values are self-reported by countries. All values from bp Statistical Review and EIA correspond to 2019.. Reported TW values are computed from primary energy units (e.g. kg coal) assuming uniform use throughout the year. Oil volume includes crude oil, shale oil, oil sands, condensates, and natural gas liquids separate from specific natural gas mining. Natural gas value excludes gas flared or recycled and includes natural gas produced for gas-to-liquids transformation. Coal value includes 2019 value exclusively for solid commercial fuels such as bituminous coal and anthracite, lignite and subbituminous coal, and other solid fuels. This includes coal used directly in power production as well as coal used in coal-to-liquids and coal-to-gas transformations.

Q. Power From Renewable Resources

Wind = 0.36 - 0.43 TW (HuID: 30581, 85919)
Solar = 0.18 - 0.21 TW (HuID: 99885, 58303)
Hydroelectric = 1.2 - 1.3 TW (HuID: 15765, 50558)
Total = 1.9 - 2.1 TW (HuID: 74571, 20246)
Data Source(s): bp Statistical Review of World Energy, 2020. U.S. Energy Information Administration, 2022.
Notes: Reported values correspond to estimates for the 2019 calendar year. Renewable resources are defined as wind, geothermal, solar, biomass and waste. Hydroelectric, while presented here, is not defined as

a renewable in the BP dataset. All values are reported as input-equivalent energy, meaning the input energy that would have been required if the power was produced by fossil fuels. BP reports that fossil fuel efficiency used to make this conversion was about 40% in 2017.

R. Fossil Fuel Extraction

Natural gas volume = (3.9 - 4.0) × 10^{12} m^3 / yr (HuID: 11468; 20532)
Oil volume = (5.5 - 5.8) × 10^9 m^3 / yr (HuID: 66789; 97719)
Coal mass = (7.8 - 8.1) × 10^{12} kg / yr (HuID: 78435; 48928)
Data Source(s): bp Statistical Review of World Energy, 2020. U.S. Energy Information Administration (EIA), 2022.
Notes: Oil volume includes crude oil, shale oil, oil sands, condensates, and natural gas liquids separate from specific natural gas mining. Natural gas value excludes gas flared or recycled and includes natural gas produced for gas-to-liquids transformation. Coal value includes solid commercial fuels such as bituminous coal, anthracite, lignite, subbituminous coal, and other solid fuels. All values correspond to 2019 estimates..

S. Ocean Warming

Heat uptake = $346 \pm 51\, TW$ (HuID: 94108)
Upper ocean (0 - 700m) temperature increase since to 1960 = $0.18 - 0.20$ ℃ (HuID: 69674, 72086)
Data Source(s): Table S1 of Cheng et al. 2017. DOI: 10.1126/sciadv.1601545. NOAA National Centers for Environmental Information, 2020. DOI: 10.1029/2012GL051106.
Notes: Heat uptake reported is the average over time period 1992-2015 with 95% confidence intervals. Range of temperatures reported captures the 95% confidence interval of temperature increase for the period 2015-2019 with respect to the 1958-1962 mean. Temperature change is considered in the upper 700 m because sea surface temperatures have high decadal variability and are a poor indicator of ocean warming; see Roemmich et al. 2015, DOI: 10.1038/NCLIMATE2513.

T. Power From Nuclear Fission

Nuclear power ≈ 0.79 - 0.92 TW (HuID: 48387; 71725)
Data Source(s): bp Statistical Review of World Energy, 2020. U.S. Energy Information Administration (EIA), 2022
Notes: Values are self-reported by countries and correspond to estimates for the 2019 calendar year.. Values are reported as 'input-equivalent' energy, meaning the energy that would have been needed to produce a given amount of power if the input were a fossil fuel, which is converted to TW here. This is calculated by multiplying the given power by a conversion factor representing the efficiency of power production by fossil fuels. In 2017, this factor was about 40%.

U. Nuclear Fallout

Anthropogenic ^{239}Pu and ^{240}Pu from nuclear weapons ≈ 1.4 × 10^{11} kg / yr (HuID: 42526)
Data Source(s): Table 1 in Hancock et al. 2014 doi: 10.1144/SP395.15. Fallout in activity from UNSCEAR 2000 Report on Sources and Effects of Ionizing Radiation Report to the UN General Assembly -- Volume 1.
Notes: The approximate mass of Plutonium isotopes ^{239}Pu and ^{240}Pu released into the atmosphere from the ≈ 500 above-ground nuclear weapons tests conducted between 1945 and 1980. Naturally occurring ^{239}Pu and ^{240}Pu are rare, meaning that nearly all contemporary labile plutonium comes from human production (Taylor 2001,doi: 10.1016/S1569-4860(01)80003-6). The total mass of radionuclides released is ≈ 3300 kg with a

combined radioactive fallout of ≈ 11 PBq. These values do not represent the entire 239+240Pu globally distributed mass as it excludes non-weapons sources.

V. Contemporary Extinction

Animal species extinct since 1500 > 750 (HuID: 44641)
Plant species extinct since 1500 > 120 (HuID: 86866)
Data Source(s): The IUCN Red List of Threatened Species. Version 2020-2
Notes: Values correspond to absolute lower-bound count of animal extinctions over the past ≈ 520 years. Of the predicted ≈ 8 million animal species, the IUCN databases catalogues only ≈ 900,000 with only ≈ 75,000 being assigned a conservation status. Representation of plants and fungi is even more sparse with only ≈ 40,000 and ≈ 285 being assigned a conservation status, respectively. The number of extinct animal species is undoubtedly higher than these reported values, as signified by an inequality symbol (>).

W. Earth Moving

Waste and overburden from coal mining ≈ 6.5 × 10^{13} kg / yr (HuID: 72899)
Earth moved from urbanization > 1.4 × 10^{14} kg / yr (HuID: 59640)
Data Source(s): Supplementary table 1 of Cooper et al. 2018. DOI: doi.org/gfwfhd.
Notes: Coal mining waste and overburden mass is calculated given commodity-level stripping ratios (mass of overburden/waste per mass of coal resource mined) and reported values of global coal production by type. Urbanization mass is presented as a lower bound estimate of the mass of earth moved from global construction projects. This comes from a conservative estimate that the ratio of the mass of earth moved per mass of cement/concrete used in construction globally is 2:1. This value is highly context dependent and we encourage the reader to read the source material for a more thorough description of this estimation.
Erosion rate from agriculture > (1.2 - 2.4) × 10^{13} kg / yr (HuID: 19415; 41496)
Data Source(s): Pg. 377 of Wang and Van Oost 2019. DOI: 10.1177/0959683618816499. Pg. 21996 of Borrelli et al. 2020 DOI: 10.1073/pnas.2001403117.
Notes: Cumulative sediment mass loss over history of human agriculture due to accelerated erosion is estimated to be ≈ 30,000 Gt. Recent years have an estimated erosion rate ranging from 12 Pg / yr (Wang and Van Oost) to ≈ 24 Pg / yr (Borrelli et al.). Values come from computational models conditioned on time-resolved measurements of sediment deposition in catchment basins.
